# Supplementary material for: The Heterogeneity of Ly6Chi Monocytes Controls Their Differentiation into iNOS+ Macrophages or Monocyte-Derived Dendritic Cells
Source: Immunity. 2016 Dec 20;45(6):1205–18. doi: 10.1016/j.immuni.2016.12.001 (PMC5196026; doi:10.1016/j.immuni.2016.12.001)
Supplement: Document S1. Supplemental Experimental Procedures and Figures S1–S7 [file mmc1.pdf]

Immunity, Volume *45*

Supplemental Information

The Heterogeneity of Ly6C^{hi} Monocytes Controls Their Differentiation into iNOS^{+} Macrophages or Monocyte-Derived Dendritic Cells

Shinelle Menezes, Daisy Melandri, Giorgio Anselmi, Thibaut Perchet, Jakob Loschko, Juan Dubrot, Rajen Patel, Emmanuel L. Gautier, Stéphanie Hugues, M. Paula Longhi, Jake Y. Henry, Sergio A. Quezada, Grégoire Lauvau, Ana-Maria Lennon-Duménil, Enrique Gutiérrez-Martínez, Alain Bessis, Elisa Gomez-Perdiguero, Christian E. Jacome-Galarza, Hannah Garner, Frederic Geissmann, Rachel Golub, Michel C. Nussenzweig, and Pierre Guermonprez

FIGURE S1

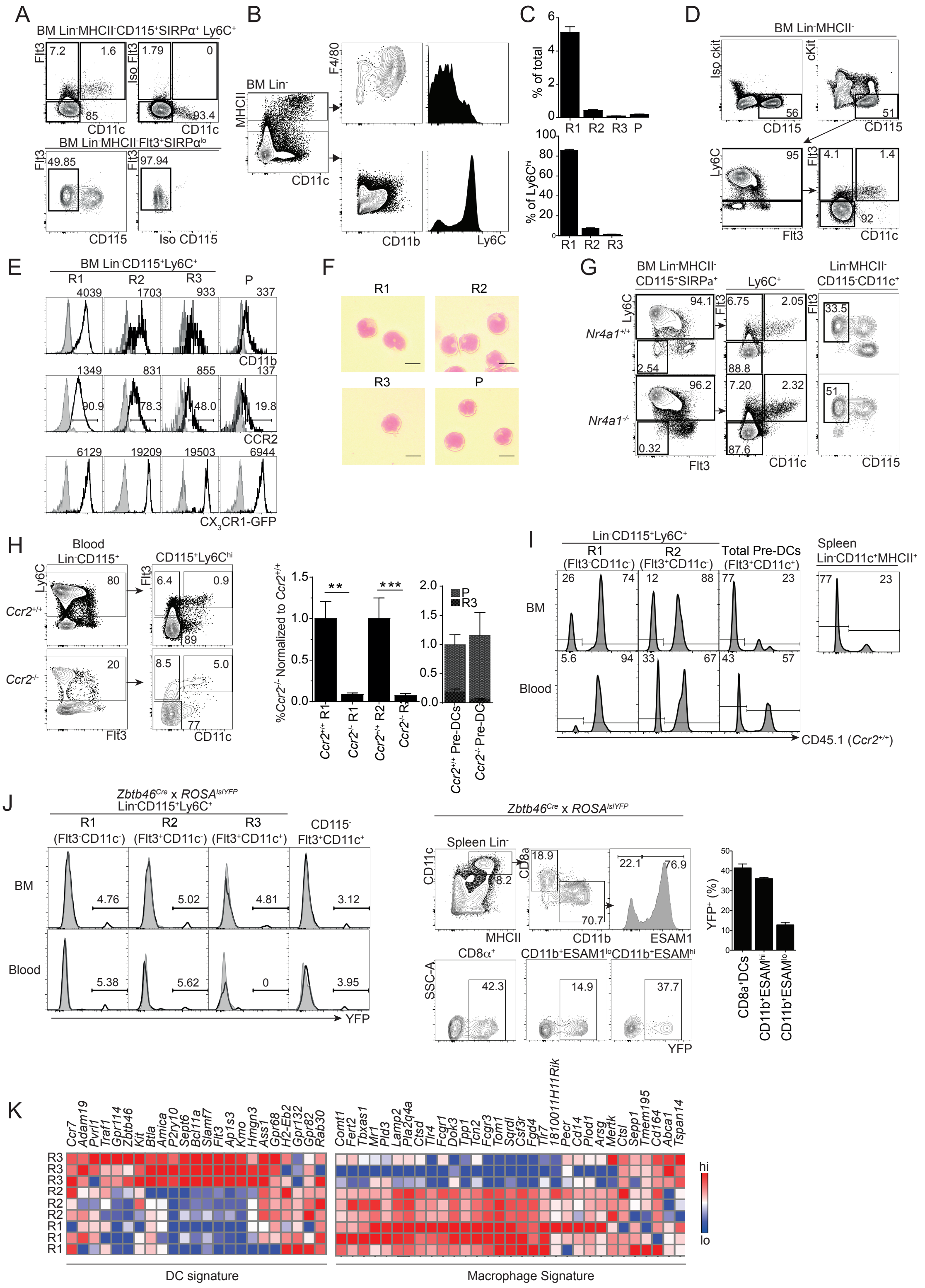

A
BM Lin-MHCII-CD115+SIRPα+ Ly6C+
BM Lin-MHCII-Flt3+SIRPαlo
B
BM Lin-
C
D
BM Lin-MHCII-
E
BM Lin-CD115+Ly6C+
F
G
Nr4a1+/+
Nr4a1-/-
H
Blood Lin-CD115+
CD115+Ly6Chi
Ccr2+/+
Ccr2-/-
I
Lin-CD115+Ly6C+
Total Pre-DCs
Spleen Lin-CD11c+MHCII+
CD45.1 (Ccr2+/+)
J
Zbtb46Cre x ROSAlslYFP
K
DC signature
Macrophage Signature

Figure S1 (Related to Figure 1): Phenotypic characterization of R1 and R2 monocytes and R3 and P pre-DCs.

A) FACS analysis of Flt3 and CD115 in BM $Ly6C^{+}$ cells.

Anti-Flt3 or isotype staining on $MHCII^{-}CD115^{+}SIRP\alpha^{+}Ly6C^{+}$ R1 and R2 monocytes and R3 pre-DCs, and anti-CD115 or isotype staining on R3 and P ($Lin^{-}MHCII^{-}CD115^{-}Flt3^{+}CD11c^{+}$) pre-DCs gated on Lin^{-}($Ly6G^{-}$, $CD3e^{-}$.$NK1.1^{-}$, $Ter119^{-}$, $CD19^{-}$, $CD45RA^{-}$) cells.

B) Phenotype of $MHCII^{+}$ cells in the BM. F4/80, CD11b and Ly6C expression

on WT BM $Lin^{-}MHCII^{+}$ and $MHCII^{-}$ cells.

C) Cellularity of BM subsets. Percentage of R1, R2 monocytes and R3 pre-DCs in total BM and in $Lin^{-}CD115^{+}Ly6C^{+}$ cells and of $CD115^{-}$pre-DC (P) in total BM.

D) R1, R2 and R3 are not $ckit^{+}$.

Anti-ckit or isotype control staining of BM $Lin^{-}MHCII^{-}$ cells. R1 and R2 monocytes and R3 pre-DCs are gated from Lin^{-}($CD3\varepsilon^{-}CD19^{-}Ly6G^{-}CD45RA^{-}NK1.1^{-}Ter119^{-}$) $MHCII^{-}ckit^{-}CD115^{+}Ly6C^{+}$ cells.

E) Phenotype of BM R1 and R2 monocytes and pre-DCs R3 and P. FACS analysis for CD11b and CCR2 (solid black lines) expression versus isotype controls (grey shaded) in WT mice. GFP reporter expression analyzed in *Cx3cr1*$^{gfp/+}$ mice at steady state. Numbers above gates indicate percentage of parent population and numbers above histograms indicate mean fluorescence intensity.

F) Giemsa staining of FACS sorted R1, R2 monocytes and R3 and P Pre-DCs from the BM of WT mice. Scale bar=10µm.

G) *Nr4a1* dependency of R1, R2, R3 and P in WT mice. Representative FACS analysis of R1 and R2 monocyte subsets and pre-DCs- R3 and P, in *Nr4a1*$^{+/+}$ and *Nr4a1*$^{-/-}$ BM.

H) Analysis of monocytes R1 and R2, and R3 pre-DCs in the blood of *Ccr2*$^{-/-}$ versus WT mice at steady state.

I) FACS analysis of *Ccr2*$^{-/-}$ and WT mixed bone marrow chimeras. FACS analysis of CD45.1^{+} (*Ccr2*$^{+/+}$) and CD45.1^{-}(*Ccr2*$^{-/-}$) cells within R1, R2 monocytes and total pre-DCs in the BM and blood, and total cDCs in the spleen of WT reconstituted recipients of *Ccr2*$^{-/-}$ and WT mixed BM (1:1).

J) Fate mapping of R1, R2, R3 and P in *Zbtb46*^{Cre} *x ROSA*^{lslYFP} mice. YFP expression in R1, R2 monocytes and pre-DCs R3 and P, in the BM and blood, and CD8α^{+} and CD11b^{+}ESAM^{+} and ESAM^{-} cDCs in the spleens of *Zbtb46*^{Cre} *x ROSA*^{lslYFP} mice.

K) Expression of cDC and macrophage ImmGen gene signatures in BM R1, R2 and R3 at steady state. Relative mRNA expression levels for each gene are depicted according to the color bar shown.

FIGURE S2

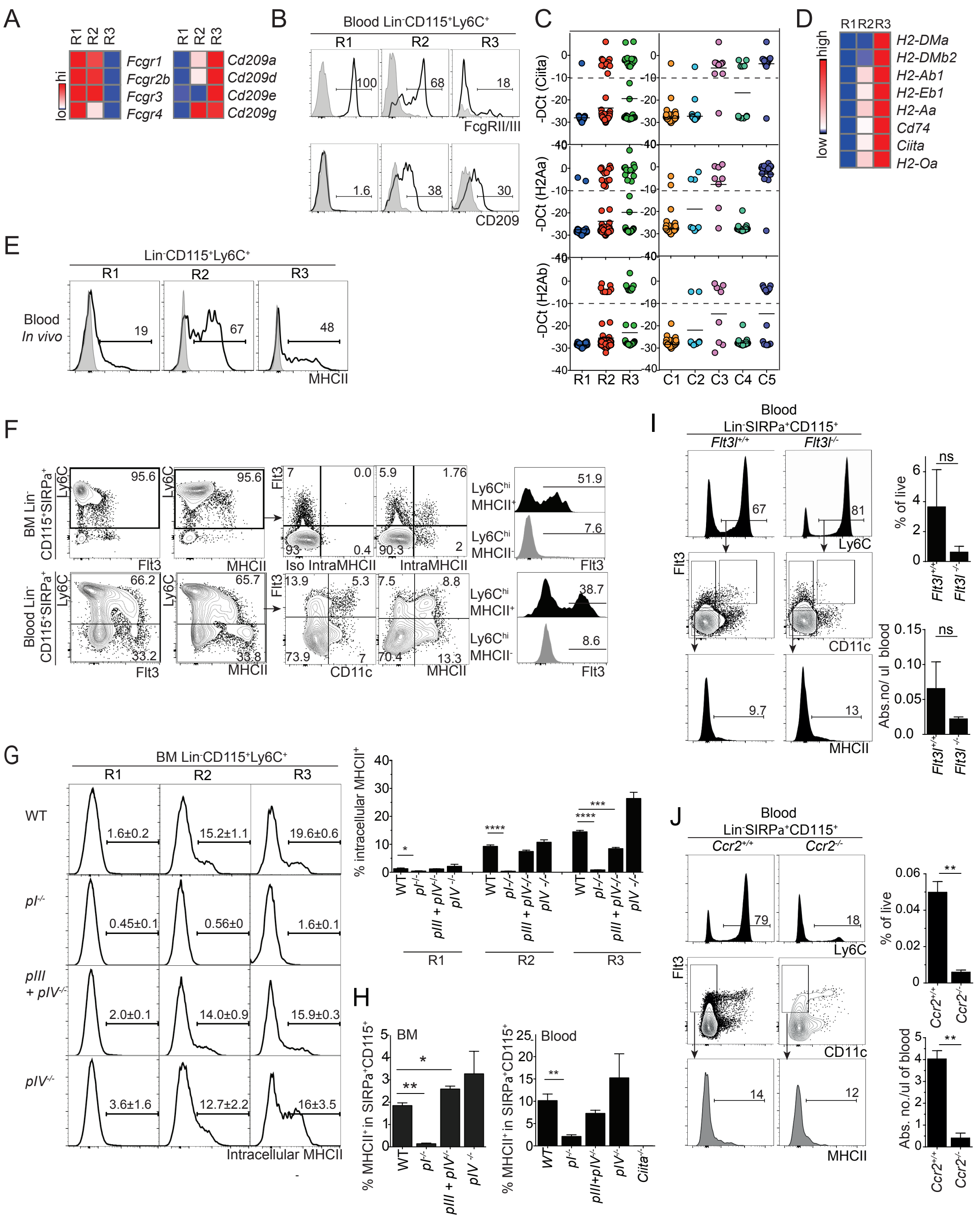

A
Fcgr1
Fcgr2b
Fcgr3
Fcgr4
Cd209a
Cd209d
Cd209e
Cd209g
B
Blood Lin-CD115+Ly6C+
FcgRII/III
CD209
C
-DCt (Ciita)
-DCt (H2Aa)
-DCt (H2Ab)
D
H2-DMa
H2-DMb2
H2-Ab1
H2-Eb1
H2-Aa
Cd74
Ciita
H2-Oa
E
Lin-CD115+Ly6C+
Blood
In vivo
MHCII
F
BM Lin-CD115+SIRPa+
Blood Lin-CD115+SIRPa+
Iso IntraMHCII
IntraMHCII
G
BM Lin-CD115+Ly6C+
WT
pI-/-
pIII + pIV-/-
pIV-/-
Intracellular MHCII
% intracellular MHCII+
H
BM
Blood
% MHCII+ in SIRPa+CD115+
I
Blood
Lin-SIRPa+CD115+
Flt3l+/+
Flt3l-/-
% of live
Abs.no/ ul blood
J
Blood
Lin-SIRPa+CD115+
Ccr2+/+
Ccr2-/-
% of live
Abs. no./ul of blood

Figure S2 (Related to Figure 2): Transcriptional control of MHCII expression on R1 and R2 monocytes and R3 pre-DCs.

A,B) Expression of FcgR and CD209 in R1,R2 and R3. Average values of 3 replicates of microarray data of FcgR and CD209 in R1 and R2 monocytes and R3 pre-cDCs (A). Expression of FcgRII/III and CD209a versus respective isotype controls as assessed by FACS analysis (B).

C) -DCt values in Clusters1-5 and populations R1-3, of *Ciita, H2-Aa* and *H2-Ab* as derived from single cell multiplex qPCR analysis.

D) Microarray analysis of MHCII-related genes in R1, R2 and R3. Average values of triplicate samples in R1, R2 and R3 are shown for MHCII-related genes.

E) MHCII expression in blood $Lin^{-}CD115^{+}Ly6C^{+}$ cells at steady state.

F) MHCII expression in $Ly6C^{+}CD115^{+}$ cells in the BM and blood at steady state.

Intracellular staining of MHCII or isotype control in the BM and staining of surface MHCII in blood of WT $Lin^{-}SIRPa^{+}CD115^{+}$ cells. Histograms show the percentage of $Flt3^{+}$ cells within the $Lin^{-}SIRPa^{+}CD115^{+}Ly6C^{+}$ $MHCII^{+}$ and $MHCII^{-}$ cells.

G) Intracellular expression of MHCII in BM R1, R2 and R3 in WT, $pI^{-/-}$, $pIII$+$pIV^{-/-}$, $pIV^{-/-}$ BM at steady state. Percentages of parent indicated above each gate ± SEM.

H) MHCII expression in total BM and blood $Lin^{-}CD115^{+}SIRP\alpha^{+}$ cells in $pI^{-/-}$, $pIII^{+}pIV^{-/-}$, $pIV^{-/-}$ and $Ciita^{-/-}$ mice at steady state.

I) $MHCII^{+}$ fraction of R2 cells is independent of Flt3L. FACS analysis and quantification in percentage of live cells and absolute number of the $MHCII^{+}$ cells within R2 in the blood of $Flt3l^{-/-}$ and $Flt3^{+/+}$ mice.

J) $MHCII^{+}$ fraction of R2 cells is dependent on CCR2. FACS analysis and quantification in percentage of live cells and of $Ly6C^{+}$ monocytes of the $MHCII^{+}$ cells within R2 in $Ccr2^{-/-}$ and $Ccr2^{+/+}$ mice. Mean ± SEM, *p<0.05, **<0.005, ***p<0.0005, ****p<0.00005, Student's t-test.

FIGURE S3

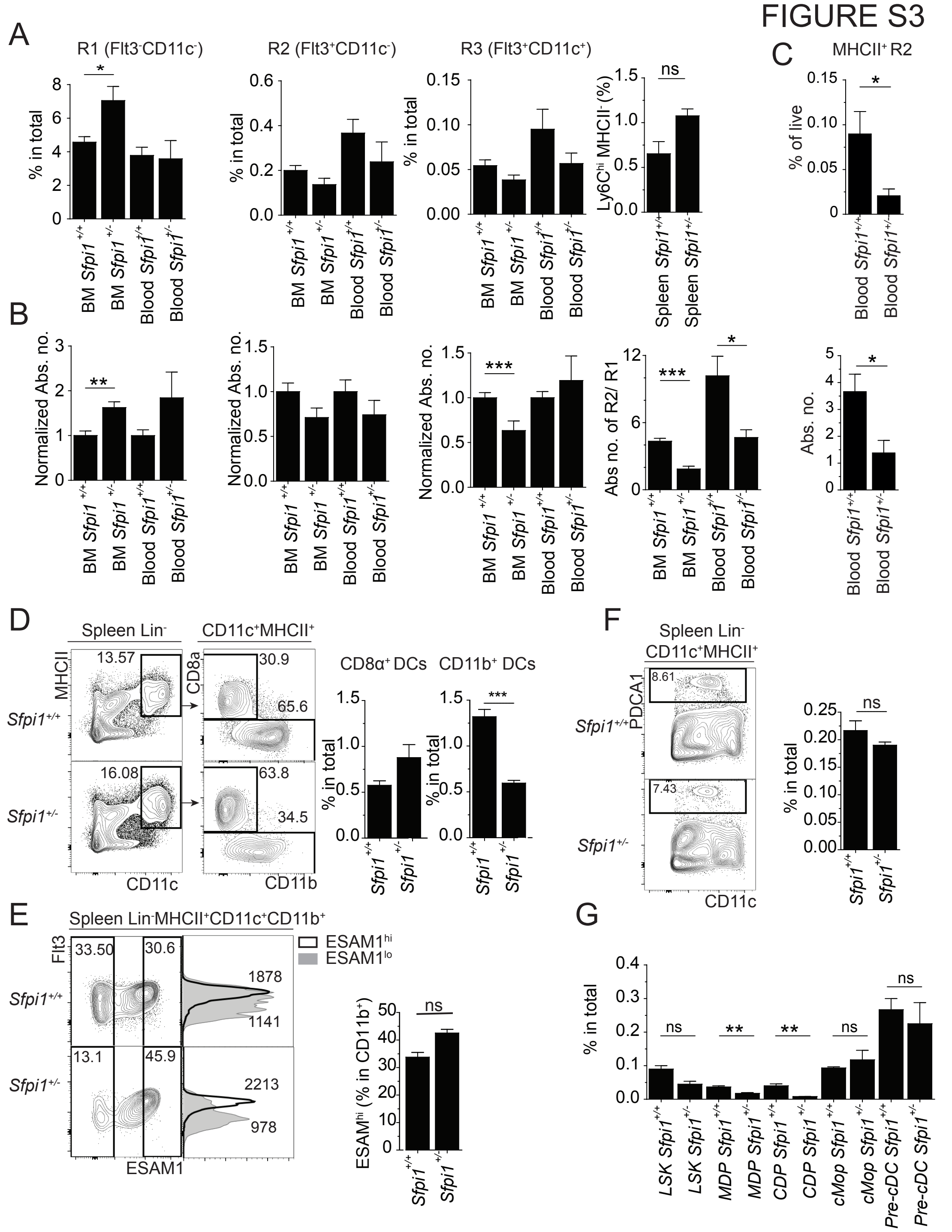

A
R1 (Flt3-CD11c-)
R2 (Flt3+CD11c-)
R3 (Flt3+CD11c+)
% in total
Ly6Chi MHCII- (%)
ns
BM Sfpi1+/+
BM Sfpi1+/-
Blood Sfpi1+/+
Blood Sfpi1+/-
Spleen Sfpi1+/+
Spleen Sfpi1+/-
C
MHCII+ R2
% of live
Abs. no.
B
Normalized Abs. no.
Abs no. of R2/ R1
D
Spleen Lin-
CD11c+MHCII+
MHCII
CD8a
CD11c
CD11b
13.57
16.08
30.9
65.6
63.8
34.5
CD8α+ DCs
CD11b+ DCs
Sfpi1+/+
Sfpi1+/-
E
Spleen Lin-MHCII+CD11c+CD11b+
Flt3
ESAM1
ESAM1hi
ESAM1lo
33.50
30.6
13.1
45.9
1878
1141
2213
978
ESAMhi (% in CD11b+)
F
Spleen Lin- CD11c+MHCII+
PDCA1
CD11c
8.61
7.43
G
LSK Sfpi1+/+
LSK Sfpi1+/-
MDP Sfpi1+/+
MDP Sfpi1+/-
CDP Sfpi1+/+
CDP Sfpi1+/-
cMop Sfpi1+/+
cMop Sfpi1+/-
Pre-cDC Sfpi1+/+
Pre-cDC Sfpi1+/-

Figure S3 (Related to Figure 3): PU1 levels influence the development of R2 monocytes and R3 pre-DCs.

A,B) Cellularity of *Sfpi1*$^{+/+}$ and *Sfpi1*$^{+/-}$ BM, blood and spleen. Quantification of percentage in total cells (A) and normalized absolute number (normalized to *Sfpi1*$^{+/+}$ controls) (B) of BM and blood R1 and R2 monocytes and R3 pre-DCs from *Sfpi1*$^{+/+}$ and *Sfpi1*$^{+/-}$ mice. Percentage of total Ly6C^{hi}MHCII^{-} cells in total splenocytes and the fold change of absolute number of R2 over R1 monocytes in the BM and blood of *Sfpi1*$^{+/+}$ and *Sfpi1*$^{+/-}$ mice.

C) MHCII^{+} fraction of R2 is PU.1 – dependent. Percentage of live and absolute number of the MHCII^{+} cells within R2 in the blood of *Sfpi1*$^{+/+}$ versus *Sfpi1*$^{+/-}$ mice.

D-F) Cellularity of *Sfpi1*$^{+/+}$ and *Sfpi1*$^{+/-}$ spleen total DCs, CD11b^{+} DCs and pDCs.

FACS analysis of the Lin^{-}(CD19^{-}, Ly6G^{-}, CD3e^{-}, NK1.1^{-}, Ter119^{-}, CD45RA^{-}, ckit^{-}) CD11c^{+}MHCII^{+} CD8α^{+} and CD11b^{+} DCs (D) ESAM^{hi-} and ESAM^{lo-} CD11b^{+} DCs (E) and PDCA1^{+} cells (F) in the spleen of *Sfpi1*$^{+/+}$ and *Sfpi1*$^{+/-}$ mice. Histograms in D show the levels of Flt3 in the ESAM^{hi} (solid black line) and the ESAM^{lo} DCs (shaded grey) with mean fluorescence intensity (MFI) indicated adjacent to the respective histogram.

G) Percentage in total BM cells of LSK, MDP, CDP, cMop and Pre-DC populations from *Sfpi1*$^{+/+}$ and *Sfpi1*$^{+/-}$ mice.

(Data shown is mean ± SEM, *$p<0.05$, ***$p<0.0005$, ns= non-significant; Student's t-test)

FIGURE S4

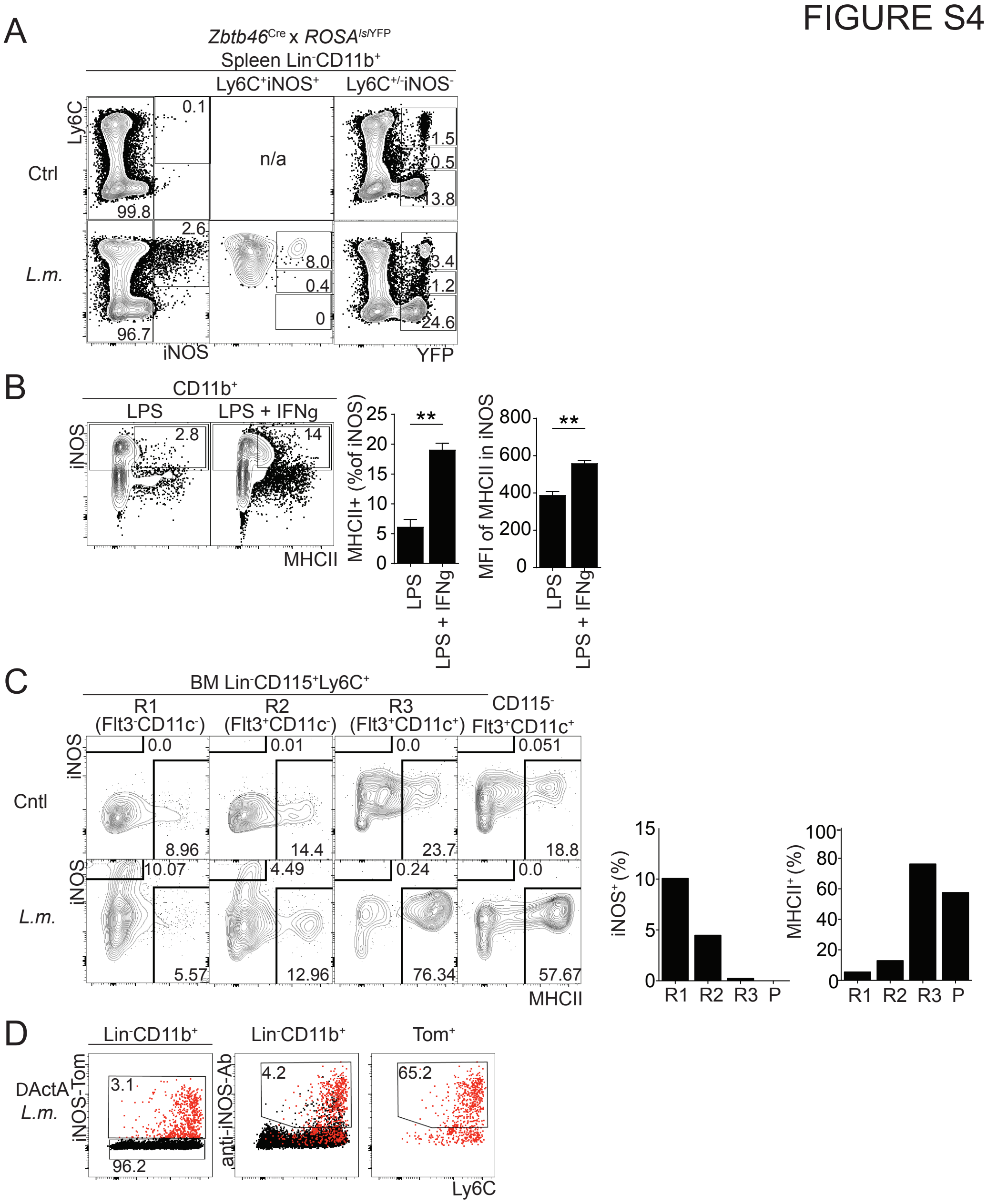
A
Zbtb46Cre x ROSAlsl/YFP
Spleen Lin-CD11b+
Ly6C+iNOS+
Ly6C+/-iNOS-
Ctrl
L.m.
Ly6C
iNOS
YFP
n/a
0.1
99.8
1.5
0.5
3.8
2.6
96.7
8.0
0.4
0
3.4
1.2
24.6
B
CD11b+
LPS
LPS + IFNg
iNOS
MHCII
2.8
14
MHCII+ (%of iNOS)
MFI of MHCII in iNOS
**
C
BM Lin-CD115+Ly6C+
R1 (Flt3-CD11c-)
R2 (Flt3+CD11c-)
R3 (Flt3+CD11c+)
CD115- Flt3+CD11c+
Cntl
L.m.
iNOS
MHCII
0.0
0.01
0.0
0.051
8.96
14.4
23.7
18.8
10.07
4.49
0.24
0.0
5.57
12.96
76.34
57.67
iNOS+ (%)
MHCII+ (%)
R1
R2
R3
P
D
Lin-CD11b+
Lin-CD11b+
Tom+
DActA L.m.
iNOS-Tom
anti-iNOS-Ab
Ly6C
3.1
96.2
4.2
65.2

Figure S4 (Related to Figure 4): iNOS production is restricted to activated R1 monocytes.

A) DC lineage tracing of $iNOS^{+}$ cells. FACS analysis of YFP expression in $Lin^{-}CD11b^{+}$ $iNOS^{+}$ and $iNOS^{-}$ cells in *L.m.*-infected *Zbtb46*^{Cre} *x ROSA* $^{lsl\ YFP}$ mice (day2).

B) MHCII expression in total WT BM after overnight culture with LPS, with or without IFNγ.

C) *In vitro* overnight stimulation with *L.m.* or no stimulation, of R1 and R2 monocytes and pre- DCs R3 and P.

D) Anti-iNOS antibody staining of iNOS-$Tomato^{+}$ (red cells) and total $Lin^{-}CD11b^{+}$ cells in ΔActA-*L.m*. infected *Nos2*^{TomatoCRE}*x ROSA*^{lslTomato} mice.

FIGURE S5

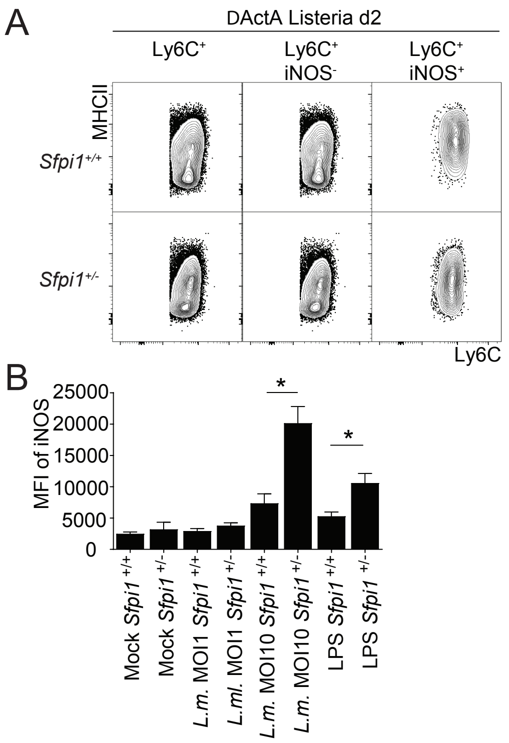

Figure S5 (Related to Figure 5): PU.1 inhibits the generation of iNOS$^+$ macrophages upon microbial stimulation.

A) MHCII expression of Ly6C$^+$, Ly6C$^+$iNOS$^-$ and Ly6C$^+$iNOS$^+$splenocytes from ΔActA-*L.m.* infected *Sfpi1*$^{+/+}$ versus *Sfpi1*$^{+/-}$ mice.

B) Mean fluorescence intensity (MFI) of anti-iNOS staining on BMDMs from *Sfpi1*$^{+/+}$ and *Sfpi1*$^{+/-}$ mice cultured overnight with plain media, *L.m.*(MOI of 1 or 10 CFU) or LPS (1ug/ml).

FIGURE S6

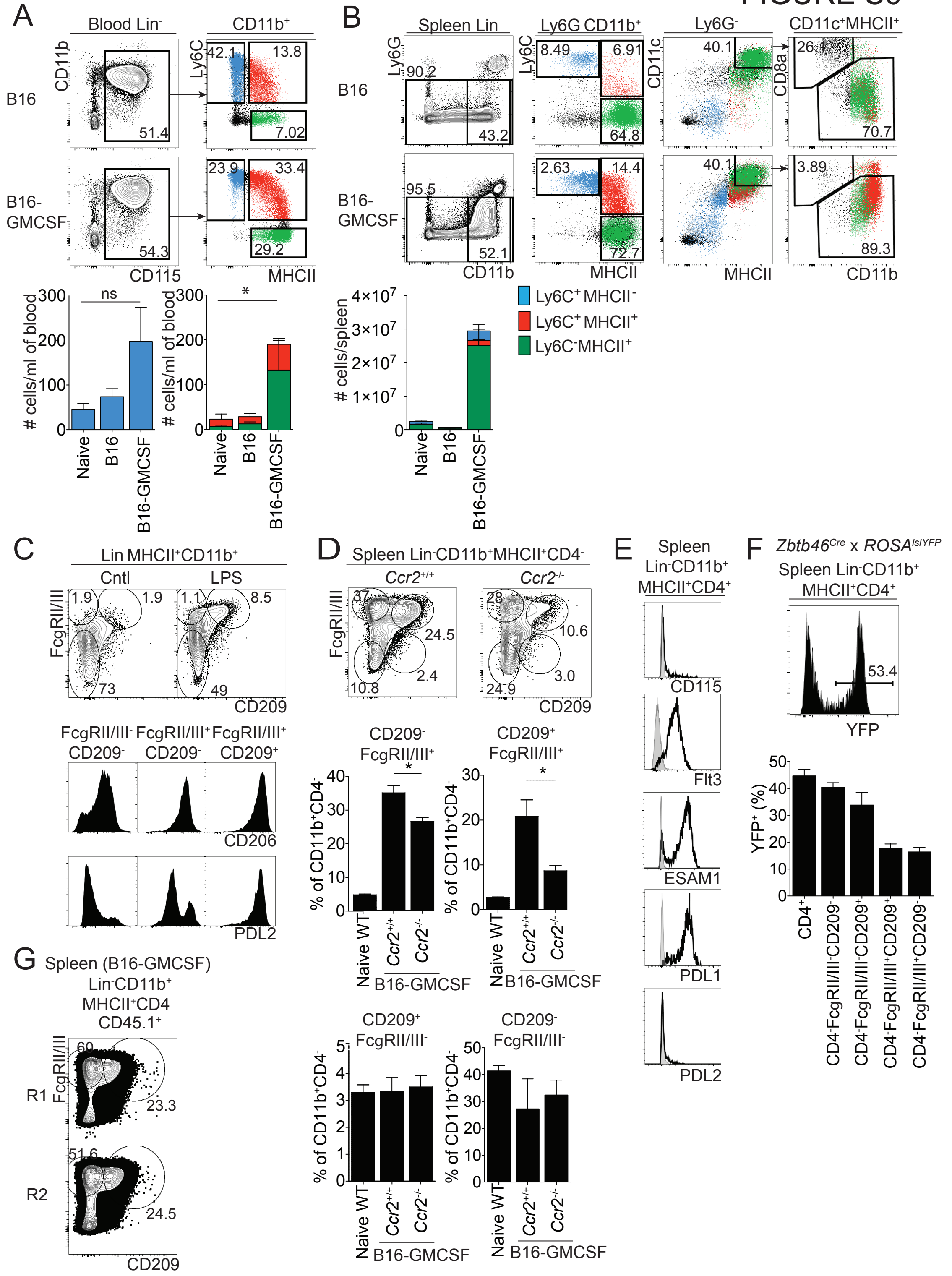
A
Blood Lin-
CD11b+
B16
B16-GMCSF
CD11b
CD115
Ly6C
MHCII
cells/ml of blood
Naive
B16
B16-GMCSF
B
Spleen Lin-
Ly6G-CD11b+
Ly6G-
CD11c+MHCII+
Ly6G
CD11b
Ly6C
MHCII
CD11c
CD8a
cells/spleen
Ly6C+ MHCII-
Ly6C+ MHCII+
Ly6C- MHCII+
C
Lin-MHCII+CD11b+
Cntl
LPS
FcgRII/III
CD209
FcgRII/III- CD209-
FcgRII/III+ CD209-
FcgRII/III+ CD209+
CD206
PDL2
D
Spleen Lin-CD11b+MHCII+CD4-
Ccr2+/+
Ccr2-/-
CD209- FcgRII/III+
CD209+ FcgRII/III+
CD209+ FcgRII/III-
CD209- FcgRII/III-
% of CD11b+CD4-
Naive WT
B16-GMCSF
E
Spleen Lin-CD11b+ MHCII+CD4+
CD115
Flt3
ESAM1
PDL1
PDL2
F
Zbtb46Cre x ROSAlslYFP
Spleen Lin-CD11b+ MHCII+CD4+
YFP
YFP+ (%)
CD4+
CD4-FcgRII/III-CD209-
CD4-FcgRII/III-CD209+
CD4-FcgRII/III+CD209+
CD4-FcgRII/III+CD209-
G
Spleen (B16-GMCSF)
Lin-CD11b+ MHCII+CD4- CD45.1+
R1
R2
FcgRII/III
CD209

Figure S6 (related to Figure 6): PU.1^{high}Flt3^{+}MHCII^{+} R2 monocytes differentiate into PD-L2^{+}CD209^{+} moDCs upon exposure to GM-CSF

A,B) Phenotype of blood cells and splenocytes from mice bearing B16 or B16-GMCSF tumours. FACS analysis of the Ly6C^{+}MHCII^{-} (blue), Ly6C^{+}MHCII^{+} (red) Ly6C^{-}MHCII^{+} (green) cells within the Lin^{-}(Ly6G^{-}, CD19^{-}, CD3ε^{-}, NK1.1^{-}, Ter119^{-}, CD45RA^{-}, cKit^{-}) CD115^{+} cells in the blood (A) and the Lin^{-} Ly6G^{-}CD11b^{+} cells in spleen (B) of B16 or B16-GMCSF tumor-bearing mice. FACS staining for CD11c and MHCII in Lin^{-}Ly6G^{-} cells of the spleen showing the overlap of Ly6C^{+}MHCII^{-} (blue), Ly6C^{+}MHCII^{+} (red) and Ly6C^{+}MHCII^{+} (green) cells within the DC gating (B). n= 3-6 mice per group (A,B) (Mean ± SEM;* p<0.05, ns= non-significant; Student's unpaired t-test.)

C) Phenotype of lymph nodes after LPS treatment. FACS analysis for FcɣRII/III and CD209a expression in the Lin^{-}MHCII^{-}CD11b^{+} cells of mice treated either with PBS or LPS. CD206 and PDL2 phenotype of the FcɣRII/III^{+}CD209^{+}, FcɣRII/III^{+}CD209^{-} and the FcɣRII/III^{-}CD209^{-} cells is shown.

D) Role of CCR2 in GM-CSF-dependent CD11b^{+}MHCII^{+} splenocytes.

FACS analysis of FcgRII/III^{+}CD209^{-}, FcgRII/III^{+}CD209^{+}, FcgRII/III^{-}CD209^{+} and FcgRII/III^{-}CD209^{-} in WT and *Ccr2*$^{-/-}$ mice bearing B16-GMCSF tumors. Percentage in the Lin^{-}CD11b^{+}MHCII^{+}CD4^{-} splenocytes of each subset is shown. Mean ± SEM, * p<0.05, Student's unpaired t-test.

E) Phenotype of naïve CD4^{+} DCs in the spleen. FACS staining of Lin^{-}CD11b^{+}MHCII^{+}CD4^{+} cells for CD115, Flt3, ESAM1, PDL1 (CD274) and PDL2 (CD273) in naïve mice.

F) *Zbtb46*^{Cre} *x ROSA* ^{lslYFP} fate mapping. FACS analysis of YFP expression on Lin^{-} CD11b^{+}MHCII^{+}CD4^{+} cells from naïve *Zbtb46*^{Cre} *x ROSA* ^{lslYFP}mice. Percentage of YFP^{+} cells within Lin^{-}CD11b^{+}MHCII^{+} subsets from B16-GMCSF-tumour-engrafted *Zbtb46*^{Cre} *x ROSA* ^{lslYFP}mice.

G) Endogenous expression of FcɣRII/III and CD209a in Lin^{-}CD11b^{+}MHCII^{+}CD4^{-} cells in the tumour-bearing WT (CD45.1) mice adoptively transferred with R1 (CD45.2) or R2 (CD45.2).

FIGURE S7

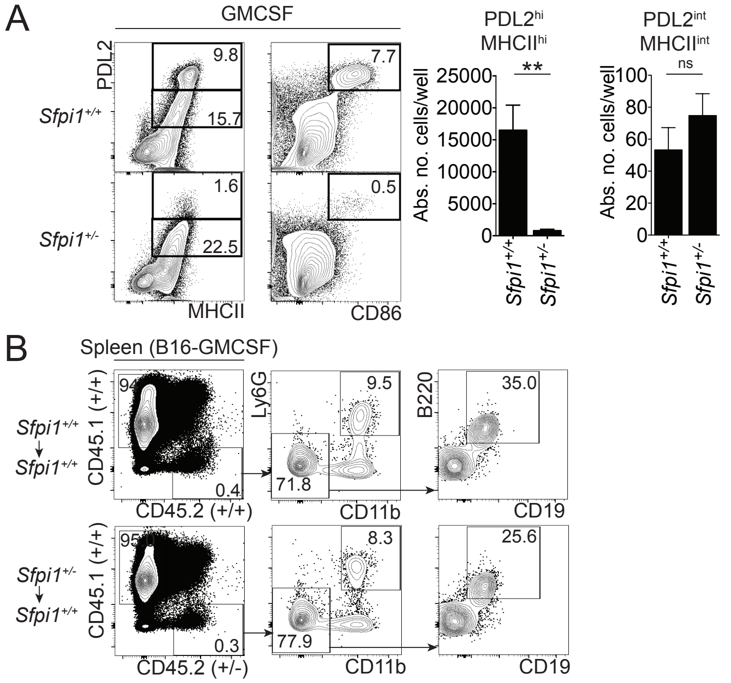

Figure S7 (Related to Figure 7): PU1 influences the number of the PDL2^{+}MHCII^{+} cells generated *in vitro* in response to GMCSF.

A) GM-CSF culture of total BM from *Sfpi1*$^{+/+}$ and *Sfpi1*$^{+/-}$ mice.

FACS analysis for PDL2 (CD273), MHCII and CD86 from DAPI^{-} progeny obtained after 5 days of GMCSF culture. Absolute number of PDL2^{+}MHCII^{hi} and PDL2^{int}MHCII^{int} cells is quantified in the graphs shown. n=4 independent cultures.

B) Phenotype of adoptively transferred *Sfpi1*$^{+/+}$ or *Sfpi1*$^{+/-}$ whole BM into B16-GMCSF treated CD45.1^{+} congenic recipients. FACS analysis of CD45.1^{+} (recipient) and CD45.2^{+} (donor) granulocytes (Ly6G^{+}CD11b^{+}) and B cells (Ly6G^{-}CD11b^{-}CD19^{+} B220^{+}) in B16-GMCSF tumor engrafted CD45.1^{+} mice.

C) Graphical summary of proposed model.

Ly6C^{+} monocytes consist of 2 subsets- PU1^{lo} R1 and PU1^{hi} R2, that are independently capable of developing into different inflammatory progeny. R1 gives rise to FcɣRII/III^{+}CD209a^{-} cells in response

to GM-CSF or $iNOS^+$ cells in response to microbial stimulation. R2 produces $FcgRII/III^+CD209^+$ mo-DCs in response to GM-CSF. These different roles are dictated by increased or decreased levels of PU.1 expression.

Supplemental Experimental Procedures

Mice: C57Bl/6 mice were bought from Charles River Laboratories, UK. *Ccr2*$^{-/-}$ (Boring et al., 1997), *Nr4a1*$^{-/-}$ (Lee et al., 1995), *Sfpi1*$^{+/-}$ (McKercher et al., 1996), CD45.1^{+} (JAX:002014) mice were housed within the clean unit of the animal housing facility at King's College London. *Zbtb46-GFP* mice (Satpathy et al., 2012)were housed at the animal facility at Queen Mary, University of London. *pIII +pIV*$^{-/-}$: (LeibundGut-Landmann et al., 2004), *pIV*$^{-/-}$ (Waldburger et al., 2001), *pI*$^{-/-}$ (Dubrot et al., 2014) and *Ciita*$^{-/-}$ (JAX: 003239) micewere housed at Geneva University, Switzerland. All mice maintained under specific pathogen-free conditions in accordance with the UK Animals (Scientific Procedures) Act,1986. *Zbtb46*^{Cre} *x ROSA*^{loxSTOPloxYFP} (Jakob Loshk, 2016)
, *NOS2*$^{Tomato\text{-}Cre}$*xROSA*^{loxSTOPloxtdTomato} (Bechade et al., 2014) bone marrow was injected into lethally irradiated recipients and used in all experiments as indicated.

Reagents: Complete medium used for cell culture was RPMI (Life technologies) with Glutamax, and 10% FBS (Life technologies) and 50uM beta-mercaptoethanol (Sigma). FACS buffer used was made of PBS (Life Technologies) with 1% bovine serum albumin (Apollo Scientific) and 2mM EDTA.

Bone marrow Chimeras: 8-10 week old C57Bl/6 mice were hematopoietically-lethal irradiated with 11Gy; bone marrow from *Zbtb46-Cre-loxSTOPloxYFP* (Loshko *et al*, 2016) , *Zbtb46-iDTR* mice (Meredith et al., 2012)or *NOS2-*$^{Tomato\text{-}Cre}$*xROSA*^{loxSTOPloxtdTomato} (Bechade et al., 2014) was injected intravenously at a dose of 5 x 10^{6} cells in100ul into these irradiated recipients. To allow full reconstitution, the mice were used at 8-16 weeks after transplantation. *Ccr2*$^{-/-}$ (CD45.2+) and WT (CD45.1+) mixed bone marrow chimeras were

produced in hematopoietically-lethal irradiated (11Gy) CD45.1+ recipients that received CD45.1^{+} WT and *Ccr2*$^{-/-}$ (CD45.2^{+}) bone marrow in equal parts. CD45.1+ recipients that received 100%CD45.2+ C57Bl/6 bone morrow were used as a control for the complete replacement of recipient bone marrow with donor-derived bone marrow.

FACS Reagents: Fluorochrome or biotin- conjugated antibodies were used to stain single cell suspensions for flow cytometry. These included monoclonal antibodies specific to mouse CD19 (Clone MB19-1), Ly6G (Clone: 1A8),CD3ε (Clone 145-2C11), Ter119 (Clone Ter119), NK1.1 (Clone PK136), CD45RA (Clone: HI100), ckit (Clone:2B8), MHCII I-A/I-E (Clone: M5/114. 15.2), Ly6C (clone: HK1.4), CD11b (clone:M1/70), CD115 (clone:AFS98), CD16/32 (clone: 93), CD209 (clone: LW206), CD135 (clone A2F10), CD172a (clone: P84), CD11c (clone: N418), CD273 (clone: TY25), CD274 (clone:10F.9G2) that were bought from eBioscience (Hatfield, UK) and Biolegend (London, UK). For staining intracellular levels of iNOS, cells were fixed and permeabilized using BD Cytofix/Cytoperm Fixation kit as per manufacturer's instructions and stained with anti-iNOSA488 or -iNOS-PE (clone: CXNFT) purchased from eBioscience. For staining PU.1, cells were fixed and permeabilized using FoxP3/ Transcription factor staining buffer set (eBioscience) according to manufacturer's instructions. Anti-PU.1 rabbit monoclonal antibody (clone: 9G7) and the corresponding isotype were purchased from Cell Signaling Technology (New England Biolabs (UK) Ltd).

Cell sorting by flow cytometry

For sorting, bone marrow or spleen cells were resuspended to an approximate concentration of 7000cells/ul. They were then sorted on a BD FACS Aria (special order machine) fitted with 405nm, 488nm, 561nm, 633nm lasers and sorted through 100um nozzle with 4-way purity. Purity checks were run on samples used for microarrays and were used when purity

was found to be >95%. Both instruments were housed at the Biomedical Research Centre Flow Core Facility (Guy's and St Thomas' NHS Foundation Trust and King's College London). Flow cytometry analysis was done using FlowJo software (TreeStar).

Giemsa Staining: Cytospins of FACS sorted R1, R2, R3 and P were fixed with methanol for 5 mins, stained with 1:20 Giemsa stain in deionized water for 45 mins and then washed and air dried. Slides were imaged on Motic AE2000 with 40x magnification. Images were modified for brightness with ImageJ (NIH).

***In vitro L.m.* infections**: *In vitro* cultures of primary sorted cells with *Listeria monocytogenes* (*L.m.)* was done overnight at an MOI of 0.01 or 0.1 as indicated in complete RPMI1640 medium supplemented with MCSF (20ng/ml) (Peprotech), GMCSF(3ng/ml) (Peprotech) and human Flt3L(100ng/ml) (CellDex). BMDMs were derived from *Sfpi1*$^{+/-}$ or *Sfpi1*$^{+/+}$ BM cultured for 8-10 days in DMEM medium (Life technologies) supplemented with 10% FBS and 10% MCSF containing L-929 cell culture supernatant. These were re-plated as 0.45 x 10^6 cells/well in a 24-well non-tissue culture treated plate to be stimulated with LPS (1ug/ml) or *L.m.* at MOI 1 or 10 for 16 hours. Cells were collected, stained with fluorochrome conjugated antibodies and analysed by FACS.

B16-GMCSF tumor experiments: Melanoma cell lines B16 and B16 expressing GMCSF (B16-GMCSF)(Dranoff et al., 1993) were maintained in RPMI1640 medium supplemented with Glutamax (Life technologies), 10% fetal bovine serum (Life technologies), 1% penicillin-streptomycin (Life Techonologies) and 50uM beta-mercaptoethanol (Sigma) and used from between passages 4 and 10. Cells were checked for viability with Trypan Blue and 1.5-3 x 10^5 live cells were injected subcutaneously in sterile RPMI 1640 medium alone.

Infection. *Listeria monocytogenes* (*L.m.*) or the ΔActA mutant of the same (ΔActA *Listeria)* were grown and sub-cultured in brain heart infusion broth at 37^0C until an OD_{600} value of 0.12-0.15 was obtained to use bacteria in their exponential growth phase. 4 – 5 x 10^3 wild type CFU (Listeria) or 10^6 ΔActA mutant CFU of *Listeria monocytogenes* were injected intravenously in sterile PBS.

Microarray processing and analysis:

Affymetrix CEL files were converted into gct files using the ExpressionFileCreator Module within Gene Pattern Software (Broad Institute) (Reich et al., 2006). The RMA algorithm withquantile normalization and background correction was used. No thresholds or filters were applied for assessing the relative expression of all genes assayed on the microarray. Heat maps were generated with this data on Gene-E software.

To create the Volcano plots, Mutiplot Preprocess Module within the Gene Pattern Software (Broad Institute) was used to derive fold change and p-values from the expression dataset of the aforementioned microarrays to be used in the MultiplotVisualizer Module. This latter module was used to highlight the genes more highly expressed in R2 or R3 above a threshold of p-value set at 0.05 and fold change of 1.2. These selected genes were then overlaid on comparisons done between R1 and R2, and R1 and R3 to obtain the plots shown in Fig.1F.

PCA analysis and hierarchical clustering: Microarray data of R1, R2 and R3 were compared with ST1.0 array data available on ImmGEN for Pre-DCs (GSE68590)) (Tussiwand et al., 2015) (and CDP (GSE 15907) (www.immgen.org) on Qlucore Omics Explorer (Sweden) and plotted as 2D plots on Prism (Graphpad). Hierarchical clustering of data sets was performed using Gene-E software (Broad Institute).

qPCR Primers: Cells were sorted as described and centrifuged. Supernatant was removed and cells were resuspended in RLT buffer from the RNeasy kit (Qiagen). mRNA was extracted using the columns as per manufacturer's instructions. mRNA was resuspended in RNase-free water and the concentration and quality measured by nanodrop (Thermo Scientific). Equal amounts of mRNA (between 0.1ng – 5ug) from each sample were taken to produce cDNA using the manufacturer's First Strand cDNA synthesis protocol with the RevertAid™ H minus Reverse Transcriptase (Thermo Scientific). Random Primers (Oligo dT primers) were mixed with template RNA and incubated at 65°C for 5 mins and Ribolock (Thermo Scientific), dNTP mix, Reaction buffer (5x) and mMulV reverse transcriptase enzyme were added and incubated at RT for 10 mins, 42°C for 1hr and at 70°C for 5 mins to inactivate the enzyme. A 1 in 10 dilution of this cDNA was used to perform qPCR with Sensimix™SYBR® (Bioline) as per manufacturer's instructions.

Multidimensional reduction analysis: Automated t-distributed stochastic non-linear embedding (t-SNE) algorithm was used to visually (viSNE) analyse (Amir el et al., 2013) bone marrow monocytes acquired by FACS for 7 fluorochromes. The online (web-based) software implementation of viSNE (Cytobank) (Kotecha et al., 2010) was used to analyse the presence of different populations within the Ly6C^{hi}CD115^{+} BM monocytes. No *a priori* gating was used and an unbiased automated analysis was conducted. The resulting viSNE maps were overlaid with each monocyte population, R1, R2 and pre-DC R3 that were gated separately by conventional FACS analysis. The colour scheme for all four viSNE maps was adjusted to represent the Flt3 expression – blue colour denoting lower levels and red – higher.

Single cell qPCR: Single cells were FACS sorted from bone marrow into 9ul of Cell Direct pre-Amp master mix in 96-well qPCR plates. Complementary DNA (cDNA) synthesis and specific target amplification of 45 genes (including 3 housekeeping genes- Hprt, ActB, Gapdh) was performed using CellsDirect One Step qRT-PCR kit (Invitrogen) with 48 Taqman assays (Life technologies) at 0.2x. Reverse transcription was performed within the same plates using the following cycle: 40^0C for15 mins, 50^0C for 15 mins, 60^0C for 15 mins. Enzyme inactivation was done at 95^0C for 2mins followed by 22x (95^0C for 15s, 60^0C for 4mins). cDNA was then diluted 1 in 5 in low EDTA TE buffer. Samples were stored at -20^0C until used in the BioMark. 81 cells of R2, and 44 cells of R1 and R3 each were compared along with control well including 10 cells and no cell controls and dilutions of cDNA from 10^5 cells to 1 cells to check for primer viability. 5ul of diluted cDNA + Taqman mastermix + Sample loading reagent and 5 ul of each Taqman assay + Assay loading reagent were loaded into their respective wells on 4 M48.M48 Dynamic Arrays. Samples and Assays were then loaded into the reaction chambers of the Dynamic Array using the IFC ControllerMX (Fluidigm), and then transferred to the BioMark HD for qPCR (95˚C for 10 min; 40 cycles of 95˚C for 15 s and 60 ˚C for 60 s). Data obtained from the ‘Real time PCR analysis’ software (Fluidigm) was analysed using Gene-E software.

The following Taqman probes were tested on all samples.

	Primers
Adamts3	Mm00625880_m1
Cd209a	Mm00460067_m1
Cd209c	Mm00652419_m1
Cd209d	Mm00459972_m1
Cd209e	Mm00459980_m1
Cd74	Mm01262763_m1
Ciita	Mm00482914_m1
Clec10a	Mm00546124_m1
Clec4a2	Mm00488795_m1
Clec4g	Mm01212425_m1
Clec5a	Mm01131766_m1
Clec7a	Mm00490960_m1
Clec9a	Mm00554956_m1
Csf1r	Mm01266652_m1
Csf2ra	Mm00438331_g1
Csf2rb	Mm00655745_m1
Csf3r	Mm00432735_m1
Ctsb	Mm01310506_m1
CtsG	Mm00456011_m1
Cybb	Mm01287743_m1
Fcgr2b	Mm00438879_m1
Fcgr3	Mm00438882_m1
Fcgr4	Mm00519988_m1
Flt3	Mm00439016_m1
H2-Aa	Mm00439211_m1
H2-Ab1	Mm01271199_m1
H2-dma	Mm04337015_m1
H2-dMb2	Mm00783707_s1
Id2	Mm00711781_m1
Irf8	Mm00492567_m1
Klrb1f	Mm04211785_m1
Klrd1	Mm00495182_m1
Kmo	Mm01321343_m1
Mgl2	Mm01250813_m1
Mpo	Mm01298424_m1
Mrc1	Mm00485155_m1
Ms4a3	Mm00460072_m1
Nfil3	Mm00600292_s1
Sfpi1	Mm00488140_m1
Spib	Mm03048233_m1
Tcfec	Mm01161234_m1
Zbtb46	Mm00511327_m1

These genes were selected for their discriminating capacity based on the PCA analysis between sorted populations of R1, R2 and R3 along with genes with previously described expression in monocytes and DCs.

Statistical Analysis. Data was analysed for statistical significance by unpaired Student's t-test. Differences were considered significant for $p<0.05$. *$p<0.05$, **$p<0.005$, ***$p<0.0005$, ****$p<0.00005$.

Supplementary References:

Amir el, A.D., Davis, K.L., Tadmor, M.D., Simonds, E.F., Levine, J.H., Bendall, S.C., Shenfeld, D.K., Krishnaswamy, S., Nolan, G.P., and Pe'er, D. (2013). viSNE enables visualization of high dimensional single-cell data and reveals phenotypic heterogeneity of leukemia. Nat Biotechnol *31*, 545-552.

Bechade, C., Colasse, S., Diana, M.A., Rouault, M., and Bessis, A. (2014). NOS2 expression is restricted to neurons in the healthy brain but is triggered in microglia upon inflammation. Glia *62*, 956-963.

Boring, L., Gosling, J., Chensue, S.W., Kunkel, S.L., Farese, R.V., Jr., Broxmeyer, H.E., and Charo, I.F. (1997). Impaired monocyte migration and reduced type 1 (Th1) cytokine responses in C-C chemokine receptor 2 knockout mice. J Clin Invest *100*, 2552-2561.

Dranoff, G., Jaffee, E., Lazenby, A., Golumbek, P., Levitsky, H., Brose, K., Jackson, V., Hamada, H., Pardoll, D., and Mulligan, R.C. (1993). Vaccination with irradiated tumor cells engineered to secrete murine granulocyte-macrophage colony-stimulating factor stimulates potent, specific, and long-lasting anti-tumor immunity. Proc Natl Acad Sci U S A *90*, 3539-3543.

Dubrot, J., Duraes, F.V., Potin, L., Capotosti, F., Brighouse, D., Suter, T., LeibundGut-Landmann, S., Garbi, N., Reith, W., Swartz, M.A., *et al.* (2014). Lymph node stromal cells acquire peptide-MHCII complexes from dendritic cells and induce antigen-specific CD4(+) T cell tolerance. J Exp Med *211*, 1153-1166.

Kotecha, N., Krutzik, P.O., and Irish, J.M. (2010). Web-based analysis and publication of flow cytometry experiments. Curr Protoc Cytom *Chapter 10*, Unit10 17.

Lee, S.L., Wesselschmidt, R.L., Linette, G.P., Kanagawa, O., Russell, J.H., and Milbrandt, J. (1995). Unimpaired thymic and peripheral T cell death in mice lacking the nuclear receptor NGFI-B (Nur77). Science *269*, 532-535.

LeibundGut-Landmann, S., Waldburger, J.M., Reis e Sousa, C., Acha-Orbea, H., and Reith, W. (2004). MHC class II expression is differentially regulated in plasmacytoid and conventional dendritic cells. Nat Immunol *5*, 899-908.

McKercher, S.R., Torbett, B.E., Anderson, K.L., Henkel, G.W., Vestal, D.J., Baribault, H., Klemsz, M., Feeney, A.J., Wu, G.E., Paige, C.J., *et al.* (1996). Targeted disruption of the PU.1 gene results in multiple hematopoietic abnormalities. EMBO J *15*, 5647-5658.

Meredith, M.M., Liu, K., Darrasse-Jeze, G., Kamphorst, A.O., Schreiber, H.A., Guermonprez, P., Idoyaga, J., Cheong, C., Yao, K.H., Niec, R.E., *et al.* (2012). Expression of the zinc finger transcription factor zDC (Zbtb46, Btbd4) defines the classical dendritic cell lineage. J Exp Med *209*, 1153-1165.

Reich, M., Liefeld, T., Gould, J., Lerner, J., Tamayo, P., and Mesirov, J.P. (2006). GenePattern 2.0. Nature genetics *38*, 500-501.

Satpathy, A.T., Wumesh, K.C., Albring, J.C., Edelson, B.T., Kretzer, N.M., Bhattacharya, D., Murphy, T.L., and Murphy, K.M. (2012). Zbtb46 expression distinguishes classical dendritic cells and their committed progenitors from other immune lineages. Journal of Experimental Medicine *209*, 1135-1152.

Tussiwand, R., Everts, B., Grajales-Reyes, G.E., Kretzer, N.M., Iwata, A., Bagaitkar, J., Wu, X., Wong, R., Anderson, D.A., Murphy, T.L., *et al.* (2015). Klf4 expression in conventional dendritic cells is required for T helper 2 cell responses. Immunity *42*, 916-928.

Waldburger, J.M., Suter, T., Fontana, A., Acha-Orbea, H., and Reith, W. (2001). Selective abrogation of major histocompatibility complex class II expression on extrahematopoietic cells in mice lacking promoter IV of the class II transactivator gene. J Exp Med *194*, 393-406.
