# Supplementary material for: The Heterogeneity of Ly6Chi Monocytes Controls Their Differentiation into iNOS+ Macrophages or Monocyte-Derived Dendritic Cells
Source: Immunity. 2016 Dec 20;45(6):1205–18. doi: 10.1016/j.immuni.2016.12.001 (PMC5196026; doi:10.1016/j.immuni.2016.12.001)
Supplement: Document S2. Article plus Supplemental Information [file mmc3.pdf]

Immunity

Article

The Heterogeneity of Ly6C^{hi} Monocytes Controls Their Differentiation into iNOS$^+$ Macrophages or Monocyte-Derived Dendritic Cells

Graphical Abstract

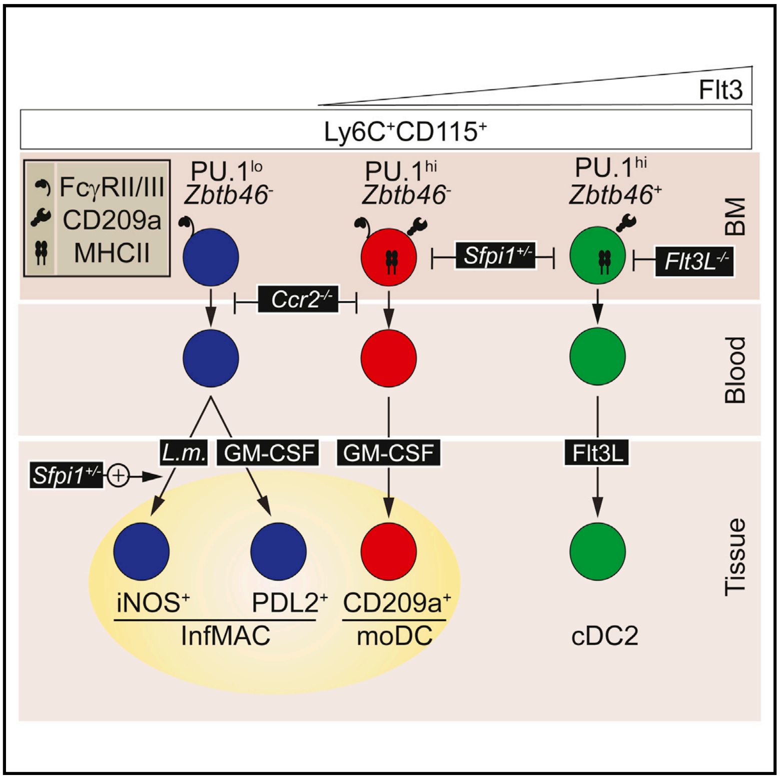

Authors

Shinelle Menezes, Daisy Melandri, Giorgio Anselmi, ..., Rachel Golub, Michel C. Nussenzweig, Pierre Guermonprez

Correspondence

pierre.guermonprez@kcl.ac.uk

In Brief

Monocytes can differentiate into multiple progenies during inflammation. Here, Menezes et al. show that monocytes from naive mice are heterogeneous and contain distinct precursor subsets giving rise to iNOS$^+$ inflammatory macrophages or GM-CSF-induced CD209a$^+$ monocyte-derived dendritic cells.

Highlights

- Murine Ly6C^{hi}CD115$^+$ monocytes are heterogeneous
- DC-related genes (*Cd209a* and *MHCII*) are expressed in a subset of FcγRIII$^+$ monocytes
- GM-CSF-dependent CD209a$^+$ moDCs are generated by FcγRIII$^+$CD209a$^+$MHCII$^+$ monocytes
- iNOS$^+$ macrophages are generated by FcγRIII$^+$CD209a$^-$MHCII$^-$ monocytes

Accession Numbers

GSE90471

Menezes et al., 2016, Immunity *45*, 1205–1218
December 20, 2016 © 2016 The Authors. Published by Elsevier Inc.
http://dx.doi.org/10.1016/j.immuni.2016.12.001

Immunity
Article

The Heterogeneity of Ly6C^{hi} Monocytes Controls Their Differentiation into iNOS^{+} Macrophages or Monocyte-Derived Dendritic Cells

Shinelle Menezes,[1,2,3] Daisy Melandri,[1,2,3] Giorgio Anselmi,[1,2,3] Thibaut Perchet,[4] Jakob Loschko,[5] Juan Dubrot,[6] Rajen Patel,[1,2,3] Emmanuel L. Gautier,[7] Stéphanie Hugues,[6] M. Paula Longhi,[8] Jake Y. Henry,[9] Sergio A. Quezada,[9] Grégoire Lauvau,[10] Ana-Maria Lennon-Duménil,[11] Enrique Gutiérrez-Martínez,[1,2,3] Alain Bessis,[12] Elisa Gomez-Perdiguero,[4] Christian E. Jacome-Galarza,[13] Hannah Garner,[13] Frederic Geissmann,[13] Rachel Golub,[4] Michel C. Nussenzweig,[5] and Pierre Guermonprez[1,2,3,14,*]
[1]Laboratory of Phagocyte Immunobiology
[2]Centre for Inflammation Biology and Cancer Immunology
[3]Peter Gorer Department of Immunobiology
King's College London, SE1 1UL London, UK
[4]Pasteur Institute, 75724 Paris, France
[5]The Rockefeller University, New York, NY 10065, USA
[6]Geneva University, 1211 Geneva, Switzerland
[7]Pitié-Salpêtrière Hospital, 75013 Paris, France
[8]Barts and the London School of Medicine, EC1M 6BQ London, UK
[9]University College London, WC1E 6BT London, UK
[10]Albert Einstein College of Medicine, New York, NY 10461, USA
[11]Institut Curie, 75248 Paris, France
[12]École Normale Supérieure, 75230 Paris, France
[13]Memorial Sloan Kettering Cancer Center, New York, NY 10065, USA
[14]Lead Contact
*Correspondence: pierre.guermonprez@kcl.ac.uk
http://dx.doi.org/10.1016/j.immuni.2016.12.001

SUMMARY

Inflammation triggers the differentiation of Ly6C^{hi} monocytes into microbicidal macrophages or monocyte-derived dendritic cells (moDCs). Yet, it is unclear whether environmental inflammatory cues control the polarization of monocytes toward each of these fates or whether specialized monocyte progenitor subsets exist before inflammation. Here, we have shown that naive monocytes are phenotypically heterogeneous and contain an NR4A1- and Flt3L-independent, CCR2-dependent, Flt3^{+}CD11c^{-}MHCII^{+}PU.1^{hi} subset. This subset acted as a precursor for FcγRIII^{+} PD-L2^{+}CD209a^{+}, GM-CSF-dependent moDCs but was distal from the DC lineage, as shown by fate-mapping experiments using *Zbtb46*. By contrast, Flt3^{-}CD11c^{-}MHCII^{-}PU.1^{lo} monocytes differentiated into FcγRIII^{+}PD-L2^{-}CD209a^{-}iNOS^{+} macrophages upon microbial stimulation. Importantly, *Sfpi1* haploinsufficiency genetically distinguished the precursor activities of monocytes toward moDCs or microbicidal macrophages. Indeed, *Sfpi1*$^{+/-}$ mice had reduced Flt3^{+}CD11c^{-}MHCII^{+} monocytes and GM-CSF-dependent FcγRIII^{+}PD-L2^{+}CD209a^{+} moDCs but generated iNOS^{+} macrophages more efficiently. Therefore, intercellular disparities of PU.1 expression within naive monocytes segregate progenitor activity for inflammatory iNOS^{+} macrophages or moDCs.

INTRODUCTION

Haematopoietic stem cells continually give rise to mononuclear phagocytes, including monocytes and conventional dendritic cells (DCs) (Steinman and Cohn, 1973). Both monocytes and DCs arise from common early bone marrow (BM) myeloid progenitors called MDPs (Fogg et al., 2006; Lee et al., 2015). MDPs further differentiate into (1) monocyte-committed progenitors (cMoPs) (Hettinger et al., 2013), giving rise to Ly6C^{+} monocytes unable to differentiate into DCs, and (2) common DC progenitors (CDPs) (Lee et al., 2015; Naik et al., 2007; Onai et al., 2007), which do not give rise to monocytes but generate circulating precursors for DCs (pre-DCs) (Breton et al., 2015; Liu et al., 2009). More recently, MDPs have been shown to generate granulocytes as well (Sathe et al., 2014).

Initially defined by their ability to drive the priming of naive T cells after activation (Nussenzweig et al., 1980), DCs are now regarded as a specific hematopoietic lineage defined by their dependency on growth factor Flt3L (McKenna et al., 2000), which engages the Flt3 receptor tyrosine kinase (CD135) (Waskow et al., 2008), and the expression of the transcription factor (TF) ZBTB46 (Meredith et al., 2012; Satpathy et al., 2012). Fate-mapping (Schraml et al., 2013) and barcoding (Naik et al., 2013) studies have firmly established that DCs are distinct from other lineages.

Monocytes are BM-derived mononuclear phagocytes that circulate in the blood stream. In mice, circulating monocytes are classically defined by expression of CD115 (CSF1R), a receptor for the macrophage growth factor CSF1 (M-CSF). Two categories of monocytes have been identified on the basis of

This is an open access article under the CC BY license (http://creativecommons.org/licenses/by/4.0/).

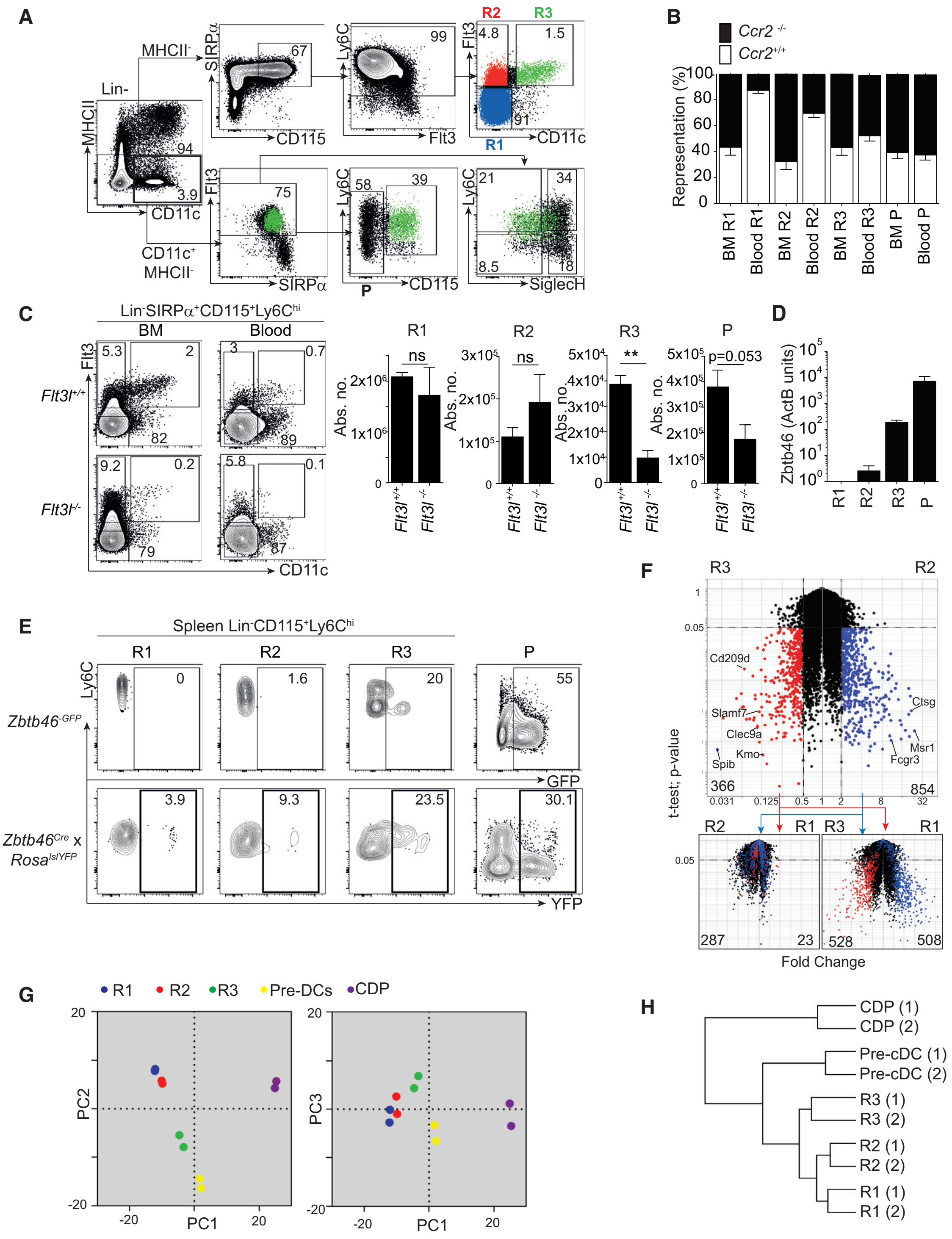

Figure 1. Identification of BM Ly6C$^+$CD115$^+$ Subsets

(A) BM Lin$^-$MHCII$^-$Ly6C$^+$CD115$^+$ monocytes consist of three subsets. Shown is a representative flow cytometry analysis of WT BM at steady state. (Top) Lineage$^-$ (CD19$^-$, CD3ε$^-$, Ly6G$^-$, Ter119$^-$, CD45RA$^-$, NK1.1$^-$, cKit$^-$) MHCII$^-$ cells analyzed by a conventional monocyte gating strategy. CD115$^+$Ly6C^{hi} cells can be subdivided into three sub-populations: R1(Flt3$^-$CD11c$^-$), R2(Flt3$^+$CD11c$^-$), and R3(Flt3$^+$CD11c$^+$). (Bottom) Pre-DC gating of Lin$^-$MHCII$^-$CD11c$^+$ Flt3$^+$SIRPα^{int} cells. Pre-DCs can be subdivided into CD115$^-$ (P) and CD115$^+$(R3) subsets. Overlay of R3 is shown in green in the lower panels.

(B) Graphical summary of WT and *Ccr2*$^{-/-}$ mixed BM chimera. Shown are steady-state percentages of WT (CD45.1, white bars) and *Ccr2*$^{-/-}$ (CD45.2, black bars) cells within R1–R3 and P in the BM and blood of WT (CD45.1) reconstituted recipients. Data represent five chimeric mice over two experiments.

(legend continued on next page)

the expression of Ly6C and CX3CR1 according to GFP intensity in *Cx3cr1*$^{GFP/+}$ mice: Ly6C^{+}CX3CR1^{int} and Ly6C^{-}CX3CR1^{hi} monocytes (Geissmann et al., 2003). Various studies support the notion that Ly6C^{+} monocytes can convert to blood Ly6C^{-} monocytes (Hettinger et al., 2013; Sunderkötter et al., 2004; Varol et al., 2007; Yona et al., 2013). However, selective impairment of Ly6C^{+} monocytes in *Irf8*$^{-/-}$ mutant mice suggests an independent developmental pathway for Ly6C^{-} monocytes (Kurotaki et al., 2013). The egress of BM Ly6C^{+} monocytes at steady state requires the engagement of the chemokine receptor CCR2 (Serbina and Pamer, 2006). By contrast, most Ly6C^{-} monocytes gain access to the bloodstream independently of CCR2 and rely on the TF NR4A1 (Hanna et al., 2012). They exhibit a "patrolling" behavior (Auffray et al., 2007) and scavenge damaged endothelia during inflammation (Carlin et al., 2013). A subset of Ly6C^{-} monocytes expressing extracellular major histocompatibility complex II (MHCII) has also been described (Jakubzick et al., 2013).

Inflammatory monocytes have multiple fates. Pamer and colleagues have elegantly shown that the sensing of *Listeria monocytogenes* (*L.m.*) infection activates the release of CCR2 ligands to mediate the recruitment of Ly6C^{+} monocytes, which differentiate into TNF-α^{+}iNOS^{+} microbicidal phagocytes (Serbina et al., 2003). iNOS^{+}Ly6C^{+} phagocytes are distinct from the DC lineage (Meredith et al., 2012; Satpathy et al., 2012) and are essential for the control of *Listeria* infection, as demonstrated by infection of *Nos2*$^{-/-}$ (MacMicking et al., 1995), *Ccr2*$^{-/-}$ (Serbina et al., 2003), and monocyte-depleted (Schreiber et al., 2013) mice.

In addition to differentiating into iNOS^{+} phagocytes, Ly6C^{+} monocytes can differentiate into CCR2-dependent monocyte-derived DCs (moDCs) (Bain et al., 2013; Zigmond et al., 2012). Accordingly, moDCs can be generated upon adoptive transfer of Ly6C^{+} monocytes that progressively lose Ly6C and acquire MHCII when differentiating in inflamed tissues (Bain et al., 2013; Zigmond et al., 2012). FcγRI (CD64), FcεRI, and CD206 have emerged as markers of inflammatory phagocytes distinct from the DC lineage (Cheong et al., 2010; Langlet et al., 2012; Plantinga et al., 2013).

The processes regulating the polarization of Ly6C^{+} monocytes toward iNOS^{+} macrophages or moDCs remain unclear. Local inflammatory cues might control the nature of monocyte progeny. Alternatively, monocyte subpopulations might be endowed with a selective potential to generate iNOS^{+} phagocytes or moDCs. Here, we report the description and functional characterization of monocyte subsets endowed with the selective ability to generate iNOS^{+} phagocytes or moDCs. We show that the amount of PU.1 arbitrates the commitment of monocytes toward either cell fate.

RESULTS

Ly6C^{+} Monocytes Are Heterogeneous

As an initial approach to addressing the heterogeneity of BM mononuclear phagocyte precursors, we analyzed the expression of CD135 (Flt3) and CD115 (CSF1R) in Lin^{-} BM cells (Figure 1A; isotype controls in Figure S1A). MHCII^{+} cells were not considered because they correspond to F4/80^{hi} BM macrophages (Figure S1B). We noticed that MHCII^{-}CD172a (SIRPα)$^{+}$ CD115^{+}Ly6C^{+} cells contained three sub-populations: a major CD11c^{-}Flt3^{-} (R1) and two minor CD11c^{-}Flt3^{+} (R2) and CD11c^{+} Flt3^{+} (R3) populations (Figures 1A and S1C). R3 corresponded phenotypically to a subset of pre-DCs (CD11c^{+}Flt3^{+}SIRPα^{int}; Liu et al., 2009; Figure 1A) and was distinct from cKit^{+}CD115^{+} Flt3^{+} CDPs (Naik et al., 2007; Onai et al., 2007; Figure S1D). Within pre-DCs, R3 coexisted with CD115^{-} pre-DCs (P) and aligned with both the Ly6C^{+}SiglecH^{-} and Ly6C^{+}SiglecH^{+} pre-DC subsets (Figure 1A; Schlitzer et al., 2015). R1 and R2 monocytes expressed heterogeneous amounts of CD11b and CX3CR1 (Figure S1E), had horse-shoe-shaped nuclei (Figure S1F), and were distinct from *Nr4a1*-dependent Ly6C^{lo} monocytes (Figure S1G).

Blood Lin^{-}CD115^{+}Ly6C^{+} cells, like their Ly6C^{+} BM counterparts, also contained sub-populations R1–R3 (Figures 1B and 1C). R1 expressed higher amounts of CCR2 than R2 and R3 (Figure S1E), and *Ccr2* inactivation drastically restricted the size of circulating R1 and R2 monocytes, but not pre-DCs (Figure S1H). Mixed BM chimeras of wild-type (WT) and *Ccr2*$^{-/-}$ cells showed that CCR2 controls the egress of R1 and R2 monocytes by a cell-intrinsic effect (Figures 1B and S1I).

Unlike pre-DCs and DCs (McKenna et al., 2000), BM and blood R1 and R2 cells were largely independent of growth factor Flt3L (Figure 1C). The DC-specific TF-encoding gene *Zbtb46* (BTBD4) was highly expressed only in R3 and P pre-DCs (Figure 1D). Accordingly, reporter expression in *Zbtb46*$^{GFP/+}$ mice and fate mapping in the *Zbtb46*^{Cre} *x Rosa*^{lslYFP} model (Loschko et al., 2016; Figures 1E and S1J) showed that splenic R3 and P pre-DCs, but not R1 or R2 monocytes, belonged to the DC lineage.

Genes with higher expression in R3 pre-DCs than in R2 monocytes largely overlapped the genes with higher expression in R3 pre-DCs than in R1 monocytes (e.g., *Clec9a* and *Slamf7*; Figure 1F, red dots; Table S1) and, to a lesser extent, overlapped genes with higher expression in R2 monocytes than in R1 monocytes (*Ctsg* and *Flt3*; Figure 1F, red dots in lower left plot). R3

(C) Flow cytometry analysis of R1–R3 and P in *Flt3l*$^{-/-}$ and *Flt3l*$^{+/+}$ BM and blood. Quantification shows the absolute number of each cell population within the blood (n = 4 mice per group).

(D) qPCR for *Zbtb46* in WT BM R1–R3 and P in β-actin units.

(E) *Zbtb46* labeling and reporter expression. Steady-state GFP reporter expression and YFP expression in splenic R1–R3 and P cells in *Zbtb46*$^{GFP/+}$ and *Zbtb46*^{Cre} *x Rosa*^{lslYFP} mice, respectively.

(F) Genes differentially expressed among R1–R3. Volcano plots of R2 versus R3 (main plot) show genes with a fold change $\geq$ 2 and a p value of $p < 0.05$ in R2 (blue) and R3 (red). These genes are overlaid on volcano plots of R1 versus R2 (left) and R1 versus R3 (right) with the same axes of fold change and p value. Numbers indicate differentially expressed genes in each comparison (gene list available in Table S1).

(G) Clustering of R1–R3 with pre-DCs and CDPs. Principal-component analysis compares microarray data of R1 (blue), R2 (red), and R3 (green) with previously published data of pre-DCs (yellow) and CDPs (violet) on PC1 (72% variance), PC2 (24% variance), and PC3 (2% variance).

(H) Hierarchical clustering analysis (1 − Pearson correlation) of monocytes (R1 and R2), pre-DCs (R3), total pre-DCs, and CDPs.

Data represent the mean ± SEM (*p < 0.05;**p < 0.005; ns, not significant; Student's t test). Please also refer to Figure S1.

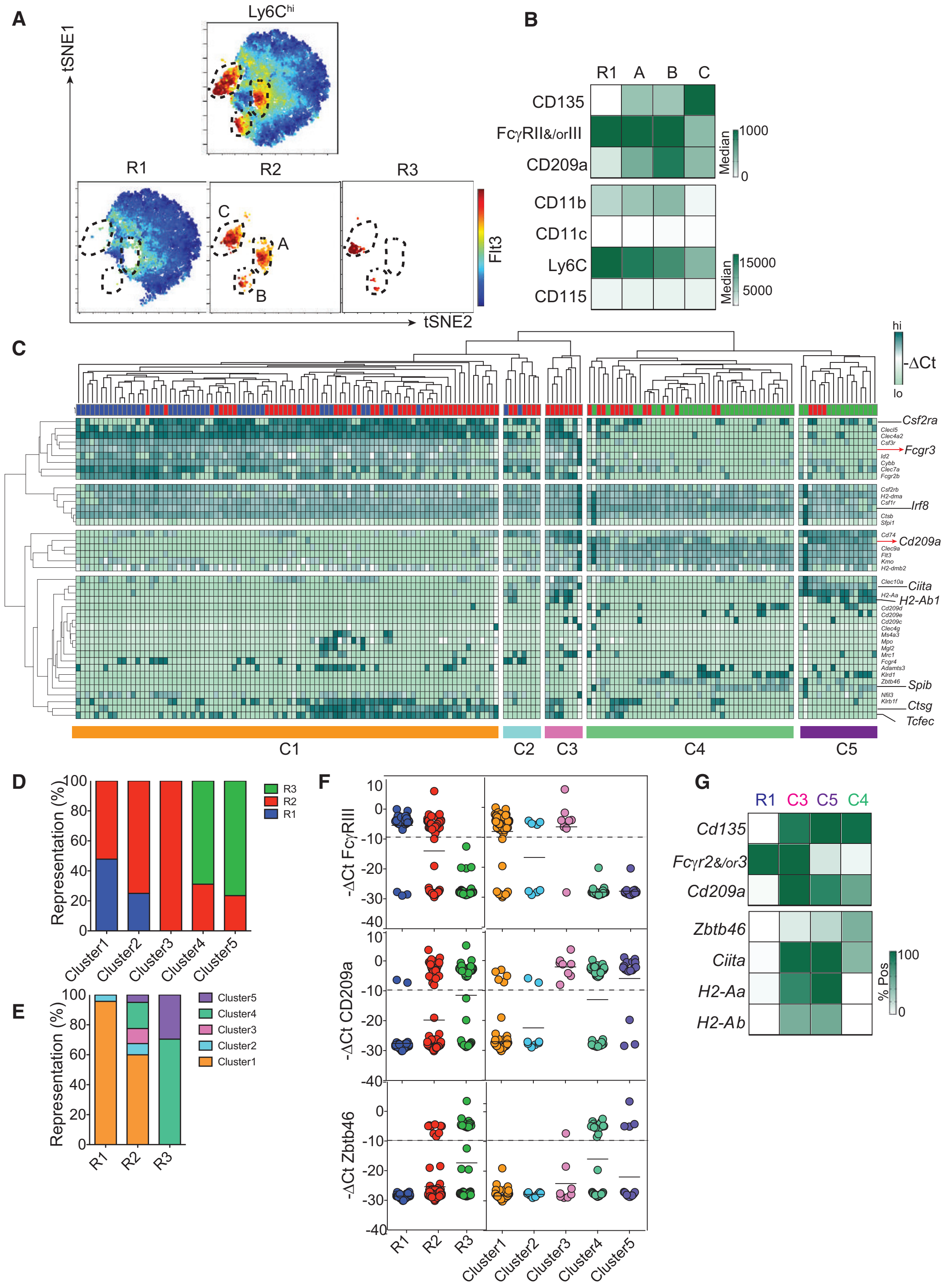

Figure 2. Transcriptional Profiling of Monocyte Subsets R1 and R2

(A) Nonlinear dimensionality reduction analysis of Ly6C^{hi}CD115^{+}cells. t-SNE maps of total Lin^{-}MHCII^{-}CD115^{+}SIRPα^{+} cells, R1 and R2 monocytes, and R3 pre-DCs are based on the parameters CD115, SIRPα, Ly6C, Flt3, CD11c, CD209a, and FcγRII and/or FcγRIII. Color scale indicates Flt3 expression.

(B) Expression analysis of t-SNE-generated sub-populations of R2 and expression of Flt3, FcγRII and/or FcγRIII, CD209a, CD11b, CD11c, Ly6C, and CD115 of t-SNE-generated Flt3^{+} populations A–C with R1 monocytes. CD209a expression is shown as the difference between MFI and fluorescence minus one (FMO) control for all four populations.

(legend continued on next page)

pre-DCs expressed genes belonging to the DC signature (Miller et al., 2012) (e.g., *Clec9a* and *Slamf7*; Figures 1F and S1K) and clustered with total pre-DCs and CDPs (Figures 1G and 1H).

Genes with higher expression in R2 monocytes than in R3 pre-DCs (Figure 1F, blue dots in the main panel) largely overlapped the genes with higher expression in R1 monocytes than in R3 pre-DCs (e.g., *Msr1* and *Fcgr3*; Figure 1F, lower right plot; Table S1). However, most of these genes were not differentially expressed between R2 and R1 monocytes (Figure 1F, blue dots in lower left panel). Furthermore, R1 and R2 expressed a macrophage signature (e.g., *Fcgr3* and *Csf3r*; Figure S1K; Gautier et al., 2012) while clustering close to each other (Figures 1G and 1H) apart from the DC-committed precursors.

Overall, R1 and R2 were more similar to each other than to R3 (Figures 1F–1H; Table S1). We conclude that R1 and R2 qualify as bona fide monocytes given that both are largely CCR2 dependent for BM egress, do not rely on Flt3L, and do not express *Zbtb46*. R3 met all the criteria for bona fide pre-DCs because it was largely CCR2 independent and Flt3L dependent and expressed the DC-specific *Zbtb46* (Meredith et al., 2012; Satpathy et al., 2012).

R2 Monocytes Bear a Mixed Transcriptional Profile

We next aimed to assess the diversity of Ly6C$^+$ monocytes by using unsupervised analyses. To this end, we used multi-dimensional reduction analysis of multi-parametric flow cytometry. BM Ly6C$^+$CD115$^+$ cells were divisible into one major Flt3$^-$ and three minor Flt3$^+$ subsets with distinct t-distributed stochastic neighbor embedding (t-SNE) coordinates (populations A–C) (Figure 2A). Population A was CD11c$^-$ and found only within R2. By contrast, populations B and C overlapped R2 and R3. Unlike population C, A and B phenotypically shared high expression of FcγRII and/or FcγRIII with R1 monocytes. However, like population C but unlike R1 monocytes, A and B expressed CD209a (Figure 2B). All together, these data were corroborated by microarray analysis of BM R1–R3 populations (Figure S2A) and flow cytometry analysis of blood Ly6C$^+$ cells (Figure S2B). In addition, population C had lower CD11b expression than A and B (Figure 2B).

As a parallel unsupervised approach, we used gene-expression profiling at the single-cell level to assess the diversity of Ly6C$^+$CD115$^+$ cells. We performed single-cell qPCR by using a set of 42 genes and 3 house-keeping controls. Unsupervised clustering of gene expression at the single-cell level revealed the existence of five clusters within Ly6C$^+$CD115$^+$ cells (Figure 2C). Clusters 1 and 2 aligned mostly with R1 and also partially with R2. Cluster 3 was exclusively represented within R2, whereas clusters 4 and 5 were enriched in R3 but also present in R2 (Figures 2C–2E). Cluster 3 was unique in its mixed expression pattern of monocyte (*Fcgr3*, *Fcgr2b*, and *Csf3r*) and DC (*Kmo*, *Cd209a*, and *Flt3*) genes (Figures 2C and 2F and S2A and S2B). However, cluster 3 (in addition to clusters 1 and 2) showed low transcription of *Zbtb46*, which was found in clusters 4 and 5 (Figure 2F).

Of interest, we noticed that the expression of *Ciita* and MHCII-related genes was found mostly in clusters 3 and 5 (Figure S2C). This is consistent with MHCII expression in BM and blood R2 and R3, as assessed by flow cytometry and microarray analysis (Figures S2D and S2E). R2 and R3 thus aligned with previously described Ly6C$^+$CD115$^+$MHCII$^+$ cells in the blood (Carlin et al., 2013; Jakubzick et al., 2013; Figure S2F). Using mice deficient of various CIITA promoters (pI$^{-/-}$, pIV$^{-/-}$, or pIII$^+$pIV$^{-/-}$), we showed that R2, like DCs, expressed MHCII after pI-dependent induction of *Ciita* (LeibundGut-Landmann et al., 2004; Figures S2G and S2H).

All together, both unsupervised flow cytometry and gene-expression analyses revealed that R2 monocytes contained unique populations of *Zbtb46*$^-$ cells that were distinct from the cDC lineage and expressed transcriptional profiles with mixed features of monocytes and cDCs (*Zbtb46*$^-$Flt3$^+$FcγRII and/or FcγRIII$^+$CD209a$^+$CD11c$^-$). Using MHCII as a surrogate marker for these cells, we showed that they are independent of Flt3L and rely on CCR2 for their mobilization from the BM to the blood (Figures S2I and S2J).

PU.1 Controls the Formation of Flt3$^+$MHCII$^+$ R2 Monocytes

We next sought to identify TFs regulating the formation of R2 Flt3$^+$MHCII$^+$ monocytes. *Sfpi1* (PU.1) is an attractive candidate because it promotes Flt3 expression (Carotta et al., 2010) and MHCII through the induction of *Ciita* (Bakri et al., 2005). Intracellular flow cytometry staining for PU.1 indicated that R3 pre-DCs and R2 monocytes expressed higher amounts of PU.1 than R1 monocytes (Figure 3A). Accordingly, PU.1 was expressed more in MHCII$^+$ than in MHCII$^-$ blood CD115$^+$ cells (Figure 3B).

The effects of PU.1 were tightly dependent on its expression; therefore, we analyzed *Sfpi1*$^{+/-}$ mice that had reached adulthood without any obvious phenotype. *Sfpi1*$^{+/-}$ mice had reduced numbers of blood CD115$^+$Ly6C^{lo} cells, whereas blood Ly6C$^+$ were not affected despite an increase in BM Ly6C$^+$ cells (Figure 3C). *Sfpi1*$^{+/-}$ mice had reduced MHCII$^+$ (Ly6C$^+$ and Ly6C$^-$) CD115$^+$ populations in the BM and blood (Figure 3D). Finally, *Sfpi1*$^{+/-}$ mice had smaller populations of R2 monocytes (including the MHCII$^+$ fraction of R2) and R3 pre-DCs than WT mice in BM and blood (Figures 3E and S3A–S3C). In agreement with R3 pre-DC reduction, *Sfpi1*$^{+/-}$ mice displayed a selective deficiency in spleen CD11b$^+$ DCs, mostly in the ESAM1^{lo}Flt3^{lo} compartment, whereas plasmacytoid DCs remained unchanged (Figures S3D–S3F). In contrast to R2 monocytes and R3

(C) Heterogeneity in population R2. The hierarchical clustering dendogram (top and left margins) is based on $-\Delta$Ct values from single-cell multiplex qPCR analysis of 44 R1 (blue), 81 R2 (red), and 44 R3 (green) single cells for 42 genes.

(D) Representation of populations R1–R3 within the five clusters defined in (C).

(E) Representation of the five clusters within populations R1–R3.

(F) Single-cell expression of *Fcgr3*, *CD209a*, and *Zbtb46* in populations R1–R3 or clusters C1–C5. Each dot represents the $-\Delta$Ct value of a single cell.

(G) Analysis of the defining genes of population R1 with clusters C3–C5. The heatmap compares mRNA expression of MHCII-related genes *Cd135*, *Zbtb46*, *Cd209a*, and *Fcgr2* and/or *Fcgr3* on single cells between clusters C3–C5 and monocyte population R1.

Please also refer to Figure S2.

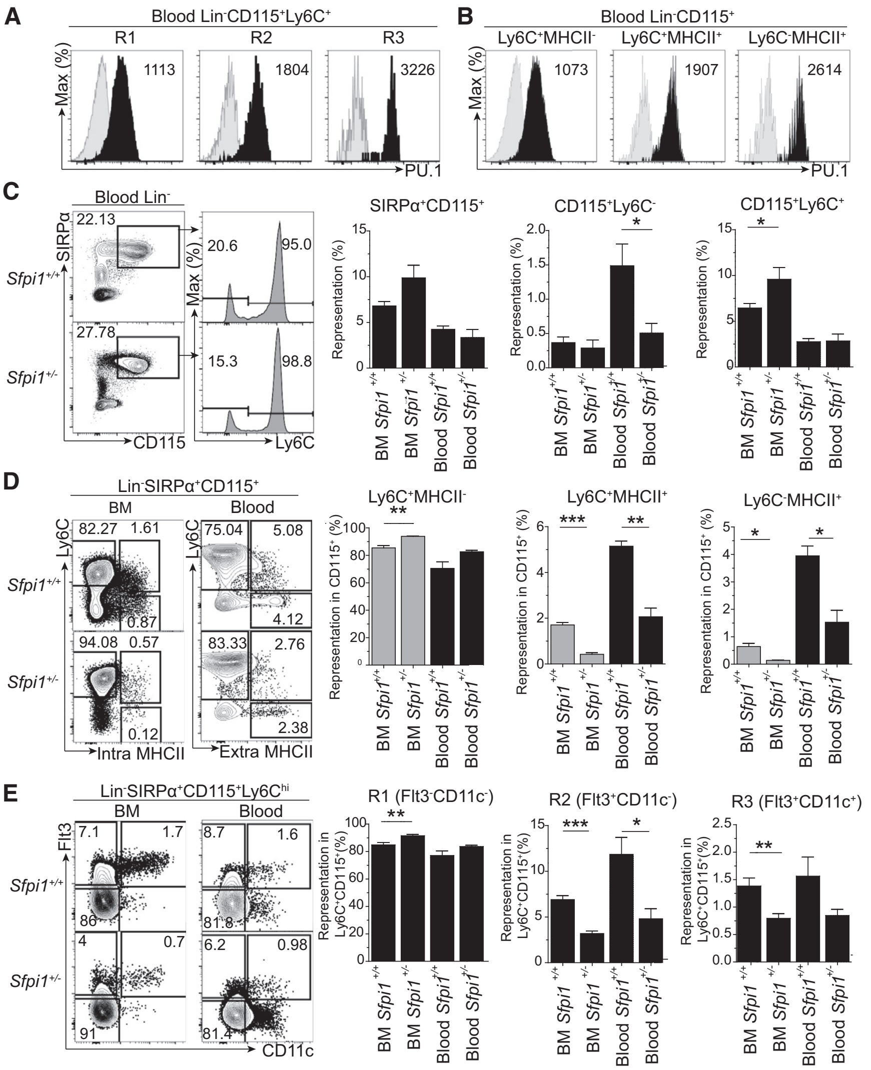

Figure 3. PU.1 Controls the Development of PU.1^{hi}Flt3$^+$MHCII$^+$ R2 Monocytes at Steady State

(A and B) Expression of PU.1 (black shading) and isotype control (gray shading) within Ly6C^{hi}CD115$^+$ monocytes (R1 and R2) and pre-DCs (R3) (A) and in Ly6C^{hi}MHCII$^-$, Ly6C$^+$MHCII$^+$, and Ly6C$^-$MHCII$^+$CD115$^+$ cells (B) in the blood as seen by intra-nuclear staining of PU1 by flow cytometry. Numbers within plots indicate the MFI of PU.1.

(C–E) Representative flow cytometric analysis of the blood of *Sfpi1*$^{+/+}$ and *Sfpi1*$^{+/-}$ mice at steady state. (C) Comparison of total (SIRPα$^+$CD115$^+$), Ly6C$^+$, and Ly6C^{lo} blood monocytes by flow cytometry and quantification as the percentage of total live cells. (D and E) Comparison of Ly6C$^+$ MHCII$^-$, Ly6C$^+$MHCII$^+$, and Ly6C$^-$MHCII$^+$ cells within SIRPα$^+$CD115$^+$ monocytes (D) and of R1–R3 within Ly6C^{hi} MHCII$^{+/-}$ monocytes (E) in the BM and blood. Gray bars indicate intracellular staining for MHCII.

Data represent the mean ± SEM of three mice per group from three identical experiments (*p < 0.05,**p < 0.005, ***p < 0.0005; Student's t test). Please also refer to Figure S3.

pre-DCs, R1 monocytes were slightly increased in the BM of *Sfpi1*$^{+/-}$ mice (Figure 3E). However, this increase did not reach significance in the blood (Figures 3E, S3A, and S3B). *Sfpi1* hemizygosity reduced the numbers of MDPs and CDPs, but not cMoPs (Figure S3F). We conclude that high expression of PU.1 is selectively required for the development of Flt3$^+$MHCII$^+$ R2 monocytes and R3 pre-DCs at steady state.

PU.1^{lo}Flt3$^-$MHCII$^-$ R1 Monocytes Generate iNOS$^+$ Macrophages upon Microbial Stimulation

In the next set of experiments, we aimed to determine which Ly6C$^+$ monocytes are precursors for iNOS$^+$ macrophages. We chose to use *Listeria* infection because it efficiently induces the recruitment and differentiation of iNOS$^+$ phagocytes from Ly6C$^+$ monocytes (Serbina et al., 2003). In agreement with their independence of Flt3L (Meredith et al., 2012), *L.m.*-induced iNOS$^+$ macrophages were not identified in *Zbtb46*^{cre}x*ROSA*^{lslYFP} mice (Loschko et al., 2016), thus confirming their monocytic origin (Figure S4A). In vitro exposure to lipopolysaccharide (LPS) or infection with *L.m.* resulted in iNOS expression in a subset of responding BM CD115$^+$Ly6C$^+$ cells (up to 38%) that remained mostly MHCII^{lo} (Figure 4A), unless these cells were treated with interferon-γ (IFN-γ), which increased MHCII expression in iNOS$^+$ cells (Figure S4B). Overnight culture of sorted R1 or R2 monocytes or R3 pre-DCs with *L.m.* or LPS (Figures 4B and S4C) revealed the selective ability of R1 to produce iNOS$^+$ MHCII$^-$ macrophages, whereas R2 and R3 generated iNOS$^-$MHCII$^+$ cells (Figures 4B and S4C). We conclude that the formation of iNOS$^+$ macrophages upon microbial exposure is a selective feature of R1 monocytes or possibly a subset of them. Like R1 monocytes (Figures 2F and 2G), in-vivo-generated Ly6C$^+$CD11b$^+$iNOS$^+$ macrophages (Serbina et al., 2003) expressed FcγRII and/or FcγRIII, but not CD209a or Flt3 (Figure 4C).

Transcriptomic analysis of BM R1–R3 demonstrated an upregulation of toll-like receptor (TLR) signaling, RIG-like helicase, NOD-like receptor genes, and IFN-γ signaling and its target genes in R1 monocytes (Figure 4D). We conclude that R1 cells are efficiently equipped for innate sensing prior to microbial exposure.

We next wanted to determine whether iNOS$^+$ macrophages lose iNOS and enter a differentiation pathway of monocytes characterized by the loss of Ly6C in inflamed tissues (Bain et al., 2013; Zigmond et al., 2012). We used a fate-mapping approach to irreversibly label iNOS-expressing cells in *Nos2*$^{tomato\text{-}cre}$x*ROSA*^{lsltdTomato} mice. *L.m.* infection triggered the labeling (Tomato$^+$) of a subset of Ly6C$^+$ cells 2 days after infection; these cells were also stained with anti-iNOS antibody (Figures 4E, 4F, and S4D). We found that by day 4, the iNOS$^+$ cells (both Tomato$^+$ and anti-iNOS-Ab$^+$ cells) had reduced drastically

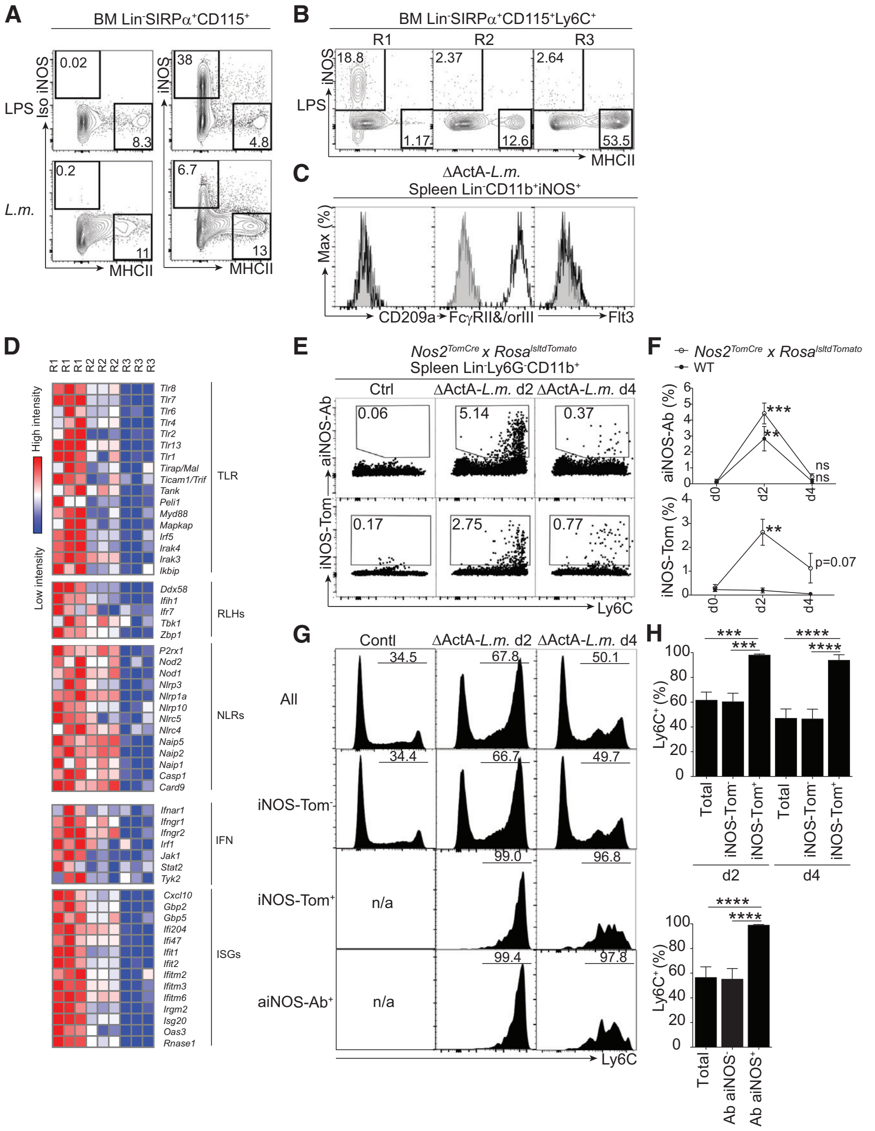

Figure 4. $Pu.1^{lo}Flt3^{-}MHCII^{-}$ R1 Monocytes Differentiate into $iNOS^{+}$ Phagocytes upon Microbial Stimulation

(A) iNOS production by $CD115^{+}$ cells in vitro. Shown is surface MHCII and intracellular iNOS or isotype control (Iso iNOS) staining after overnight culture of BM $Lin^{-}CD115^{+}$ cells in the presence of LPS or *L.m.* (MOI = 0.1).

(B) In vitro microbial stimulation of R1–R3. Shown is the analysis of surface MHCII and intracellular iNOS on sorted R1–R3 cells cultured overnight in the presence of LPS (1 μg/ml).

(C) Cell-surface phenotype of $Lin^{-}CD11b^{+}iNOS^{+}$ cells during *L.m.* infection (day 2). Shown is flow cytometry analysis of CD209a, FcγRII and/or FcγRIII (CD16/32), and Flt3 (black lines) against respective isotype controls (gray shading).

(D) Pathway analysis of differentially expressed genes in flow-cytometry-sorted steady-state BM R1–R3. Abbreviations are as follows: TLR, toll-like receptor; RLH, RIG-like helicase; NLR, NOD-like receptor; IFN, interferon; and ISG, interferon-stimulated gene.

(E–H) Fate mapping of *L.m.*-induced iNOS-expressing splenocytes. (E) $Nos2^{TomatoCre}$ x $Rosa^{lsltdTomato}$ mice infected with the ΔActA mutant of *L.m.* were analyzed for intracellular anti-iNOS staining (top) and tomato labeling (bottom) in $Lin^{-}CD11b^{+}$ splenocytes in control or *L.m.*-infected mice (days 2 and 4). (F) Mean ± SEM of the percentage of iNOS-Ab^{+} (top) and $Tomato^{+}$ (bottom) cells in Bl/6 (WT) or $Nos2^{TomatoCre}$ x $Rosa^{lsltdTomato}$ mice in untreated (d0) or *L.m.*-infected mice on days 2 and 4 (n = 3). (G) Histograms show Ly6C expression in cells either stained or unstained with anti-iNOS antibody or iNOS-Tomato. (H) Percentages of $Ly6C^{+}$ cells within each indicated population (n = 4 mice per group; $^{**}p < 0.005$, $^{***}p < 0.0005$, $^{****}p < 0.00005$; Student's t test).

Please also refer to Figure S4.

(Figure 4F). Like iNOS-Ab^{+} cells, $tomato^{+}$ cells remained $Ly6C^{+}$ and did not become $Ly6C^{-}$ (Figures 4G and 4H). Therefore, we conclude that $Ly6C^{+}iNOS^{+}$ macrophages do not convert efficiently to $Ly6C^{-}$. This result is compatible with the existence of a subset of $Ly6C^{+}$ monocytes specialized to generate $iNOS^{+}$ macrophages.

High Amounts of PU.1 Inhibit the Production of $iNOS^{+}$ Macrophages

From the experiments performed so far, we noticed that PU.1 concentration was inversely correlated with the ability of monocyte subsets to generate $iNOS^{+}$ macrophages (Figures 3A and 4B). We therefore hypothesized that PU.1 acts as a regulator of iNOS expression. To test this hypothesis, we infected WT and $Sfpi1^{+/-}$ mice with *L.m.* and analyzed the generation of $CD11b^{+}Ly6C^{+}iNOS^{+}$ macrophages in the spleens of these animals 2 days later. Using the avirulent ΔActA mutant of *L.m.*, we were able to assess innate sensing of the bacteria independently of infectious load (Schreiber et al., 2013; Serbina et al., 2003). $Sfpi1^{+/-}$ mice accumulated higher numbers of total $CD11b^{+}$ $Ly6C^{+}$ and $CD11b^{+}Ly6C^{+}iNOS^{+}$ macrophages than did their WT counterparts (Figures 5A–5C). Importantly, the percentage of $iNOS^{+}$ cells in $Ly6C^{+}CD11b^{+}$ macrophages was increased in $Sfpi1^{+/-}$ mice, whereas MHCII expression remained unchanged (Figures 5C and S5A).

In order to test whether the regulatory role of PU.1 is cell intrinsic, we infected normalized numbers of BM macrophages (BMDMs) from WT or $Sfpi1^{+/-}$ mice infected with *L.m.* (or LPS) overnight. We found that $Sfpi1^{+/-}$ macrophages expressed higher amounts of iNOS than did WT cells both in percentage and staining intensity (Figures 5D and S5B). We conclude that *Sfpi1* acts as a negative regulator of iNOS acquisition in monocytes responding to microbial stimulation.

In order to address the cell-intrinsic role of PU.1 in vivo, we performed adoptive transfers of $CD45.2^{+}Sfpi1^{+/+}$ or $CD45.2^{+}$ $Sfpi1^{+/-}$ BM cells into *L.m.*-infected $CD45.1^{+}$ recipients. $Sfpi1^{+/-}$ donor cells expressed more iNOS than their WT counterparts

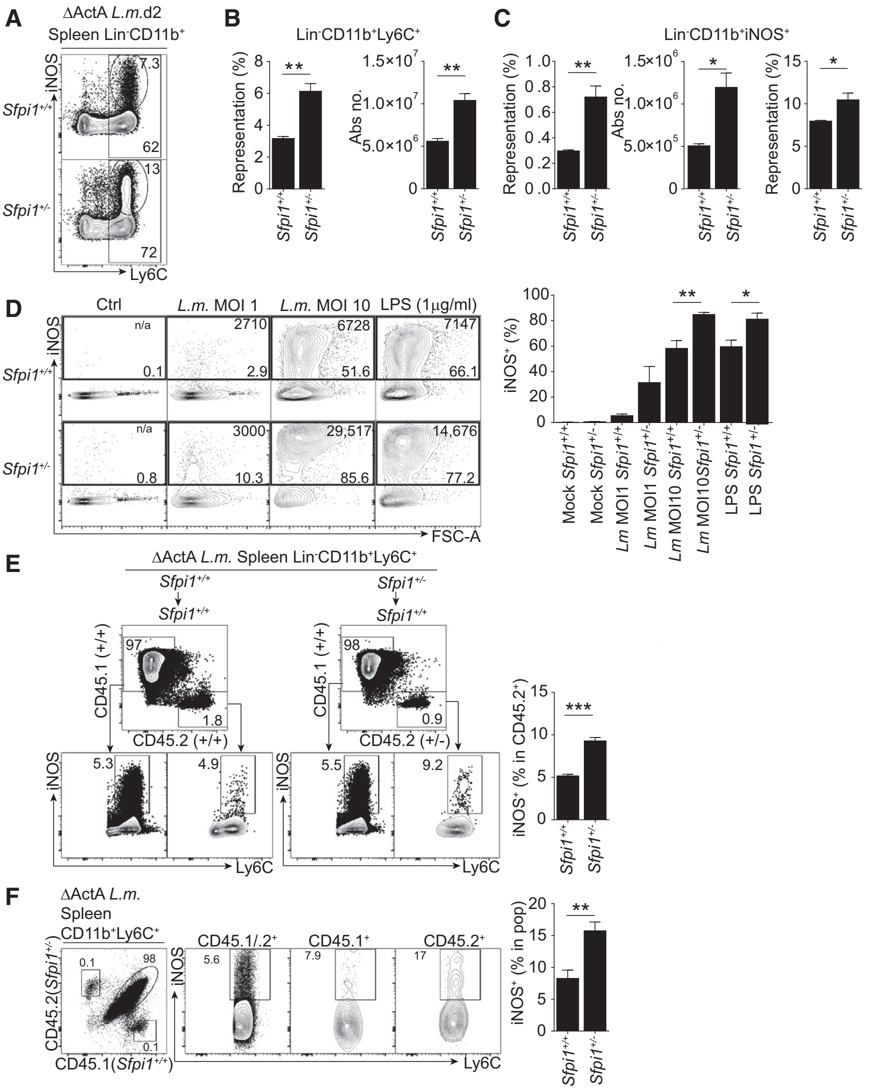

Figure 5. PU.1 Inhibits the Generation of iNOS$^+$ Macrophages upon Microbial Stimulation

(A–C) Generation of Ly6C$^+$iNOS$^+$ cells upon microbial stimulation of *Sfpi1*$^{+/+}$ or *Sfpi1*$^{+/-}$ mice.

(A) Intracellular flow cytometry staining of spleen Lin$^-$ (CD19$^-$, CD3ε$^-$, Ly6G$^-$, Ter119$^-$, CD45RA$^-$, NK1.1$^-$, cKit$^-$) CD11b$^+$ cells in *Sfpi1*$^{+/+}$ and *Sfpi1*$^{+/-}$ mice infected with ΔActA *L.m.* (B and C) Quantification of the percentage and absolute number of Lin$^-$CD11b$^+$Ly6C$^+$ cells (B) and Lin$^-$CD11b$^+$iNOS$^+$ cells (C).

(D) Microbial stimulation of BMDMs generated from *Sfpi1*$^{+/+}$ or *Sfpi1*$^{+/-}$ mice and flow cytometry analysis of iNOS$^+$ BMDMs from *Sfpi1*$^{+/+}$ and *Sfpi1*$^{+/-}$ mice. BMDMs were cultured with MCSF alone, MCSF and *L.m.* at a MOI of 1 or 10, or MCSF and 1 μg/ml LPS. Numbers at the top of each gate indicate MFI. The percentage of iNOS$^+$ cells obtained within each culture is quantified.

(E) PU.1 reduction in the BM compartment results in an increase in iNOS$^+$ macrophages in vivo. CD45.2$^+$*Sfpi1*$^{+/+}$ or *Sfpi1*$^{+/-}$ whole BM was adoptively transferred into WT ΔActA *L.m.*-infected CD45.1$^+$ recipients. Representative flow cytometry analysis of iNOS expression in Lin$^-$CD11b$^+$Ly6C$^+$ splenocytes from recipients (CD45.1$^+$) or donors (CD45.2$^+$) is shown.

(F) PU.1 in monocytes regulates the production of iNOS$^+$ macrophages in vivo. Shown is flow cytometry analysis of iNOS expression in Lin$^-$CD11b$^+$Ly6C$^+$ cells of CD45.1$^+$*Sfpi1*$^{+/+}$ and CD45.2$^+$*Sfpi1*$^{+/-}$ sorted BM monocytes adoptively transferred intravenously into WT ΔActA *L.m.*-infected CD45.1/.2$^+$ recipients (n = 4 mice per group).

Data represent the mean ± SEM (*p < 0.05; **p < 0.005; ***p < 0.0005; ns, not significant; Student's t test). Please also refer to Figure S5.

(Figure 5E). Finally, to address the role of *Sfpi1* in the monocyte compartment, we engrafted a mixture of *Sfpi1*$^{+/-}$ (CD45.2) and *Sfpi1*$^{+/+}$ (CD45.1) BM monocytes into *L.m.*-infected CD45.1 and CD45.2 recipient mice. We found that the percentage of iNOS$^+$ macrophages within Ly6C$^+$CD11b$^+$ cells was higher in the progeny of *Sfpi1*$^{+/-}$ monocytes than in that of *Sfpi1*$^{+/+}$ monocytes (Figure 5F).

We conclude that PU.1 acts as a cell-intrinsic negative regulator of the differentiation of monocytes into iNOS$^+$ macrophages upon microbial exposure.

PU.1^{hi}Flt3$^+$MHCII$^+$ R2 Monocytes Differentiate into PDL2$^+$CD209a$^+$ moDCs upon GM-CSF Exposure

Having established that R1 monocytes are poised to generate iNOS$^+$ macrophages, we next assessed the ability of monocyte subsets to give rise to moDCs. Elevation of granulocyte-macrophage colony-stimulating factor (GM-CSF) concentrations induces the accumulation of CD11b$^+$MHCII$^+$ spleen cells that resemble CD11b$^+$ DCs (Daro et al., 2000; Mach et al., 2000). Engraftment of live GM-CSF-producing B16 melanoma (B16-GM-CSF) (Dranoff et al., 1993) triggered the expansion of (1) circulating MHCII$^+$ monocytes with varying expression levels of Ly6C (Figure S6A) and (2) Lin$^-$Ly6G$^-$CD11b$^+$Ly6C$^{hi–lo}$MHCII$^+$ spleen cells largely overlapping the CD11b$^+$ DC phenotype (Figure S6B). GM-CSF-induced (or LPS-induced; Figure S6C) Ly6C$^{hi–lo}$MHCII$^+$ cells can be subdivided according to their expression of FcγRII and/or FcγRIII and CD209a (Figures 6A and 6B). Both CD209a$^-$ and CD209a$^+$ FcγRII$^+$ and/or FcγRIII$^+$ populations rely on CCR2 (Figure S6D). Compared with CD4$^+$ DCs, CD209a$^-$ and CD209a$^+$ FcγRII$^+$ and/or FcγRIII$^+$ cells expressed greater CD115 and lower Flt3 and ESAM1 (Figures 6B and S6E). In stark contrast with CD4$^+$CD11b$^+$ DCs (Figures 6B and S6E), both expressed PD-L2 and low amounts of PD-L1 (Figures 6B and S6C). Additionally, both CD209a$^-$ and CD209a$^+$ FcγRII$^+$ and/or FcγRIII$^+$ cells had lower expression of YFP than CD4$^+$ DCs from naive or B16-GM-CSF-bearing *Zbtb46*^{Cre} x *Rosa*^{lslYFP} mice (Loschko et al., 2016; Figures 6B and S6F). A similar hierarchy of labeling was obtained in the *Zbtb46*$^{GFP/+}$ reporter mice (Satpathy et al., 2012;

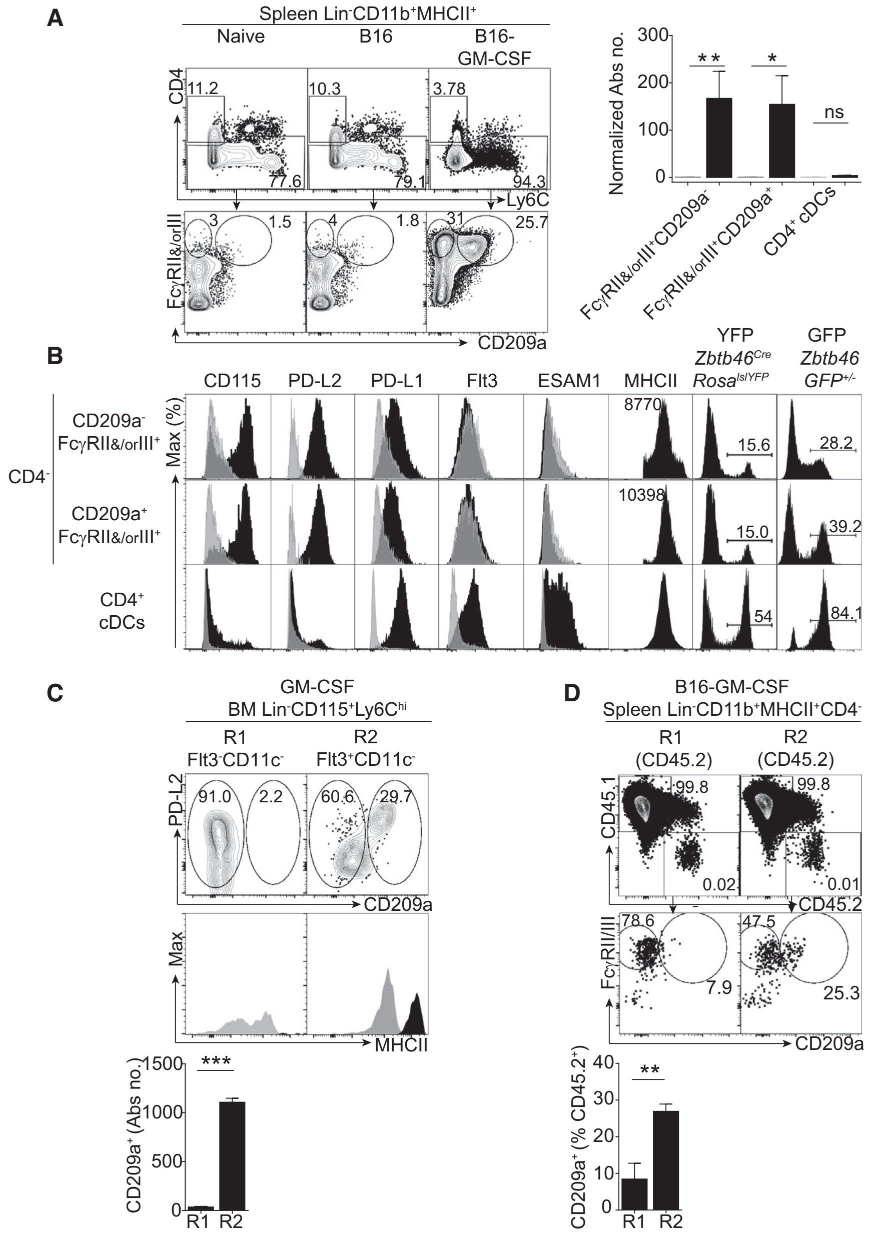

Figure 6. PU.1^{hi}Flt3^{+}MHCII^{+} R2 Monocytes Differentiate into PD-L2^{+}CD209a^{+} moDCs upon Exposure to GM-CSF

(A) Phenotype of spleen cells from control, B16, or B16-GM-CSF-engrafted mice. FACS analysis for CD4, Ly6C, FcγRII and/or FcγRIII, and CD209a expression within Lin^{-} (Ly6G^{-}, CD3ε^{-}, NK1.1^{-}, Ter119^{-}, CD45RA^{-}, cKit^{-}) CD11b^{+}MHCII^{+} spleen cells in naive, B16, or B16-GM-CSF-bearing mice. Absolute numbers of CD4^{-}FcγRII^{+} and/or FcγIII^{+}CD209a^{-}, CD4^{-}FcγRII^{+} and/or FcγIII^{+} CD209a^{+}, and CD4^{+} cells in Lin^{-}CD11b^{+}MHCII^{+} cells in spleens from B16-GM-CSF-engrafted mice. Data are normalized to the naive control of each population.

(B) Phenotype of CD4^{-}FcγRII^{+} and/or FcγIII^{+} CD209a^{-}, CD4^{-}FcγRII^{+} and/or FcγRIII^{+}CD209a^{+}, and CD4^{+} cells in Lin^{-}Ly6G^{-}CD11b^{+}MHCII^{+} splenocytes from B16-GM-CSF-bearing mice. Extracellular flow cytometry analysis of each population is shown for CD115, PDL2 (CD273), PDL1 (CD274), Flt3, ESAM1, and MHCII (black) against isotype controls (gray). YFP labeling of the indicated populations in *Zbtb46*^{Cre} *x Rosa*^{lslYFP} mice and GFP labeling in *Zbtb46*$^{GFP/+}$ mice is also shown. Numbers above the histograms indicate MFI. Data represent five mice over two experiments.

(C) GM-CSF culture of flow-cytometry-sorted R1 and R2 BM monocytes. Shown is PDL2 and CD209a expression on DAPI^{-}CD45^{+} cells after 2 days of culture and MHCII expression of CD209a^{-} (gray) and CD209a^{+} (black). Data represent three independent cultures.

(D) Cell fate of flow-cytometry-sorted R1 or R2 BM monocytes adoptively transferred into B16-GM-CSF-engrafted mice. Shown is FcγRII and/or FcγRIII and CD209a expression on recipient CD45.1^{+} (Figure S6G) and donor CD45.2^{+} cells in the spleens of CD45.1^{+} B16-GM-CSF-bearing recipients, as well as quantification of CD209a^{+} cells within Lin^{-}CD11b^{+}MHCII^{+}CD4^{-}CD45.2^{+} cells.

Data represent the mean ± SEM (*$p < 0.05$; **$p < 0.005$; ***$p < 0.0005$; ns, not significant; Student's t test). Please also refer to Figure S1.

Figure 6B). Together, these results support a monocytic origin of CD209a^{-} and CD209a^{+} FcγRII^{+} and/or FcγRIII^{+} cells.

Finally, we addressed whether R1 and/or R2 BM monocytes could differentiate into PDL2^{+}CD209a^{+} cells upon short-term culture in GM-CSF. We found that, reminiscent of macrophages produced during long-term GM-CSF culture (Barthélémy et al., 2015), R1 cells produced exclusively CD209a^{-}MHCII^{lo}PDL2^{+} cells. Unlike R1, R2 monocytes produced CD209a^{+}MHCII^{hi}PDL2^{+} cells (Figure 6C). In vivo, adoptive transfer of R2, but not R1, monocytes in B16-GM-CSF-engrafted mice generated CD209a^{+} moDCs, as was seen in the CD45.1^{+} recipients (Figures 6D and S6G). We conclude that R2, but not R1, monocytes exhibit a precursor ability for CD209a^{+}PDL2^{+} moDCs in vitro and in vivo.

An Increase in PU.1 Promotes the Generation of Monocyte-Derived PDL2^{+}CD209a^{+} moDCs

Finally, we investigated whether a higher expression of PU.1 is required for the differentiation of GM-CSF-induced moDCs. The generation of PD-L2^{hi}CD86^{hi}MHCII^{hi} moDCs (Barthélémy et al., 2015; Gao et al., 2013) from BM GM-CSF cultures was lower in *Sfpi1*$^{+/-}$ cultures than in WT cultures (Figure S7A). WT, but not *Sfpi1*$^{+/-}$ Ly6C^{hi}, monocytes generated PD-L2^{+}CD209a^{+} moDCs in vitro (Figure 7A). Analyzing B16-GM-CSF-engrafted *Sfpi1*$^{+/+}$ or *Sfpi1*$^{+/-}$ mice, we found that the expansion of CD209a^{+} moDCs (but not FcγRII^{+} and/or FcγRIII^{+} CD209a^{-} macrophages) was selectively decreased in *Sfpi1*$^{+/-}$ mice (Figure 7B).

We wondered whether PU.1 is required at the cell-autonomous level for the generation of CD209a^{+} moDCs. We adoptively

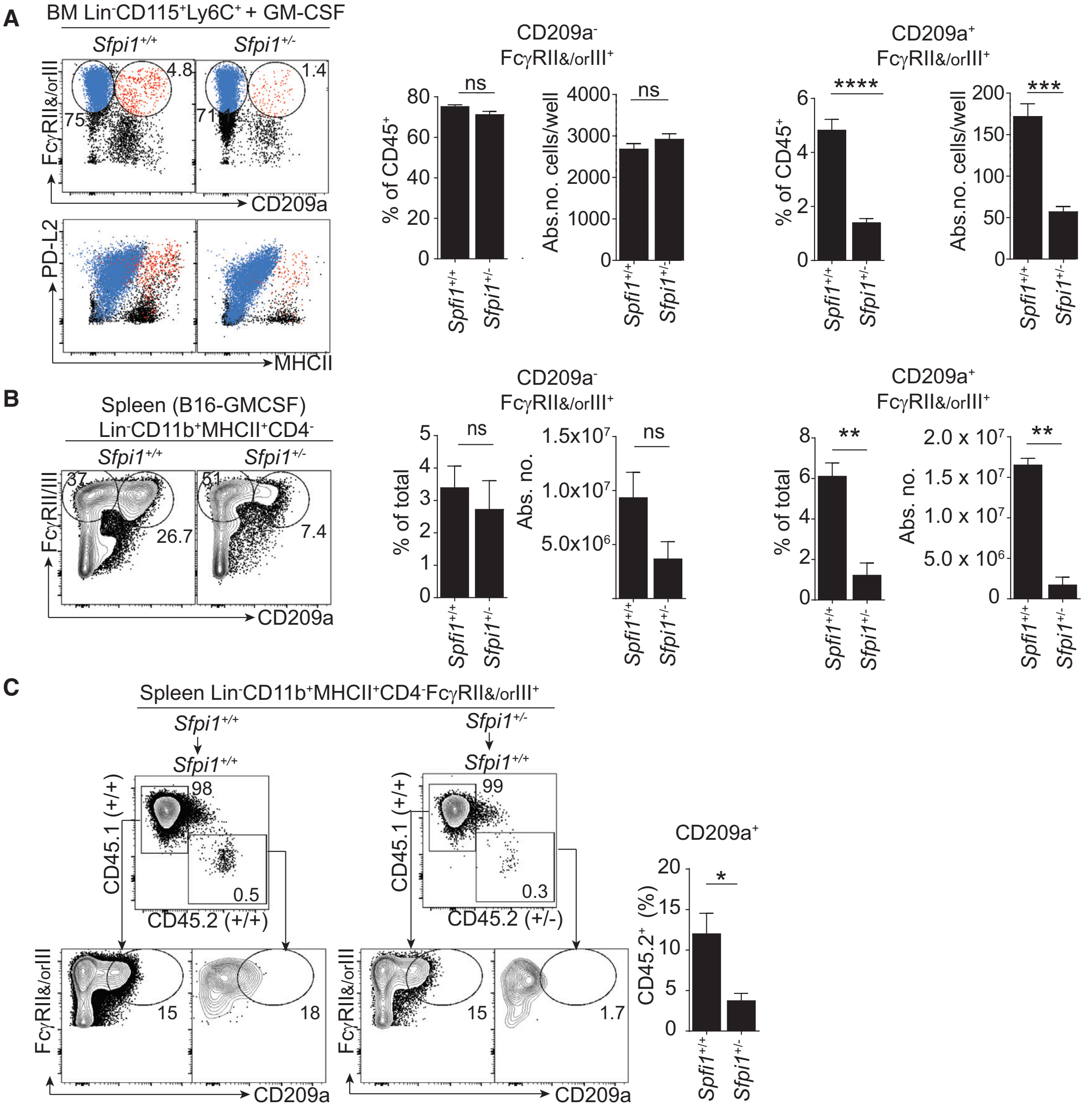

Figure 7. PU.1 Promotes the Generation of GM-CSF-Dependent PD-L2$^+$CD209a$^+$ moDCs

(A) FcγRII and/or FcγRIII and CD209a expression in GM-CSF culture of BM Lin$^-$CD115$^+$Ly6C$^+$ cells from *Sfpi1*$^{+/+}$ or *Sfpi1*$^{+/-}$ mice. FcγRII$^+$ and/or FcγRIII$^+$ CD209a$^-$ (blue) and FcγRII$^+$ and/or FcγRIII$^+$CD209a$^+$ (red) cells are overlaid on flow cytometry staining of PDL2 (CD273) and MHCII. The percentage and absolute number of DAPI$^-$CD45$^+$ cells of each indicated population of *Sfpi1*$^{+/+}$ or *Sfpi1*$^{+/-}$ BM are shown. Data represent two similar experiments.

(B) Quantification of FcγRII$^+$ and/or FcγRIII$^+$CD209a$^-$ and FcγRII$^+$ and/or FcγRIII$^+$CD209a$^+$ cells in Lin$^-$CD11b$^+$MHCII$^+$CD4$^-$ splenocytes from B16-GM-CSF-bearing *Sfpi1*$^{+/+}$or *Sfpi1*$^{+/-}$ mice. Data represent six mice.

(C) PU.1 promotes the generation of CD209a$^+$ moDCs by a cell-intrinsic mechanism. Shown is the adoptive transfer of CD45.2$^+$ *Sfpi1*$^{+/+}$ or *Sfpi1*$^{+/-}$ BM into B16-GM-CSF-engrafted CD45.1$^+$ congenic recipients, as well as CD209a and FcγRII and/or FcγRIII expression on Lin$^-$CD11b$^+$MHCII$^+$CD4$^-$ cells of recipient CD45.1$^+$ or donor CD45.2$^+$ *Sfpi1*$^{+/+}$ or *Sfpi1*$^{+/-}$ BM. The percentage of CD209a$^+$ cells inside the donor Lin$^-$CD11b$^+$MHCII$^+$CD4$^-$FcγRII$^+$ and/or FcγRIII$^+$CD45.2$^+$ cells is quantified (n = 3 mice per group in two identical experiments). Data represent the mean ± SEM (*$p < 0.05$, **$p < 0.005$, ***$p < 0.0005$; Student's t test). Please also refer to Figure S7.

transferred *Sfpi1*$^{+/+}$ or *Sfpi1*$^{+/-}$ CD45.2$^+$ BM into CD45.1$^+$ B16-GM-CSF-injected recipients. Unlike *Sfpi1*$^{+/+}$BM, *Sfpi1*$^{+/-}$ BM did not generate CD209a$^+$ moDCs (Figure 7C). Control populations such as B cells and granulocytes were generated as efficiently upon transfer of either *Sfpi1*$^{+/+}$ or *Sfpi1*$^{+/-}$ BM (Figure S7B). We conclude that PU.1 selectively controls the differentiation of GM-CSF-dependent CD209a$^+$ moDCs by a cell-intrinsic mechanism.

DISCUSSION

Depending on the study, monocyte-derived inflammatory cells (distinct from the cDC lineage) are termed inflammatory macrophages (Bain et al., 2013; Tamoutounour et al., 2012) or moDCs (Cheong et al., 2010; Plantinga et al., 2013; Zigmond et al., 2012). Here, we report that distinct monocyte subsets give rise to iNOS$^+$ inflammatory macrophages and CD209a$^+$ moDCs.

Indeed, we have shown that a subset of monocytes (R2; CCR2 dependent and not labeled in *Zbtb46^{Cre} x Rosa^{lslYFP}* mice) contain MHCII$^+$ cells that are distinct from BTBD4$^+$ pre-DCs. These cells develop into CD209a$^+$ moDCs upon exposure to GM-CSF both in vitro and in vivo. Conversely, MHCII$^-$ monocytes (R1) possess the progenitor function for iNOS$^+$ inflammatory macrophages. This supports the view that inflammatory macrophages and moDCs are ontogenically distinct populations. In support of this hypothesis, fate mapping of iNOS$^+$ inflammatory macrophages did not show an efficient conversion to the moDC phenotype. In addition, single-cell analysis of monocytes (R2; CD11c$^-$) identified a mixed transcriptional program characterized by the expression of MHCII genes (downstream activation of CIITA by pI promoter) and DC-related genes such as *Flt3*, *Cd209a*, or *Kmo*, for example. These cells might originate from cMoPs (through Flt3 acquisition), from CDPs (via loss of DC-specific commitment), or from earlier myeloid-primed progenitors (LMPP or GMPs). Additional experiments are needed to address this. Of note, we have shown that R3 cells (CD11c$^+$Flt3$^+$CD115$^+$BTBD4$^+$SiglecH$^-$Ly6C$^+$) aligning with previously described cDC2-commited pre-cDCs (pre-DC2s) (Schlitzer et al., 2015; Tussiwand et al., 2015) are diverse in terms of MHCII or CD209a expression, for example. The functional relevance of pre-DC2 heterogeneity for the generation of multiple cDC2 subsets (Lewis et al., 2011; Tussiwand et al., 2015) remains to be addressed.

An important question that arises is the transcriptional mechanism driving the steady-state differentiation of Ly6C$^+$ monocytes into a small sub-population with a distinct potential to generate moDCs or inflammatory macrophages. PU.1 is a lineage-determining TF essential for hematopoietic stem cells and has multiple roles in the myeloid lineage (Dakic et al., 2005; DeKoter and Singh, 2000; McKercher et al., 1996; Scott et al., 1994). PU.1 cooperates with multiple other TFs to shape the enhancer landscape of tissue-resident macrophages (Lavin et al., 2014; Norris et al., 2014). Here, we report that *Sfpi1* haploinsufficiency promotes the generation of iNOS$^+$ macrophages during *L.m.* infection. PU.1-dependent negative regulation of iNOS$^+$ macrophages might constitute a regulatory mechanism limiting iNOS-dependent immunopathology. How does PU.1 downregulate the production of iNOS? PU.1 is known to upregulate multiple miRNAs, including miR-146 and miR-155, which in turn negatively regulate innate sensing through the regulation of TRAF6, IRAK4, and STAT1, for example (Ghani et al., 2011; Jurkin et al., 2010). Indeed, Ly6C$^+$ monocytes from *mir146$^{-/-}$* mice are hyper-responsive to microbial stimulation (Etzrodt et al., 2012). Higher amounts of PU.1 might be needed to induce mir146 and limit anti-microbial responses. Further experiments are needed to assess the relevance of miRNAs downstream of PU.1-dependent regulation of innate sensing.

We have found that, in addition to having a regulatory effect on microbicidal iNOS$^+$ macrophages, the highest amounts of PU.1 selectively promote the generation of GM-CSF-dependent moDCs in vitro and in vivo. This could be explained by (1) the reduction of moDC precursors (MHCII$^+$ R2 monocytes) in naive *Sfpi1$^{+/-}$* mice and (2) an effect on moDC terminal differentiation. In support of the latter, overexpression of PU.1 promotes the differentiation of DC-like cells (Bakri et al., 2005), and inducible ablation of *Sfpi1* prevents the differentiation of DCs (Carotta et al., 2010). In this context, PU.1 cooperation with TFs IRF4 and IRF8 could be relevant to explain the role of PU.1 in moDC differentiation from monocytes. PU.1 can bind to Ets binding sites on its own, but PU.1 also cooperates with IRF4 or IRF8 at Ets-IRF composite response elements called EICEs (Brass et al., 1999). IRF4 is known to be involved in the control of CIITA promoter pI in GM-CSF moDCs or CD11b$^+$ DCs (Gao et al., 2013; Tamura et al., 2005; Vander Lugt et al., 2014). In addition, PU.1 might boost the expression of growth factor receptors required for the development of moDCs (e.g., CSFR2A; DeKoter et al., 1998).

In conclusion, our results shed light on the readiness of inflammatory monocyte subsets for distinct and specialized developmental programs activated in inflammatory conditions. Importantly, PU.1 amounts segregate the transcriptional programs of microbicidal iNOS$^+$ macrophages or moDCs.

EXPERIMENTAL PROCEDURES

Mice

All mice used were between 6 and 12 weeks old and were matched for age and sex in all experiments. They were maintained under specific-pathogen-free conditions in accordance with the UK Animals (Scientific Procedures) Act of 1986.

Cell Isolation and Fluorescence-Activated Cell Sorting

For preparation of BM cell suspensions, the bones of both hind limbs (two tibia and two femurs) were flushed with ice-cold fluorescence-activated cell sorting (FACS) buffer (PBS (Life technologies) with 1% BSA (Apollo Scientific Ltd) and 2 mM EDTA (Life Technologies). Spleens were collected, cut into small pieces, and incubated with collagenase D (Roche) and DNaseI (Roche) in Hank's balanced salt solution (GE Healthcare) and 5% fetal bovine serum (Life technologies) for 20 min; they were further macerated through 100 μm cell strainers (BD Falcon). Red blood cells were lysed with 2 ml of Ack lysis buffer (Life Technologies), incubated for 2 min at room temperature, and then diluted with FACS buffer. After centrifugation, cells were either re-suspended in an antibody cocktail in FACS buffer or permeabilized and fixed for intracellular staining and analyzed by flow cytometry with FlowJo software (TreeStar). For cell sorting, see Supplemental Experimental Procedures.

In Vitro GM-CSF Cultures

Total BM or 10^4 sorted Ly6C^{hi}CD115$^+$ monocytes (total or subsets R1–R3) were cultured in 20 ng/ml of GM-CSF in complete RPMI with 6,000 live MS-5 cells as "feeders," which were plated on the same day. Analyzed cells were pre-gated to be DAPI$^-$ and CD45$^+$.

In Vitro *L.m.* Infections

Primary cells infected with *L.m.* were in vivo cultured overnight at an MOI of 0.01, 0.1, 1, or 10 (as indicated in Figure 5) in complete RPMI medium supplemented with macrophage colony-stimulating factor (MCSF, 20 ng/ml; Peprotech), GM-CSF (3 ng/ml; Peprotech), and human Flt3L (100 ng/ml; CellDex). BMDMs were derived by culture of whole BM in RPMI supplemented with culture medium from L-929 cells.

B16-GM-CSF Tumor Experiments

B16-GM-CSF tumor cells were checked for viability with Trypan Blue, and 1.5×10^5 to 3×10^5 live cells were injected subcutaneously (see Supplemental Experimental Procedures).

Infection

4×10^3 to 5×10^3 WT colony-forming units (CFU) (*Listeria*) or 10^6 ΔActA mutant CFU of *L.m.* (*ΔActA Listeria*) were injected intravenously into sterile PBS (see Supplemental Experimental Procedures).

Microarray

Cells sorted by flow cytometry were collected in complete RPMI and pelleted, lysed in Buffer RLT (RNeasy Kit, QIAGEN), and frozen at $-80^{\circ}C$ until they were processed for RNA. The NuGEN Ovation Pico WTA V2 Kit was used to process 1 ng RNA per sample into cDNA amplified by single-primer isothermal amplification. The Encore Biotin Module (NuGEN) was used to fragment and label the cDNA with biotin. Hybridization cocktails were prepared as recommended by NuGEN and hybridized to Affymetrix Mouse Gene 1.0 ST arrays overnight. Arrays were washed and stained with Affymetrix Fluidics Station FS450 and the GeneChip Hybridization, Wash, and Stain Kit and scanned by the GeneChip Scanner 3000 7G with Autoloader. Raw data files (DAT and CEL) were generated in Affymetrix GeneChip Command Console software and are available at GEO: GSE90471. Data were analyzed with GenePattern software (Broad Institute), and pre-DC and CDP datasets were obtained from public databases.

t-SNE Analysis

Single-cell analysis using the t-SNE algorithm was done on flow cytometry data in the online platform provided by Cytobank (see Supplemental Experimental Procedures).

Single-Cell qPCR

Single cells were sorted by flow cytometry, cDNA was amplified with the CellsDirect One-Step qRT-PCR Kit (ThermoFisher), and qPCR was run on a BioMark HD (Fluidigm) with the help of Taqman probes (Life Technologies) for the genes indicated in Figure 2. The 45 targeted genes were analyzed against an average of three housekeeping genes: *Hprt*, *ActB*, and *Gapdh*. Analysis was done with the help of Gene-E software (Broad Institute) (see Supplemental Experimental Procedures).

qPCR

qPCR was carried out in duplicate for samples sorted from three independent sorting experiments. Primers used for testing *Zbtb46* were 5′-AGAGAG CACATGAAGCGACA-3′ (forward) and 5′-CTGGCTGCAGACATGAACAC-3′ (reverse). Results were normalized against β-actin: 5′-ATGCTCCCCGGG CTGTAT-3′ (forward) and 5′-CATAGGAGTCCTTCTGACCCATTC-3′ (reverse).

Adoptive Transfer

For adoptive transfer of cells sorted by flow cytometry, 3.3×10^5 cells of each population were collected in complete RPMI, centrifuged, resuspended in 120 μl of RPMI alone, and injected intravenously into each $CD45.1^+$ congenic recipient. For whole-BM transfers, single-cell suspensions of BM were depleted of red blood cells by exposure to RBC lysis buffer (Life technologies) and counted. 40×10^6 whole-BM cells from $Sfpi1^{+/+}$ or $Sfpi1^{+/-}$ mice were transferred into B16-GM-CSF-treated congenic $CD45.1^+$ mice on day 9 and analyzed on day 11 after tumor injection. For *L.m.*-infected recipients, 20×10^6 whole-BM cells were transferred into congenic $CD45.1^+$ recipients 2 hr before intravenous *L.m.* infection.

Statistical Analysis

Data were analyzed for statistical significance by unpaired Student's t tests. Differences were considered significant at $p < 0.05$ (*$p < 0.05$; **$p < 0.005$; ***$p < 0.0005$; ****$p < 0.00005$; ns, non-significant).

ACCESSION NUMBERS

The accession number for the microarray data reported in this paper is GEO: GSE90471.

SUPPLEMENTAL INFORMATION

Supplemental Information includes Supplemental Experimental Procedures, seven figures, and one table and can be found with this article online at http://dx.doi.org/10.1016/j.immuni.2016.12.001.

AUTHOR CONTRIBUTIONS

S.M. performed most of the experiments (with help from R.P., D.M., and G.A. for some) and designed the figures. T.P. performed the single-cell multiplex qPCR; R.G. provided expertise for the design of these experiments. J.D.A., S.H., and A.-M.L.-D. provided help for the analysis of MHCII expression and its transcriptional regulation and performed some experiments addressing the role of CIITA. J.L., M.C.N., and M.P.L. provided tools and expertise for the analysis of *Zbtb46* expression and the fate mapping of *Zbtb46*-expressing cells. G.L., A.B., and F.G. provided reagents and expertise with the fate mapping of iNOS-expressing cells. J.Y.H and S.A.Q provided reagents and expertise with the B16-GM-CSF melanoma model. E.L.G, E.G.-M, C.E.J.-G., E.G.-P., H.G., and F.G. provided mice models, expertise for the analysis of monocyte populations, and helpful discussions. The manuscript was written by P.G. and S.M. Experiments were designed by P.G. in collaboration with S.M.

ACKNOWLEDGMENTS

P.G. is a CNRS investigator. S.M. is funded by King's Overseas Research Studentships. The research was supported by the MRC (MR/K01241X/1), BBSRC (BB/M029735/1), and King's Health Partners. We thank Dr. Loredana Saveanu, Prof. Caetano Reis e Sousa, and Dr. Julie Helft for reagents and insightful discussions. All flow cytometry work was performed within the NIHR Biomedical Research Centre based at Guy's and St. Thomas' NHS Foundation Trust and King's College London; we especially thank Prabhjoat Singh Chana for help at the facility and Nedyalko Petrov for assistance with t-SNE analysis. We would like to thank Matthew Arno and Erick Nasser at the Genomics Centre of King's College London for performing the microarray experiments and providing technical support. The views expressed are those of the authors and not necessarily those of the NHS, the NIHR, or the Department of Health.

Received: September 28, 2015
Revised: June 30, 2016
Accepted: October 24, 2016
Published: December 20, 2016

REFERENCES

Auffray, C., Fogg, D., Garfa, M., Elain, G., Join-Lambert, O., Kayal, S., Sarnacki, S., Cumano, A., Lauvau, G., and Geissmann, F. (2007). Monitoring of blood vessels and tissues by a population of monocytes with patrolling behavior. Science *317*, 666–670.

Bain, C.C., Scott, C.L., Uronen-Hansson, H., Gudjonsson, S., Jansson, O., Grip, O., Guilliams, M., Malissen, B., Agace, W.W., and Mowat, A.M. (2013). Resident and pro-inflammatory macrophages in the colon represent alternative context-dependent fates of the same Ly6Chi monocyte precursors. Mucosal Immunol. *6*, 498–510.

Bakri, Y., Sarrazin, S., Mayer, U.P., Tillmanns, S., Nerlov, C., Boned, A., and Sieweke, M.H. (2005). Balance of MafB and PU.1 specifies alternative macrophage or dendritic cell fate. Blood *105*, 2707–2716.

Barthélémy, O., Degrell, P., Berman, E., Kerneis, M., Petroni, T., Silvain, J., Payot, L., Choussat, R., Collet, J.P., Helft, G., et al. (2015). Sex-related differences after contemporary primary percutaneous coronary intervention for ST-segment elevation myocardial infarction. Arch. Cardiovasc. Dis. *108*, 428–436.

Brass, A.L., Zhu, A.Q., and Singh, H. (1999). Assembly requirements of PU.1-Pip (IRF-4) activator complexes: inhibiting function in vivo using fused dimers. EMBO J. *18*, 977–991.

Breton, G., Lee, J., Zhou, Y.J., Schreiber, J.J., Keler, T., Puhr, S., Anandasabapathy, N., Schlesinger, S., Caskey, M., Liu, K., and Nussenzweig, M.C. (2015). Circulating precursors of human CD1c+ and CD141+ dendritic cells. J. Exp. Med. *212*, 401–413.

Carlin, L.M., Stamatiades, E.G., Auffray, C., Hanna, R.N., Glover, L., Vizcay-Barrena, G., Hedrick, C.C., Cook, H.T., Diebold, S., and Geissmann, F. (2013). Nr4a1-dependent Ly6C(low) monocytes monitor endothelial cells and orchestrate their disposal. Cell *153*, 362–375.

Carotta, S., Dakic, A., D'Amico, A., Pang, S.H., Greig, K.T., Nutt, S.L., and Wu, L. (2010). The transcription factor PU.1 controls dendritic cell development and Flt3 cytokine receptor expression in a dose-dependent manner. Immunity *32*, 628–641.

Cheong, C., Matos, I., Choi, J.H., Dandamudi, D.B., Shrestha, E., Longhi, M.P., Jeffrey, K.L., Anthony, R.M., Kluger, C., Nchinda, G., et al. (2010). Microbial stimulation fully differentiates monocytes to DC-SIGN/CD209(+) dendritic cells for immune T cell areas. Cell *143*, 416–429.

Dakic, A., Metcalf, D., Di Rago, L., Mifsud, S., Wu, L., and Nutt, S.L. (2005). PU.1 regulates the commitment of adult hematopoietic progenitors and restricts granulopoiesis. J. Exp. Med. *201*, 1487–1502.

Daro, E., Pulendran, B., Brasel, K., Teepe, M., Pettit, D., Lynch, D.H., Vremec, D., Robb, L., Shortman, K., McKenna, H.J., et al. (2000). Polyethylene glycol-modified GM-CSF expands CD11b(high)CD11c(high) but notCD11b(low) CD11c(high) murine dendritic cells in vivo: a comparative analysis with Flt3 ligand. J. Immunol. *165*, 49–58.

DeKoter, R.P., and Singh, H. (2000). Regulation of B lymphocyte and macrophage development by graded expression of PU.1. Science *288*, 1439–1441.

DeKoter, R.P., Walsh, J.C., and Singh, H. (1998). PU.1 regulates both cytokine-dependent proliferation and differentiation of granulocyte/macrophage progenitors. EMBO J. *17*, 4456–4468.

Dranoff, G., Jaffee, E., Lazenby, A., Golumbek, P., Levitsky, H., Brose, K., Jackson, V., Hamada, H., Pardoll, D., and Mulligan, R.C. (1993). Vaccination with irradiated tumor cells engineered to secrete murine granulocyte-macrophage colony-stimulating factor stimulates potent, specific, and long-lasting anti-tumor immunity. Proc. Natl. Acad. Sci. USA *90*, 3539–3543.

Etzrodt, M., Cortez-Retamozo, V., Newton, A., Zhao, J., Ng, A., Wildgruber, M., Romero, P., Wurdinger, T., Xavier, R., Geissmann, F., et al. (2012). Regulation of monocyte functional heterogeneity by miR-146a and Relb. Cell Rep. *1*, 317–324.

Fogg, D.K., Sibon, C., Miled, C., Jung, S., Aucouturier, P., Littman, D.R., Cumano, A., and Geissmann, F. (2006). A clonogenic bone marrow progenitor specific for macrophages and dendritic cells. Science *311*, 83–87.

Gao, Y., Nish, S.A., Jiang, R., Hou, L., Licona-Limón, P., Weinstein, J.S., Zhao, H., and Medzhitov, R. (2013). Control of T helper 2 responses by transcription factor IRF4-dependent dendritic cells. Immunity *39*, 722–732.

Gautier, E.L., Shay, T., Miller, J., Greter, M., Jakubzick, C., Ivanov, S., Helft, J., Chow, A., Elpek, K.G., Gordonov, S., et al.; Immunological Genome Consortium (2012). Gene-expression profiles and transcriptional regulatory pathways that underlie the identity and diversity of mouse tissue macrophages. Nat. Immunol. *13*, 1118–1128.

Geissmann, F., Jung, S., and Littman, D.R. (2003). Blood monocytes consist of two principal subsets with distinct migratory properties. Immunity *19*, 71–82.

Ghani, S., Riemke, P., Schönheit, J., Lenze, D., Stumm, J., Hoogenkamp, M., Lagendijk, A., Heinz, S., Bonifer, C., Bakkers, J., et al. (2011). Macrophage development from HSCs requires PU.1-coordinated microRNA expression. Blood *118*, 2275–2284.

Hanna, R.N., Shaked, I., Hubbeling, H.G., Punt, J.A., Wu, R., Herrley, E., Zaugg, C., Pei, H., Geissmann, F., Ley, K., and Hedrick, C.C. (2012). NR4A1 (Nur77) deletion polarizes macrophages toward an inflammatory phenotype and increases atherosclerosis. Circ. Res. *110*, 416–427.

Hettinger, J., Richards, D.M., Hansson, J., Barra, M.M., Joschko, A.C., Krijgsveld, J., and Feuerer, M. (2013). Origin of monocytes and macrophages in a committed progenitor. Nat. Immunol. *14*, 821–830.

Jakubzick, C., Gautier, E.L., Gibbings, S.L., Sojka, D.K., Schlitzer, A., Johnson, T.E., Ivanov, S., Duan, Q., Bala, S., Condon, T., et al. (2013). Minimal differentiation of classical monocytes as they survey steady-state tissues and transport antigen to lymph nodes. Immunity *39*, 599–610.

Jurkin, J., Schichl, Y.M., Koeffel, R., Bauer, T., Richter, S., Konradi, S., Gesslbauer, B., and Strobl, H. (2010). miR-146a is differentially expressed by myeloid dendritic cell subsets and desensitizes cells to TLR2-dependent activation. J. Immunol. *184*, 4955–4965.

Kurotaki, D., Osato, N., Nishiyama, A., Yamamoto, M., Ban, T., Sato, H., Nakabayashi, J., Umehara, M., Miyake, N., Matsumoto, N., et al. (2013). Essential role of the IRF8-KLF4 transcription factor cascade in murine monocyte differentiation. Blood *121*, 1839–1849.

Langlet, C., Tamoutounour, S., Henri, S., Luche, H., Ardouin, L., Grégoire, C., Malissen, B., and Guilliams, M. (2012). CD64 expression distinguishes monocyte-derived and conventional dendritic cells and reveals their distinct role during intramuscular immunization. J. Immunol. *188*, 1751–1760.

Lavin, Y., Winter, D., Blecher-Gonen, R., David, E., Keren-Shaul, H., Merad, M., Jung, S., and Amit, I. (2014). Tissue-resident macrophage enhancer landscapes are shaped by the local microenvironment. Cell *159*, 1312–1326.

Lee, J., Breton, G., Oliveira, T.Y., Zhou, Y.J., Aljoufi, A., Puhr, S., Cameron, M.J., Sékaly, R.P., Nussenzweig, M.C., and Liu, K. (2015). Restricted dendritic cell and monocyte progenitors in human cord blood and bone marrow. J. Exp. Med. *212*, 385–399.

LeibundGut-Landmann, S., Waldburger, J.M., Reis e Sousa, C., Acha-Orbea, H., and Reith, W. (2004). MHC class II expression is differentially regulated in plasmacytoid and conventional dendritic cells. Nat. Immunol. *5*, 899–908.

Lewis, K.L., Caton, M.L., Bogunovic, M., Greter, M., Grajkowska, L.T., Ng, D., Klinakis, A., Charo, I.F., Jung, S., Gommerman, J.L., et al. (2011). Notch2 receptor signaling controls functional differentiation of dendritic cells in the spleen and intestine. Immunity *35*, 780–791.

Liu, K., Victora, G.D., Schwickert, T.A., Guermonprez, P., Meredith, M.M., Yao, K., Chu, F.F., Randolph, G.J., Rudensky, A.Y., and Nussenzweig, M. (2009). In vivo analysis of dendritic cell development and homeostasis. Science *324*, 392–397.

Loschko, J., Schreiber, H.A., Rieke, G.J., Esterházy, D., Meredith, M.M., Pedicord, V.A., Yao, K.H., Caballero, S., Pamer, E.G., Mucida, D., and Nussenzweig, M.C. (2016). Absence of MHC class II on cDCs results in microbial-dependent intestinal inflammation. J. Exp. Med. *213*, 517–534.

Mach, N., Gillessen, S., Wilson, S.B., Sheehan, C., Mihm, M., and Dranoff, G. (2000). Differences in dendritic cells stimulated in vivo by tumors engineered to secrete granulocyte-macrophage colony-stimulating factor or Flt3-ligand. Cancer Res. *60*, 3239–3246.

MacMicking, J.D., Nathan, C., Hom, G., Chartrain, N., Fletcher, D.S., Trumbauer, M., Stevens, K., Xie, Q.W., Sokol, K., Hutchinson, N., et al. (1995). Altered responses to bacterial infection and endotoxic shock in mice lacking inducible nitric oxide synthase. Cell *81*, 641–650.

McKenna, H.J., Stocking, K.L., Miller, R.E., Brasel, K., De Smedt, T., Maraskovsky, E., Maliszewski, C.R., Lynch, D.H., Smith, J., Pulendran, B., et al. (2000). Mice lacking flt3 ligand have deficient hematopoiesis affecting hematopoietic progenitor cells, dendritic cells, and natural killer cells. Blood *95*, 3489–3497.

McKercher, S.R., Torbett, B.E., Anderson, K.L., Henkel, G.W., Vestal, D.J., Baribault, H., Klemsz, M., Feeney, A.J., Wu, G.E., Paige, C.J., and Maki, R.A. (1996). Targeted disruption of the PU.1 gene results in multiple hematopoietic abnormalities. EMBO J. *15*, 5647–5658.

Meredith, M.M., Liu, K., Darrasse-Jeze, G., Kamphorst, A.O., Schreiber, H.A., Guermonprez, P., Idoyaga, J., Cheong, C., Yao, K.H., Niec, R.E., and Nussenzweig, M.C. (2012). Expression of the zinc finger transcription factor zDC (Zbtb46, Btbd4) defines the classical dendritic cell lineage. J. Exp. Med. *209*, 1153–1165.

Miller, J.C., Brown, B.D., Shay, T., Gautier, E.L., Jojic, V., Cohain, A., Pandey, G., Leboeuf, M., Elpek, K.G., Helft, J., et al.; Immunological Genome Consortium (2012). Deciphering the transcriptional network of the dendritic cell lineage. Nat. Immunol. *13*, 888–899.

Naik, S.H., Sathe, P., Park, H.Y., Metcalf, D., Proietto, A.I., Dakic, A., Carotta, S., O'Keeffe, M., Bahlo, M., Papenfuss, A., et al. (2007). Development of plasmacytoid and conventional dendritic cell subtypes from single precursor cells derived in vitro and in vivo. Nat. Immunol. *8*, 1217–1226.

Naik, S.H., Perié, L., Swart, E., Gerlach, C., van Rooij, N., de Boer, R.J., and Schumacher, T.N. (2013). Diverse and heritable lineage imprinting of early haematopoietic progenitors. Nature *496*, 229–232.

Norris, P.C., Gosselin, D., Reichart, D., Glass, C.K., and Dennis, E.A. (2014). Phospholipase A2 regulates eicosanoid class switching during inflammasome activation. Proc. Natl. Acad. Sci. USA *111*, 12746–12751.

Nussenzweig, M.C., Steinman, R.M., Gutchinov, B., and Cohn, Z.A. (1980). Dendritic cells are accessory cells for the development of anti-trinitrophenyl cytotoxic T lymphocytes. J. Exp. Med. *152*, 1070–1084.

Onai, N., Obata-Onai, A., Schmid, M.A., Ohteki, T., Jarrossay, D., and Manz, M.G. (2007). Identification of clonogenic common Flt3+M-CSFR+ plasmacytoid and conventional dendritic cell progenitors in mouse bone marrow. Nat. Immunol. *8*, 1207–1216.

Plantinga, M., Guilliams, M., Vanheerswynghels, M., Deswarte, K., Branco-Madeira, F., Toussaint, W., Vanhoutte, L., Neyt, K., Killeen, N., Malissen, B., et al. (2013). Conventional and monocyte-derived CD11b(+) dendritic cells initiate and maintain T helper 2 cell-mediated immunity to house dust mite allergen. Immunity *38*, 322–335.

Sathe, P., Metcalf, D., Vremec, D., Naik, S.H., Langdon, W.Y., Huntington, N.D., Wu, L., and Shortman, K. (2014). Lymphoid tissue and plasmacytoid dendritic cells and macrophages do not share a common macrophage-dendritic cell-restricted progenitor. Immunity *41*, 104–115.

Satpathy, A.T., Kc, W., Albring, J.C., Edelson, B.T., Kretzer, N.M., Bhattacharya, D., Murphy, T.L., and Murphy, K.M. (2012). Zbtb46 expression distinguishes classical dendritic cells and their committed progenitors from other immune lineages. J. Exp. Med. *209*, 1135–1152.

Schlitzer, A., Sivakamasundari, V., Chen, J., Sumatoh, H.R., Schreuder, J., Lum, J., Malleret, B., Zhang, S., Larbi, A., Zolezzi, F., et al. (2015). Identification of cDC1- and cDC2-committed DC progenitors reveals early lineage priming at the common DC progenitor stage in the bone marrow. Nat. Immunol. *16*, 718–728.

Schraml, B.U., van Blijswijk, J., Zelenay, S., Whitney, P.G., Filby, A., Acton, S.E., Rogers, N.C., Moncaut, N., Carvajal, J.J., and Reis e Sousa, C. (2013). Genetic tracing via DNGR-1 expression history defines dendritic cells as a hematopoietic lineage. Cell *154*, 843–858.

Schreiber, H.A., Loschko, J., Karssemeijer, R.A., Escolano, A., Meredith, M.M., Mucida, D., Guermonprez, P., and Nussenzweig, M.C. (2013). Intestinal monocytes and macrophages are required for T cell polarization in response to Citrobacter rodentium. J. Exp. Med. *210*, 2025–2039.

Scott, E.W., Simon, M.C., Anastasi, J., and Singh, H. (1994). Requirement of transcription factor PU.1 in the development of multiple hematopoietic lineages. Science *265*, 1573–1577.

Serbina, N.V., and Pamer, E.G. (2006). Monocyte emigration from bone marrow during bacterial infection requires signals mediated by chemokine receptor CCR2. Nat. Immunol. *7*, 311–317.

Serbina, N.V., Salazar-Mather, T.P., Biron, C.A., Kuziel, W.A., and Pamer, E.G. (2003). TNF/iNOS-producing dendritic cells mediate innate immune defense against bacterial infection. Immunity *19*, 59–70.

Steinman, R.M., and Cohn, Z.A. (1973). Identification of a novel cell type in peripheral lymphoid organs of mice. I. Morphology, quantitation, tissue distribution. J. Exp. Med. *137*, 1142–1162.

Sunderkötter, C., Nikolic, T., Dillon, M.J., Van Rooijen, N., Stehling, M., Drevets, D.A., and Leenen, P.J. (2004). Subpopulations of mouse blood monocytes differ in maturation stage and inflammatory response. J. Immunol. *172*, 4410–4417.

Tamoutounour, S., Henri, S., Lelouard, H., de Bovis, B., de Haar, C., van der Woude, C.J., Woltman, A.M., Reyal, Y., Bonnet, D., Sichien, D., et al. (2012). CD64 distinguishes macrophages from dendritic cells in the gut and reveals the Th1-inducing role of mesenteric lymph node macrophages during colitis. Eur. J. Immunol. *42*, 3150–3166.

Tamura, T., Tailor, P., Yamaoka, K., Kong, H.J., Tsujimura, H., O'Shea, J.J., Singh, H., and Ozato, K. (2005). IFN regulatory factor-4 and -8 govern dendritic cell subset development and their functional diversity. J. Immunol. *174*, 2573–2581.

Tussiwand, R., Everts, B., Grajales-Reyes, G.E., Kretzer, N.M., Iwata, A., Bagaitkar, J., Wu, X., Wong, R., Anderson, D.A., Murphy, T.L., et al. (2015). Klf4 expression in conventional dendritic cells is required for T helper 2 cell responses. Immunity *42*, 916–928.

Vander Lugt, B., Khan, A.A., Hackney, J.A., Agrawal, S., Lesch, J., Zhou, M., Lee, W.P., Park, S., Xu, M., DeVoss, J., et al. (2014). Transcriptional programming of dendritic cells for enhanced MHC class II antigen presentation. Nat. Immunol. *15*, 161–167.

Varol, C., Landsman, L., Fogg, D.K., Greenshtein, L., Gildor, B., Margalit, R., Kalchenko, V., Geissmann, F., and Jung, S. (2007). Monocytes give rise to mucosal, but not splenic, conventional dendritic cells. J. Exp. Med. *204*, 171–180.

Waskow, C., Liu, K., Darrasse-Jèze, G., Guermonprez, P., Ginhoux, F., Merad, M., Shengelia, T., Yao, K., and Nussenzweig, M. (2008). The receptor tyrosine kinase Flt3 is required for dendritic cell development in peripheral lymphoid tissues. Nat. Immunol. *9*, 676–683.

Yona, S., Kim, K.W., Wolf, Y., Mildner, A., Varol, D., Breker, M., Strauss-Ayali, D., Viukov, S., Guilliams, M., Misharin, A., et al. (2013). Fate mapping reveals origins and dynamics of monocytes and tissue macrophages under homeostasis. Immunity *38*, 79–91.

Zigmond, E., Varol, C., Farache, J., Elmaliah, E., Satpathy, A.T., Friedlander, G., Mack, M., Shpigel, N., Boneca, I.G., Murphy, K.M., et al. (2012). Ly6C hi monocytes in the inflamed colon give rise to proinflammatory effector cells and migratory antigen-presenting cells. Immunity *37*, 1076–1090.

Immunity, Volume *45*

Supplemental Information

The Heterogeneity of Ly6C^{hi} Monocytes Controls Their Differentiation into iNOS^{+} Macrophages or Monocyte-Derived Dendritic Cells

Shinelle Menezes, Daisy Melandri, Giorgio Anselmi, Thibaut Perchet, Jakob Loschko, Juan Dubrot, Rajen Patel, Emmanuel L. Gautier, Stéphanie Hugues, M. Paula Longhi, Jake Y. Henry, Sergio A. Quezada, Grégoire Lauvau, Ana-Maria Lennon-Duménil, Enrique Gutiérrez-Martínez, Alain Bessis, Elisa Gomez-Perdiguero, Christian E. Jacome-Galarza, Hannah Garner, Frederic Geissmann, Rachel Golub, Michel C. Nussenzweig, and Pierre Guermonprez

FIGURE S1

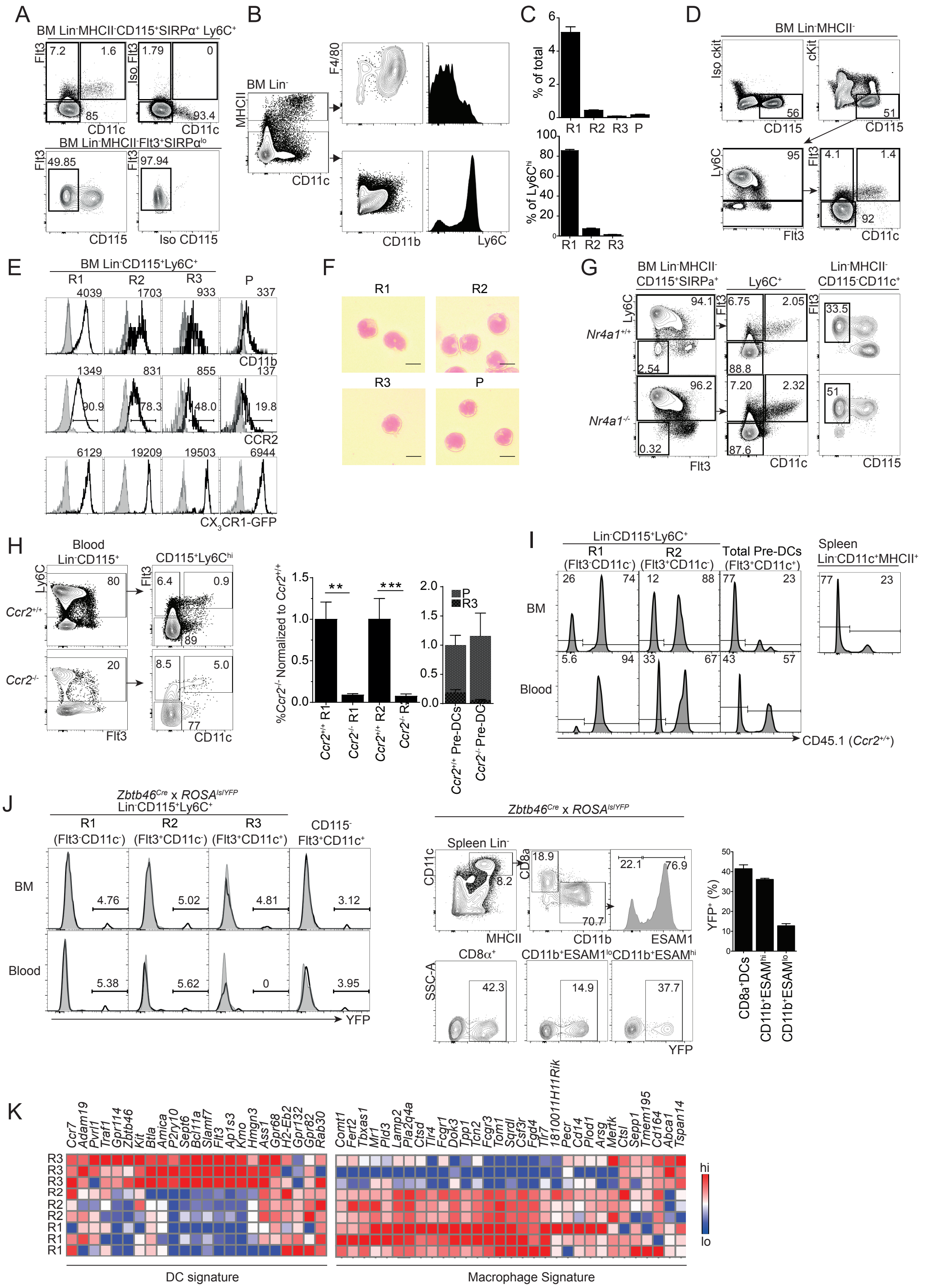

A
BM Lin-MHCII-CD115+SIRPα+ Ly6C+
BM Lin-MHCII-Flt3+SIRPαlo
B
BM Lin-
C
D
BM Lin-MHCII-
E
BM Lin-CD115+Ly6C+
F
G
H
I
J
K
DC signature
Macrophage Signature

Figure S1 (Related to Figure 1): Phenotypic characterization of R1 and R2 monocytes and R3 and P pre-DCs.

A) FACS analysis of Flt3 and CD115 in BM Ly6C^{+} cells.

Anti-Flt3 or isotype staining on MHCII^{-}CD115^{+}SIRPα^{+}Ly6C^{+} R1 and R2 monocytes and R3 pre-DCs, and anti-CD115 or isotype staining on R3 and P (Lin^{-}MHCII^{-}CD115^{-}Flt3^{+}CD11c^{+}) pre-DCs gated on Lin^{-}(Ly6G^{-}, CD3e^{-}.NK1.1^{-}, Ter119^{-}, CD19^{-}, CD45RA^{-}) cells.

B) Phenotype of MHCII^{+} cells in the BM. F4/80, CD11b and Ly6C expression

on WT BM Lin^{-}MHCII^{+} and MHCII^{-} cells.

C) Cellularity of BM subsets. Percentage of R1, R2 monocytes and R3 pre-DCs in total BM and in Lin^{-}CD115^{+}Ly6C^{+} cells and of CD115^{-}pre-DC (P) in total BM.

D) R1, R2 and R3 are not ckit^{+}.

Anti-ckit or isotype control staining of BM Lin^{-}MHCII^{-} cells. R1 and R2 monocytes and R3 pre-DCs are gated from Lin^{-}(CD3ε^{-}CD19^{-}Ly6G^{-}CD45RA^{-}NK1.1^{-}Ter119^{-}) MHCII^{-}ckit^{-}CD115^{+}Ly6C^{+} cells.

E) Phenotype of BM R1 and R2 monocytes and pre-DCs R3 and P. FACS analysis for CD11b and CCR2 (solid black lines) expression versus isotype controls (grey shaded) in WT mice. GFP reporter expression analyzed in *Cx3cr1*$^{gfp/+}$ mice at steady state. Numbers above gates indicate percentage of parent population and numbers above histograms indicate mean fluorescence intensity.

F) Giemsa staining of FACS sorted R1, R2 monocytes and R3 and P Pre-DCs from the BM of WT mice. Scale bar=10µm.

G) *Nr4a1* dependency of R1, R2, R3 and P in WT mice. Representative FACS analysis of R1 and R2 monocyte subsets and pre-DCs- R3 and P, in *Nr4a1*$^{+/+}$ and *Nr4a1*$^{-/-}$ BM.

H) Analysis of monocytes R1 and R2, and R3 pre-DCs in the blood of *Ccr2*$^{-/-}$ versus WT mice at steady state.

I) FACS analysis of *Ccr2*$^{-/-}$ and WT mixed bone marrow chimeras. FACS analysis of CD45.1^{+} (*Ccr2*$^{+/+}$) and CD45.1^{-}(*Ccr2*$^{-/-}$) cells within R1, R2 monocytes and total pre-DCs in the BM and blood, and total cDCs in the spleen of WT reconstituted recipients of *Ccr2*$^{-/-}$ and WT mixed BM (1:1).

J) Fate mapping of R1, R2, R3 and P in *Zbtb46*^{Cre} *x ROSA*^{lslYFP} mice. YFP expression in R1, R2 monocytes and pre-DCs R3 and P, in the BM and blood, and CD8α^{+} and CD11b^{+}ESAM^{+} and ESAM^{-} cDCs in the spleens of *Zbtb46*^{Cre} *x ROSA*^{lslYFP} mice.

K) Expression of cDC and macrophage ImmGen gene signatures in BM R1, R2 and R3 at steady state. Relative mRNA expression levels for each gene are depicted according to the color bar shown.

FIGURE S2

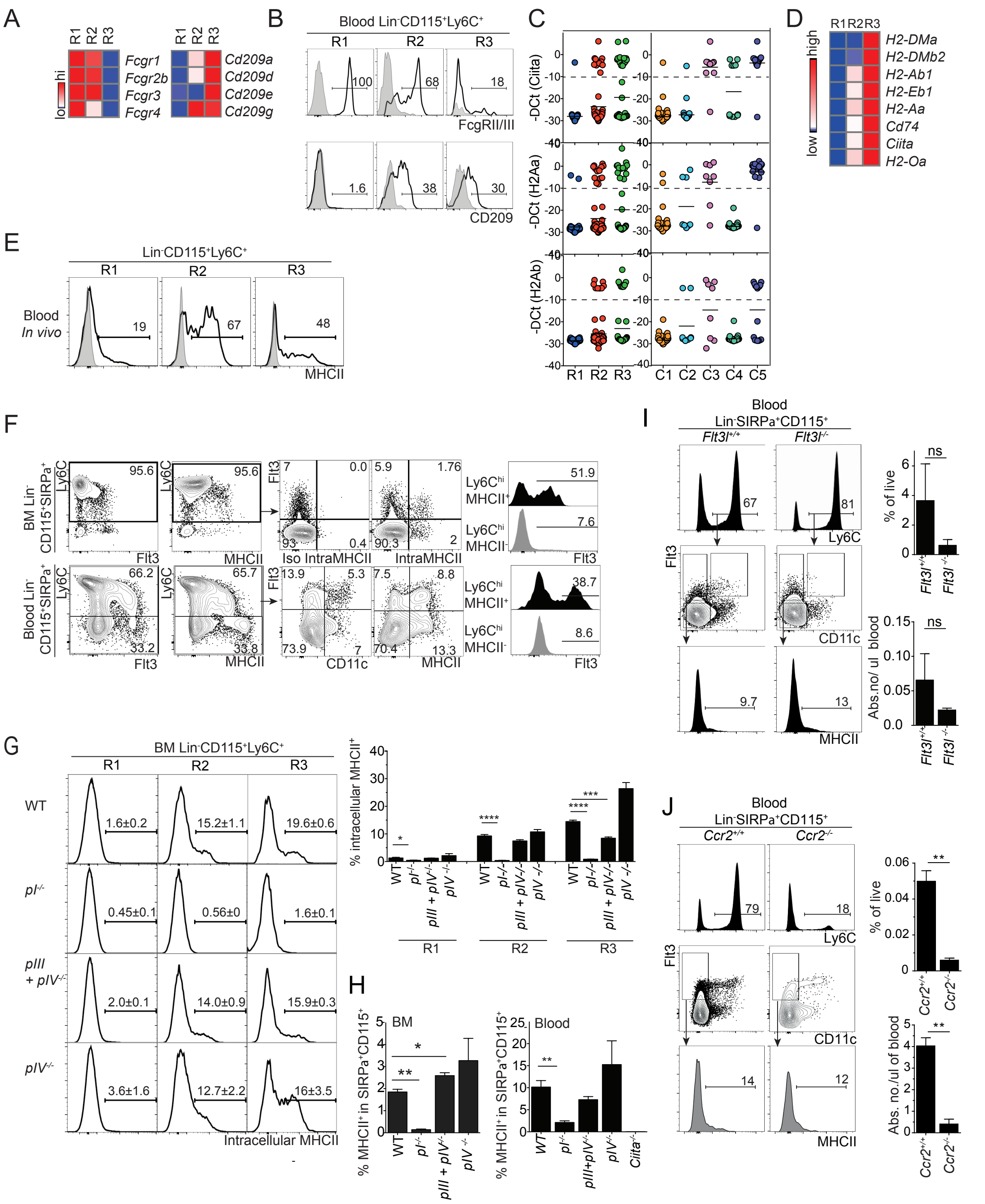

A
Fcgr1
Fcgr2b
Fcgr3
Fcgr4
Cd209a
Cd209d
Cd209e
Cd209g
B
Blood Lin-CD115+Ly6C+
R1 R2 R3
FcgRII/III
CD209
C
-DCt (Ciita)
-DCt (H2Aa)
-DCt (H2Ab)
R1 R2 R3 C1 C2 C3 C4 C5
D
H2-DMa
H2-DMb2
H2-Ab1
H2-Eb1
H2-Aa
Cd74
Ciita
H2-Oa
E
Lin-CD115+Ly6C+
Blood
In vivo
MHCII
F
BM Lin- CD115+SIRPa+
Blood Lin- CD115+SIRPa+
Iso IntraMHCII
IntraMHCII
Ly6Chi MHCII+
Ly6Chi MHCII-
G
BM Lin-CD115+Ly6C+
WT
pI-/-
pIII + pIV-/-
pIV-/-
Intracellular MHCII
% intracellular MHCII+
H
BM
Blood
% MHCII+ in SIRPa+CD115+
I
Blood
Lin-SIRPa+CD115+
Flt3l+/+
Flt3l-/-
% of live
Abs.no/ ul blood
J
Blood
Lin-SIRPa+CD115+
Ccr2+/+
Ccr2-/-
% of live
Abs. no./ul of blood

Figure S2 (Related to Figure 2): Transcriptional control of MHCII expression on R1 and R2 monocytes and R3 pre-DCs.

A,B) Expression of FcgR and CD209 in R1,R2 and R3. Average values of 3 replicates of microarray data of FcgR and CD209 in R1 and R2 monocytes and R3 pre-cDCs (A). Expression of FcgRII/III and CD209a versus respective isotype controls as assessed by FACS analysis (B).

C) -DCt values in Clusters1-5 and populations R1-3, of *Ciita, H2-Aa* and *H2-Ab* as derived from single cell multiplex qPCR analysis.

D) Microarray analysis of MHCII-related genes in R1, R2 and R3. Average values of triplicate samples in R1, R2 and R3 are shown for MHCII-related genes.

E) MHCII expression in blood $Lin^{-}CD115^{+}Ly6C^{+}$ cells at steady state.

F) MHCII expression in $Ly6C^{+}CD115^{+}$ cells in the BM and blood at steady state.

Intracellular staining of MHCII or isotype control in the BM and staining of surface MHCII in blood of WT $Lin^{-}SIRPa^{+}CD115^{+}$ cells. Histograms show the percentage of $Flt3^{+}$ cells within the Lin^{-} $SIRPa^{+}CD115^{+}Ly6C^{+}$ $MHCII^{+}$ and $MHCII^{-}$ cells.

G) Intracellular expression of MHCII in BM R1, R2 and R3 in WT, $pI^{-/-}$, $pIII{+}pIV^{-/-}$, $pIV^{-/-}$ BM at steady state. Percentages of parent indicated above each gate ± SEM.

H) MHCII expression in total BM and blood $Lin^{-}CD115^{+}SIRP\alpha^{+}$ cells in $pI^{-/-}$, $pIII^{+}pIV^{-/-}$, $pIV^{-/-}$ and $Ciita^{-/-}$ mice at steady state.

I) $MHCII^{+}$ fraction of R2 cells is independent of Flt3L. FACS analysis and quantification in percentage of live cells and absolute number of the $MHCII^{+}$ cells within R2 in the blood of $Flt3l^{-/-}$ and $Flt3^{+/+}$ mice.

J) $MHCII^{+}$ fraction of R2 cells is dependent on CCR2. FACS analysis and quantification in percentage of live cells and of $Ly6C^{+}$ monocytes of the $MHCII^{+}$ cells within R2 in $Ccr2^{-/-}$ and $Ccr2^{+/+}$ mice. Mean ± SEM, *p<0.05, **<0.005, ***p<0.0005, ****p<0.00005, Student's t-test.

FIGURE S3

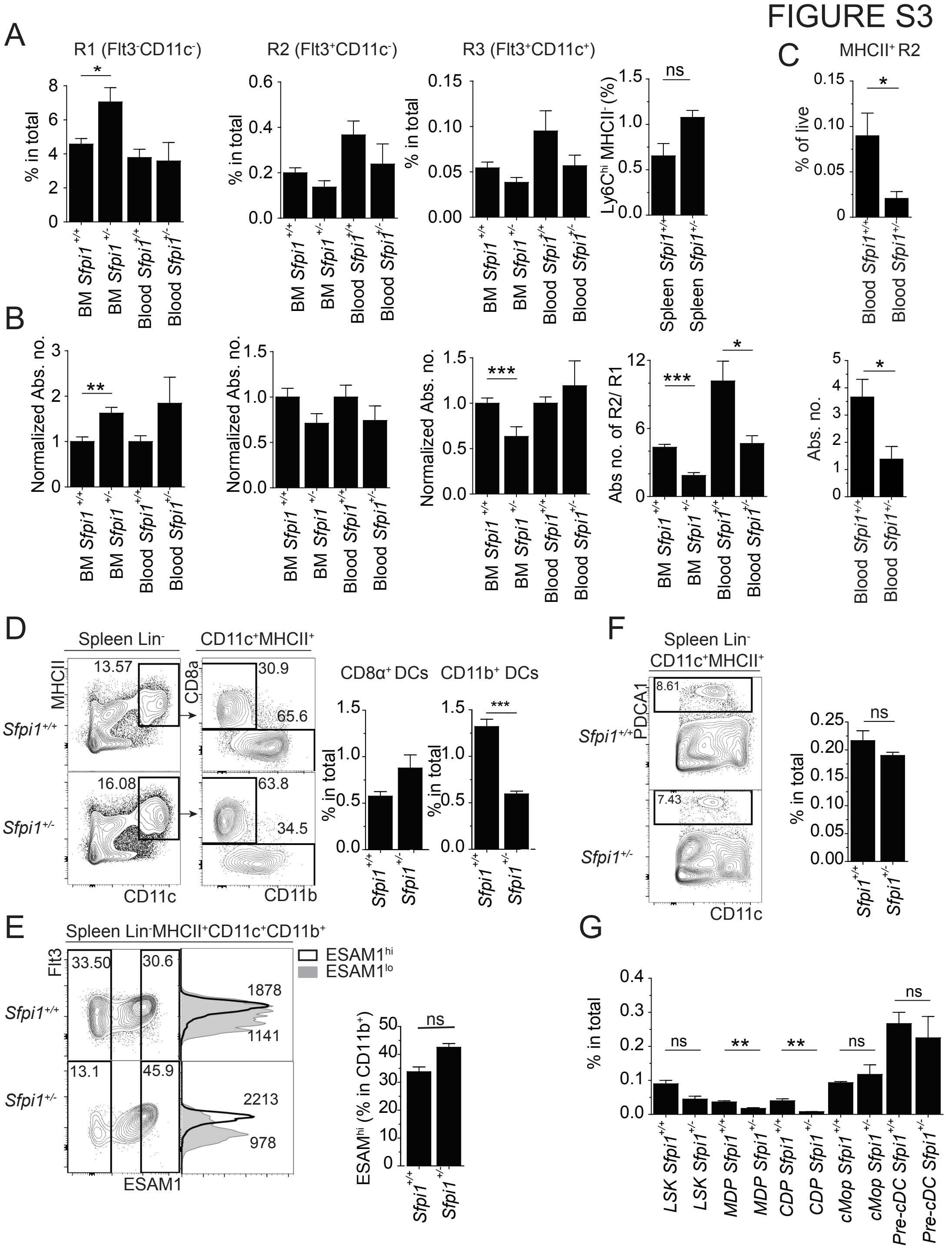

A
R1 (Flt3-CD11c-)
R2 (Flt3+CD11c-)
R3 (Flt3+CD11c+)
% in total
Ly6Chi MHCII- (%)
ns
C
MHCII+ R2
% of live
B
Normalized Abs. no.
Abs no. of R2/ R1
Abs. no.
D
Spleen Lin-
CD11c+MHCII+
MHCII
CD8a
CD11c
CD11b
13.57
16.08
30.9
65.6
63.8
34.5
CD8α+ DCs
CD11b+ DCs
F
Spleen Lin-
CD11c+MHCII+
PDCA1
8.61
7.43
CD11c
E
Spleen Lin-MHCII+CD11c+CD11b+
Flt3
ESAM1
ESAM1hi
ESAM1lo
33.50
30.6
13.1
45.9
1878
1141
2213
978
ESAMhi (% in CD11b+)
G
LSK
MDP
CDP
cMop
Pre-cDC
Sfpi1+/+
Sfpi1+/-

Figure S3 (Related to Figure 3): PU1 levels influence the development of R2 monocytes and R3 pre-DCs.

A,B) Cellularity of *Sfpi1*$^{+/+}$ and *Sfpi1*$^{+/-}$ BM, blood and spleen. Quantification of percentage in total cells (A) and normalized absolute number (normalized to *Sfpi1*$^{+/+}$ controls) (B) of BM and blood R1 and R2 monocytes and R3 pre-DCs from *Sfpi1*$^{+/+}$ and *Sfpi1*$^{+/-}$ mice. Percentage of total Ly6C^{hi}MHCII^{-} cells in total splenocytes and the fold change of absolute number of R2 over R1 monocytes in the BM and blood of *Sfpi1*$^{+/+}$ and *Sfpi1*$^{+/-}$ mice.

C) MHCII^{+} fraction of R2 is PU.1 – dependent. Percentage of live and absolute number of the MHCII^{+} cells within R2 in the blood of *Sfpi1*$^{+/+}$ versus *Sfpi1*$^{+/-}$ mice.

D-F) Cellularity of *Sfpi1*$^{+/+}$ and *Sfpi1*$^{+/-}$ spleen total DCs, CD11b^{+} DCs and pDCs.

FACS analysis of the Lin^{-}(CD19^{-}, Ly6G^{-}, CD3e^{-}, NK1.1^{-}, Ter119^{-}, CD45RA^{-}, ckit^{-}) CD11c^{+}MHCII^{+} CD8α^{+} and CD11b^{+} DCs (D) ESAM^{hi-} and ESAM^{lo-} CD11b^{+} DCs (E) and PDCA1^{+} cells (F) in the spleen of *Sfpi1*$^{+/+}$ and *Sfpi1*$^{+/-}$ mice. Histograms in D show the levels of Flt3 in the ESAM^{hi} (solid black line) and the ESAM^{lo} DCs (shaded grey) with mean fluorescence intensity (MFI) indicated adjacent to the respective histogram.

G) Percentage in total BM cells of LSK, MDP, CDP, cMop and Pre-DC populations from *Sfpi1*$^{+/+}$ and *Sfpi1*$^{+/-}$ mice.

(Data shown is mean ± SEM, *p<0.05, ***p<0.0005, ns= non-significant; Student's t-test)

FIGURE S4

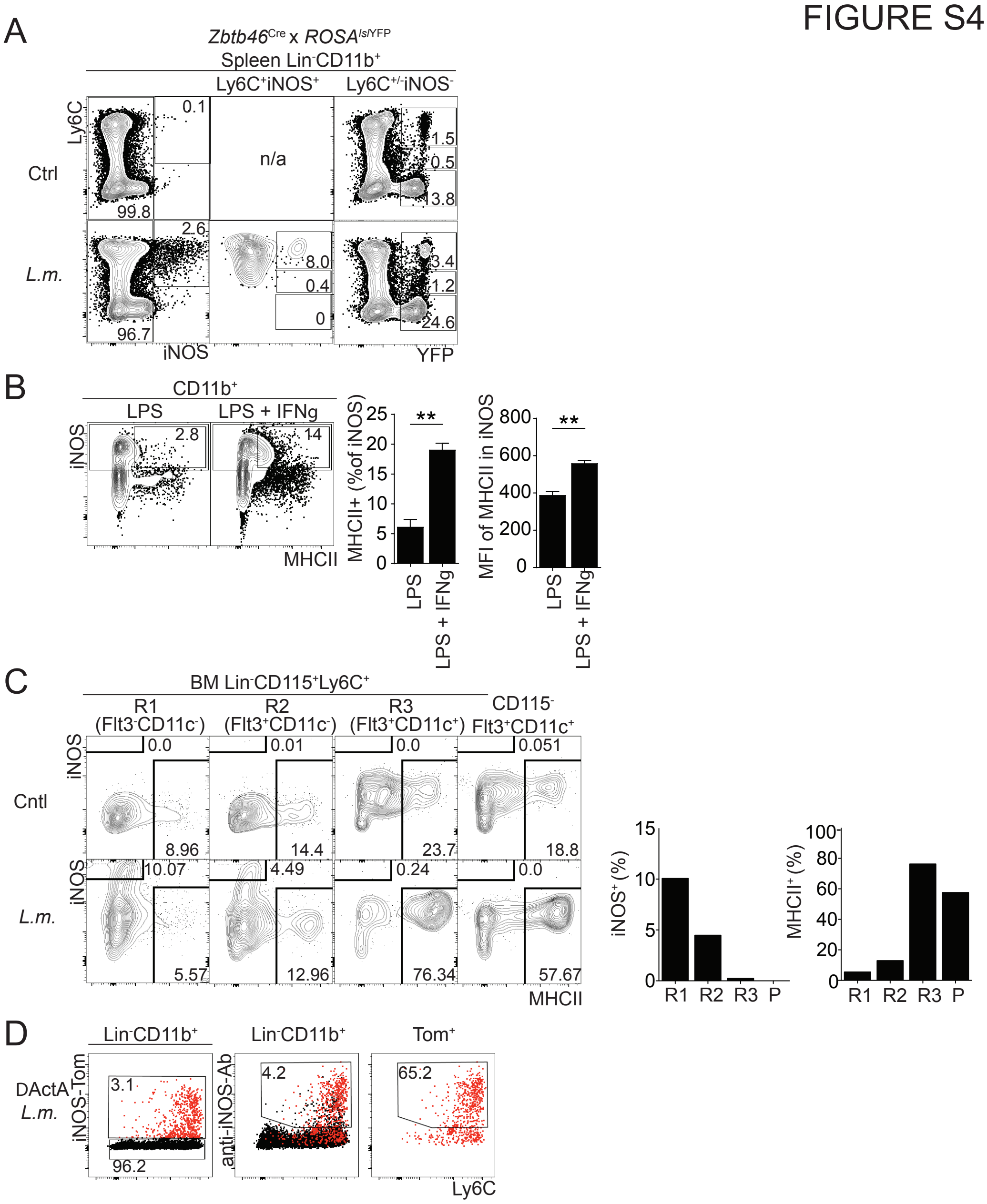
A
Zbtb46Cre x ROSAlslYFP
Spleen Lin-CD11b+
Ly6C+iNOS+
Ly6C+/-iNOS-
Ly6C
Ctrl
L.m.
0.1
99.8
n/a
1.5
0.5
3.8
2.6
96.7
8.0
0.4
0
3.4
1.2
24.6
iNOS
YFP
B
CD11b+
LPS
LPS + IFNg
iNOS
2.8
14
MHCII
MHCII+ (%of iNOS)
MFI of MHCII in iNOS
**
LPS
LPS + IFNg
C
BM Lin-CD115+Ly6C+
R1 (Flt3-CD11c-)
R2 (Flt3+CD11c-)
R3 (Flt3+CD11c+)
CD115- Flt3+CD11c+
Cntl
L.m.
iNOS
MHCII
iNOS+ (%)
MHCII+ (%)
R1 R2 R3 P
D
Lin-CD11b+
Tom+
DActA L.m.
iNOS-Tom
anti-iNOS-Ab
Ly6C
3.1
96.2
4.2
65.2

Figure S4 (Related to Figure 4): iNOS production is restricted to activated R1 monocytes.

A) DC lineage tracing of $iNOS^{+}$ cells. FACS analysis of YFP expression in $Lin^{-}CD11b^{+}$ $iNOS^{+}$ and $iNOS^{-}$ cells in *L.m.*-infected $Zbtb46^{Cre}$ *x* $ROSA^{lsl\ YFP}$ mice (day2).

B) MHCII expression in total WT BM after overnight culture with LPS, with or without IFNγ.

C) *In vitro* overnight stimulation with *L.m.* or no stimulation, of R1 and R2 monocytes and pre- DCs R3 and P.

D) Anti-iNOS antibody staining of iNOS-$Tomato^{+}$ (red cells) and total $Lin^{-}CD11b^{+}$ cells in ΔActA-*L.m.* infected $Nos2^{TomatoCRE}$*x* $ROSA^{lslTomato}$ mice.

FIGURE S5

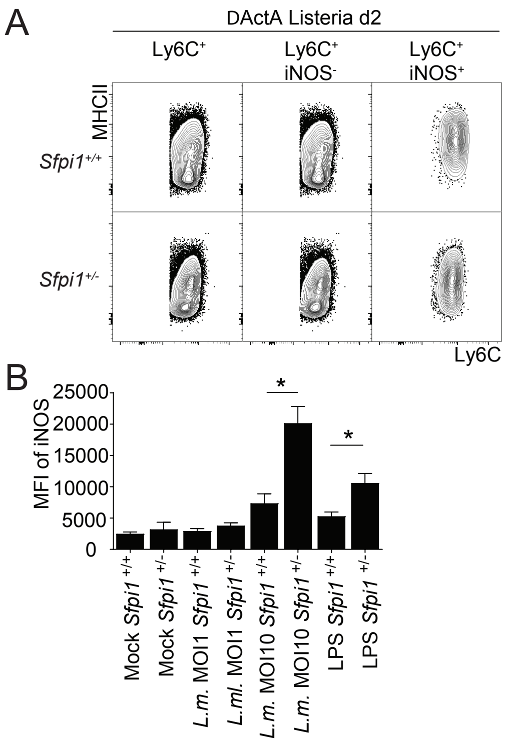

Figure S5 (Related to Figure 5): PU.1 inhibits the generation of iNOS$^+$ macrophages upon microbial stimulation.

A) MHCII expression of Ly6C$^+$, Ly6C$^+$iNOS$^-$ and Ly6C$^+$iNOS$^+$splenocytes from ΔActA-*L.m.* infected *Sfpi1*$^{+/+}$ versus *Sfpi1*$^{+/-}$ mice.

B) Mean fluorescence intensity (MFI) of anti-iNOS staining on BMDMs from *Sfpi1*$^{+/+}$ and *Sfpi1*$^{+/-}$ mice cultured overnight with plain media, *L.m.*(MOI of 1 or 10 CFU) or LPS (1ug/ml).

FIGURE S6

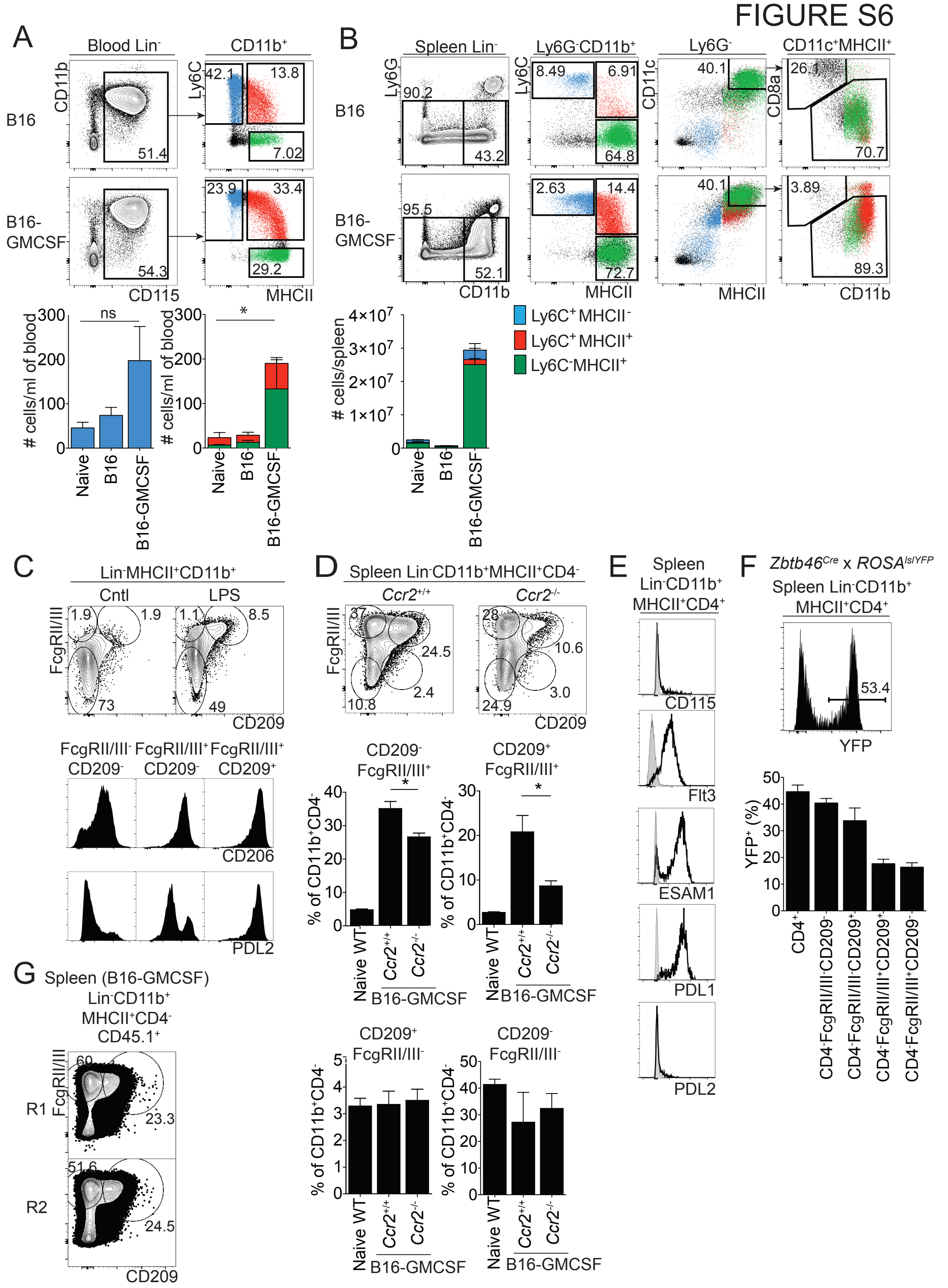
A
Blood Lin-
CD11b+
B16
B16-GMCSF
CD11b
Ly6C
CD115
MHCII
51.4
54.3
42.1
13.8
7.02
23.9
33.4
29.2
cells/ml of blood
ns
*
Naive
B16
B16-GMCSF
B
Spleen Lin-
Ly6G-CD11b+
Ly6G-
CD11c+MHCII+
Ly6G
Ly6C
CD11c
CD8a
CD11b
MHCII
90.2
43.2
8.49
6.91
64.8
40.1
26.1
70.7
95.5
52.1
2.63
14.4
72.7
3.89
89.3
cells/spleen
Ly6C+MHCII-
Ly6C+MHCII+
Ly6C-MHCII+
C
Lin-MHCII+CD11b+
Cntl
LPS
FcgRII/III
CD209
FcgRII/III- CD209-
FcgRII/III+ CD209-
FcgRII/III+ CD209+
CD206
PDL2
D
Spleen Lin-CD11b+MHCII+CD4-
Ccr2+/+
Ccr2-/-
CD209- FcgRII/III+
CD209+ FcgRII/III+
CD209+ FcgRII/III-
CD209- FcgRII/III-
% of CD11b+CD4-
Naive WT
B16-GMCSF
E
Spleen Lin-CD11b+ MHCII+CD4+
CD115
Flt3
ESAM1
PDL1
PDL2
F
Zbtb46Cre x ROSAlslYFP
Spleen Lin-CD11b+ MHCII+CD4+
53.4
YFP
YFP+ (%)
CD4+
CD4-FcgRII/III-CD209-
CD4-FcgRII/III-CD209+
CD4-FcgRII/III+CD209+
CD4-FcgRII/III+CD209-
G
Spleen (B16-GMCSF)
Lin-CD11b+ MHCII+CD4- CD45.1+
R1
R2
60
23.3
51.6
24.5
FcgRII/III
CD209

Figure S6 (related to Figure 6): PU.1^{high}Flt3^{+}MHCII^{+} R2 monocytes differentiate into PD-L2^{+}CD209^{+} moDCs upon exposure to GM-CSF

A,B) Phenotype of blood cells and splenocytes from mice bearing B16 or B16-GMCSF tumours. FACS analysis of the Ly6C^{+}MHCII^{-} (blue), Ly6C^{+}MHCII^{+} (red) Ly6C^{-}MHCII^{+} (green) cells within the Lin^{-}(Ly6G^{-}, CD19^{-}, CD3ε^{-}, NK1.1^{-}, Ter119^{-}, CD45RA^{-}, cKit^{-}) CD115^{+} cells in the blood (A) and the Lin^{-} Ly6G^{-}CD11b^{+} cells in spleen (B) of B16 or B16-GMCSF tumor-bearing mice. FACS staining for CD11c and MHCII in Lin^{-}Ly6G^{-} cells of the spleen showing the overlap of Ly6C^{+}MHCII^{-} (blue), Ly6C^{+}MHCII^{+} (red) and Ly6C^{+}MHCII^{+} (green) cells within the DC gating (B). n= 3-6 mice per group (A,B) (Mean ± SEM;* $p<0.05$, ns= non-significant; Student's unpaired t-test.)

C) Phenotype of lymph nodes after LPS treatment. FACS analysis for FcɣRII/III and CD209a expression in the Lin^{-}MHCII^{-}CD11b^{+} cells of mice treated either with PBS or LPS. CD206 and PDL2 phenotype of the FcɣRII/III^{+}CD209^{+}, FcɣRII/III^{+}CD209^{-} and the FcɣRII/III^{-}CD209^{-} cells is shown.

D) Role of CCR2 in GM-CSF-dependent CD11b^{+}MHCII^{+} splenocytes.

FACS analysis of FcgRII/III^{+}CD209^{-}, FcgRII/III^{+}CD209^{+}, FcgRII/III^{-}CD209^{+} and FcgRII/III^{-}CD209^{-} in WT and *Ccr2*$^{-/-}$ mice bearing B16-GMCSF tumors. Percentage in the Lin^{-}CD11b^{+}MHCII^{+}CD4^{-} splenocytes of each subset is shown. Mean ± SEM, * $p<0.05$, Student's unpaired t-test.

E) Phenotype of naïve CD4^{+} DCs in the spleen. FACS staining of Lin^{-}CD11b^{+}MHCII^{+}CD4^{+} cells for CD115, Flt3, ESAM1, PDL1 (CD274) and PDL2 (CD273) in naïve mice.

F) *Zbtb46*^{Cre} *x ROSA* ^{lslYFP} fate mapping. FACS analysis of YFP expression on Lin^{-}CD11b^{+}MHCII^{+}CD4^{+} cells from naïve *Zbtb46*^{Cre} *x ROSA* ^{lslYFP}mice. Percentage of YFP^{+} cells within Lin^{-}CD11b^{+}MHCII^{+} subsets from B16-GMCSF-tumour-engrafted *Zbtb46*^{Cre} *x ROSA* ^{lslYFP}mice.

G) Endogenous expression of FcɣRII/III and CD209a in Lin^{-}CD11b^{+}MHCII^{+}CD4^{-} cells in the tumour-bearing WT (CD45.1) mice adoptively transferred with R1 (CD45.2) or R2 (CD45.2).

FIGURE S7

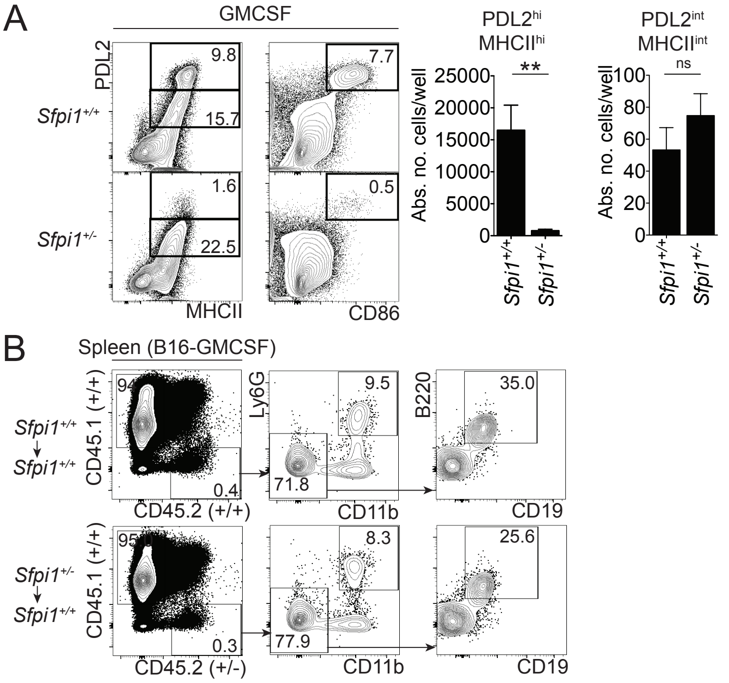

Figure S7 (Related to Figure 7): PU1 influences the number of the PDL2$^+$MHCII$^+$ cells generated *in vitro* in response to GMCSF.

A) GM-CSF culture of total BM from *Sfpi1*$^{+/+}$ and *Sfpi1*$^{+/-}$ mice.

FACS analysis for PDL2 (CD273), MHCII and CD86 from DAPI$^-$ progeny obtained after 5 days of GMCSF culture. Absolute number of PDL2$^+$MHCII^{hi} and PDL2^{int}MHCII^{int} cells is quantified in the graphs shown. n=4 independent cultures.

B) Phenotype of adoptively transferred *Sfpi1*$^{+/+}$ or *Sfpi1*$^{+/-}$ whole BM into B16-GMCSF treated CD45.1$^+$ congenic recipients. FACS analysis of CD45.1$^+$ (recipient) and CD45.2$^+$ (donor) granulocytes (Ly6G$^+$CD11b$^+$) and B cells (Ly6G$^-$CD11b$^-$CD19$^+$ B220$^+$) in B16-GMCSF tumor engrafted CD45.1$^+$ mice.

C) Graphical summary of proposed model.

Ly6C$^+$ monocytes consist of 2 subsets- PU1^{lo} R1 and PU1^{hi} R2, that are independently capable of developing into different inflammatory progeny. R1 gives rise to FcɣRII/III$^+$CD209a$^-$ cells in response

to GM-CSF or $iNOS^+$ cells in response to microbial stimulation. R2 produces $FcgRII/III^+CD209^+$ mo-DCs in response to GM-CSF. These different roles are dictated by increased or decreased levels of PU.1 expression.

Supplemental Experimental Procedures

Mice: C57Bl/6 mice were bought from Charles River Laboratories, UK. *Ccr2*$^{-/-}$ (Boring et al., 1997), *Nr4a1*$^{-/-}$ (Lee et al., 1995), *Sfpi1*$^{+/-}$ (McKercher et al., 1996), CD45.1^{+} (JAX:002014) mice were housed within the clean unit of the animal housing facility at King's College London. *Zbtb46-GFP* mice (Satpathy et al., 2012)were housed at the animal facility at Queen Mary, University of London. *pIII +pIV*$^{-/-}$: (LeibundGut-Landmann et al., 2004), *pIV*$^{-/-}$ (Waldburger et al., 2001), *pI*$^{-/-}$ (Dubrot et al., 2014) and *Ciita*$^{-/-}$ (JAX: 003239) micewere housed at Geneva University, Switzerland. All mice maintained under specific pathogen-free conditions in accordance with the UK Animals (Scientific Procedures) Act,1986. *Zbtb46*^{Cre} *x ROSA*^{loxSTOPloxYFP} (Jakob Loshk, 2016) , *NOS2*$^{Tomato\text{-}Cre}$*xROSA*^{loxSTOPloxtdTomato} (Bechade et al., 2014) bone marrow was injected into lethally irradiated recipients and used in all experiments as indicated.

Reagents: Complete medium used for cell culture was RPMI (Life technologies) with Glutamax, and 10% FBS (Life technologies) and 50uM beta-mercaptoethanol (Sigma). FACS buffer used was made of PBS (Life Technologies) with 1% bovine serum albumin (Apollo Scientific) and 2mM EDTA.

Bone marrow Chimeras: 8-10 week old C57Bl/6 mice were hematopoietically-lethal irradiated with 11Gy; bone marrow from *Zbtb46-Cre-loxSTOPloxYFP* (Loshko *et al*, 2016) , *Zbtb46-iDTR* mice (Meredith et al., 2012)or *NOS2-*$^{Tomato\text{-}Cre}$*xROSA*^{loxSTOPloxtdTomato} (Bechade et al., 2014) was injected intravenously at a dose of 5 x 10^{6} cells in100ul into these irradiated recipients. To allow full reconstitution, the mice were used at 8-16 weeks after transplantation. *Ccr2*$^{-/-}$ (CD45.2+) and WT (CD45.1+) mixed bone marrow chimeras were

produced in hematopoietically-lethal irradiated (11Gy) CD45.1+ recipients that received CD45.1$^+$ WT and *Ccr2*$^{-/-}$ (CD45.2$^+$) bone marrow in equal parts. CD45.1+ recipients that received 100%CD45.2+ C57Bl/6 bone morrow were used as a control for the complete replacement of recipient bone marrow with donor-derived bone marrow.

FACS Reagents: Fluorochrome or biotin- conjugated antibodies were used to stain single cell suspensions for flow cytometry. These included monoclonal antibodies specific to mouse CD19 (Clone MB19-1), Ly6G (Clone: 1A8),CD3ε (Clone 145-2C11), Ter119 (Clone Ter119), NK1.1 (Clone PK136), CD45RA (Clone: HI100), ckit (Clone:2B8), MHCII I-A/I-E (Clone: M5/114. 15.2), Ly6C (clone: HK1.4), CD11b (clone:M1/70), CD115 (clone:AFS98), CD16/32 (clone: 93), CD209 (clone: LW206), CD135 (clone A2F10), CD172a (clone: P84), CD11c (clone: N418), CD273 (clone: TY25), CD274 (clone:10F.9G2) that were bought from eBioscience (Hatfield, UK) and Biolegend (London, UK). For staining intracellular levels of iNOS, cells were fixed and permeabilized using BD Cytofix/Cytoperm Fixation kit as per manufacturer's instructions and stained with anti-iNOSA488 or -iNOS-PE (clone: CXNFT) purchased from eBioscience. For staining PU.1, cells were fixed and permeabilized using FoxP3/ Transcription factor staining buffer set (eBioscience) according to manufacturer's instructions. Anti-PU.1 rabbit monoclonal antibody (clone: 9G7) and the corresponding isotype were purchased from Cell Signaling Technology (New England Biolabs (UK) Ltd).

Cell sorting by flow cytometry

For sorting, bone marrow or spleen cells were resuspended to an approximate concentration of 7000cells/ul. They were then sorted on a BD FACS Aria (special order machine) fitted with 405nm, 488nm, 561nm, 633nm lasers and sorted through 100um nozzle with 4-way purity. Purity checks were run on samples used for microarrays and were used when purity

was found to be >95%. Both instruments were housed at the Biomedical Research Centre Flow Core Facility (Guy's and St Thomas' NHS Foundation Trust and King's College London). Flow cytometry analysis was done using FlowJo software (TreeStar).

Giemsa Staining: Cytospins of FACS sorted R1, R2, R3 and P were fixed with methanol for 5 mins, stained with 1:20 Giemsa stain in deionized water for 45 mins and then washed and air dried. Slides were imaged on Motic AE2000 with 40x magnification. Images were modified for brightness with ImageJ (NIH).

***In vitro L.m.* infections**: *In vitro* cultures of primary sorted cells with *Listeria monocytogenes* (*L.m.)* was done overnight at an MOI of 0.01 or 0.1 as indicated in complete RPMI1640 medium supplemented with MCSF (20ng/ml) (Peprotech), GMCSF(3ng/ml) (Peprotech) and human Flt3L(100ng/ml) (CellDex). BMDMs were derived from *Sfpi1*$^{+/-}$ or *Sfpi1*$^{+/+}$ BM cultured for 8-10 days in DMEM medium (Life technologies) supplemented with 10% FBS and 10% MCSF containing L-929 cell culture supernatant. These were re-plated as 0.45 x 10^6 cells/well in a 24-well non-tissue culture treated plate to be stimulated with LPS (1ug/ml) or *L.m.* at MOI 1 or 10 for 16 hours. Cells were collected, stained with fluorochrome conjugated antibodies and analysed by FACS.

B16-GMCSF tumor experiments: Melanoma cell lines B16 and B16 expressing GMCSF (B16-GMCSF)(Dranoff et al., 1993) were maintained in RPMI1640 medium supplemented with Glutamax (Life technologies), 10% fetal bovine serum (Life technologies), 1% penicillin-streptomycin (Life Techonologies) and 50uM beta-mercaptoethanol (Sigma) and used from between passages 4 and 10. Cells were checked for viability with Trypan Blue and 1.5-3 x 10^5 live cells were injected subcutaneously in sterile RPMI 1640 medium alone.

Infection. *Listeria monocytogenes* (*L.m.*) or the ΔActA mutant of the same (ΔActA *Listeria)* were grown and sub-cultured in brain heart infusion broth at 37^0C until an OD_{600} value of 0.12-0.15 was obtained to use bacteria in their exponential growth phase. 4 – 5 x 10^3 wild type CFU (Listeria) or 10^6 ΔActA mutant CFU of *Listeria monocytogenes* were injected intravenously in sterile PBS.

Microarray processing and analysis:

Affymetrix CEL files were converted into gct files using the ExpressionFileCreator Module within Gene Pattern Software (Broad Institute) (Reich et al., 2006). The RMA algorithm withquantile normalization and background correction was used. No thresholds or filters were applied for assessing the relative expression of all genes assayed on the microarray. Heat maps were generated with this data on Gene-E software.

To create the Volcano plots, Mutiplot Preprocess Module within the Gene Pattern Software (Broad Institute) was used to derive fold change and p-values from the expression dataset of the aforementioned microarrays to be used in the MultiplotVisualizer Module. This latter module was used to highlight the genes more highly expressed in R2 or R3 above a threshold of p-value set at 0.05 and fold change of 1.2. These selected genes were then overlaid on comparisons done between R1 and R2, and R1 and R3 to obtain the plots shown in Fig.1F.

PCA analysis and hierarchical clustering: Microarray data of R1, R2 and R3 were compared with ST1.0 array data available on ImmGEN for Pre-DCs (GSE68590)) (Tussiwand et al., 2015) (and CDP (GSE 15907) (www.immgen.org) on Qlucore Omics Explorer (Sweden) and plotted as 2D plots on Prism (Graphpad). Hierarchical clustering of data sets was performed using Gene-E software (Broad Institute).

qPCR Primers: Cells were sorted as described and centrifuged. Supernatant was removed and cells were resuspended in RLT buffer from the RNeasy kit (Qiagen). mRNA was extracted using the columns as per manufacturer's instructions. mRNA was resuspended in RNase-free water and the concentration and quality measured by nanodrop (Thermo Scientific). Equal amounts of mRNA (between 0.1ng – 5ug) from each sample were taken to produce cDNA using the manufacturer's First Strand cDNA synthesis protocol with the RevertAid™ H minus Reverse Transcriptase (Thermo Scientific). Random Primers (Oligo dT primers) were mixed with template RNA and incubated at 65°C for 5 mins and Ribolock (Thermo Scientific), dNTP mix, Reaction buffer (5x) and mMulV reverse transcriptase enzyme were added and incubated at RT for 10 mins, 42°C for 1hr and at 70°C for 5 mins to inactivate the enzyme. A 1 in 10 dilution of this cDNA was used to perform qPCR with Sensimix™SYBR® (Bioline) as per manufacturer's instructions.

Multidimensional reduction analysis: Automated t-distributed stochastic non-linear embedding (t-SNE) algorithm was used to visually (viSNE) analyse (Amir el et al., 2013) bone marrow monocytes acquired by FACS for 7 fluorochromes. The online (web-based) software implementation of viSNE (Cytobank) (Kotecha et al., 2010) was used to analyse the presence of different populations within the Ly6C^{hi}CD115^{+} BM monocytes. No *a priori* gating was used and an unbiased automated analysis was conducted. The resulting viSNE maps were overlaid with each monocyte population, R1, R2 and pre-DC R3 that were gated separately by conventional FACS analysis. The colour scheme for all four viSNE maps was adjusted to represent the Flt3 expression – blue colour denoting lower levels and red – higher.

Single cell qPCR: Single cells were FACS sorted from bone marrow into 9ul of Cell Direct pre-Amp master mix in 96-well qPCR plates. Complementary DNA (cDNA) synthesis and specific target amplification of 45 genes (including 3 housekeeping genes- Hprt, ActB, Gapdh) was performed using CellsDirect One Step qRT-PCR kit (Invitrogen) with 48 Taqman assays (Life technologies) at 0.2x. Reverse transcription was performed within the same plates using the following cycle: 40^0C for15 mins, 50^0C for 15 mins, 60^0C for 15 mins. Enzyme inactivation was done at 95^0C for 2mins followed by 22x (95^0C for 15s, 60^0C for 4mins). cDNA was then diluted 1 in 5 in low EDTA TE buffer. Samples were stored at -20^0C until used in the BioMark. 81 cells of R2, and 44 cells of R1 and R3 each were compared along with control well including 10 cells and no cell controls and dilutions of cDNA from 10^5 cells to 1 cells to check for primer viability. 5ul of diluted cDNA + Taqman mastermix + Sample loading reagent and 5 ul of each Taqman assay + Assay loading reagent were loaded into their respective wells on 4 M48.M48 Dynamic Arrays. Samples and Assays were then loaded into the reaction chambers of the Dynamic Array using the IFC ControllerMX (Fluidigm), and then transferred to the BioMark HD for qPCR (95˚C for 10 min; 40 cycles of 95˚C for 15 s and 60 ˚C for 60 s). Data obtained from the 'Real time PCR analysis' software (Fluidigm) was analysed using Gene-E software.

The following Taqman probes were tested on all samples.

	Primers
Adamts3	Mm00625880_m1
Cd209a	Mm00460067_m1
Cd209c	Mm00652419_m1
Cd209d	Mm00459972_m1
Cd209e	Mm00459980_m1
Cd74	Mm01262763_m1
Ciita	Mm00482914_m1
Clec10a	Mm00546124_m1
Clec4a2	Mm00488795_m1
Clec4g	Mm01212425_m1
Clec5a	Mm01131766_m1
Clec7a	Mm00490960_m1
Clec9a	Mm00554956_m1
Csf1r	Mm01266652_m1
Csf2ra	Mm00438331_g1
Csf2rb	Mm00655745_m1
Csf3r	Mm00432735_m1
Ctsb	Mm01310506_m1
CtsG	Mm00456011_m1
Cybb	Mm01287743_m1

Fcgr2b	Mm00438879_m1
Fcgr3	Mm00438882_m1
Fcgr4	Mm00519988_m1
Flt3	Mm00439016_m1
H2-Aa	Mm00439211_m1
H2-Ab1	Mm01271199_m1
H2-dma	Mm04337015_m1
H2-dMb2	Mm00783707_s1
Id2	Mm00711781_m1
Irf8	Mm00492567_m1
Klrb1f	Mm04211785_m1
Klrd1	Mm00495182_m1
Kmo	Mm01321343_m1
Mgl2	Mm01250813_m1
Mpo	Mm01298424_m1
Mrc1	Mm00485155_m1
Ms4a3	Mm00460072_m1
Nfil3	Mm00600292_s1
Sfpi1	Mm00488140_m1
Spib	Mm03048233_m1
Tcfec	Mm01161234_m1
Zbtb46	Mm00511327_m1

These genes were selected for their discriminating capacity based on the PCA analysis between sorted populations of R1, R2 and R3 along with genes with previously described expression in monocytes and DCs.

Statistical Analysis. Data was analysed for statistical significance by unpaired Student's t-test. Differences were considered significant for $p<0.05$. *$p<0.05$, **$p<0.005$, ***$p<0.0005$, ****$p<0.00005$.

Supplementary References:

Amir el, A.D., Davis, K.L., Tadmor, M.D., Simonds, E.F., Levine, J.H., Bendall, S.C., Shenfeld, D.K., Krishnaswamy, S., Nolan, G.P., and Pe'er, D. (2013). viSNE enables visualization of high dimensional single-cell data and reveals phenotypic heterogeneity of leukemia. Nat Biotechnol *31*, 545-552.

Bechade, C., Colasse, S., Diana, M.A., Rouault, M., and Bessis, A. (2014). NOS2 expression is restricted to neurons in the healthy brain but is triggered in microglia upon inflammation. Glia *62*, 956-963.

Boring, L., Gosling, J., Chensue, S.W., Kunkel, S.L., Farese, R.V., Jr., Broxmeyer, H.E., and Charo, I.F. (1997). Impaired monocyte migration and reduced type 1 (Th1) cytokine responses in C-C chemokine receptor 2 knockout mice. J Clin Invest *100*, 2552-2561.

Dranoff, G., Jaffee, E., Lazenby, A., Golumbek, P., Levitsky, H., Brose, K., Jackson, V., Hamada, H., Pardoll, D., and Mulligan, R.C. (1993). Vaccination with irradiated tumor cells engineered to secrete murine granulocyte-macrophage colony-stimulating factor stimulates potent, specific, and long-lasting anti-tumor immunity. Proc Natl Acad Sci U S A *90*, 3539-3543.

Dubrot, J., Duraes, F.V., Potin, L., Capotosti, F., Brighouse, D., Suter, T., LeibundGut-Landmann, S., Garbi, N., Reith, W., Swartz, M.A., *et al.* (2014). Lymph node stromal cells acquire peptide-MHCII complexes from dendritic cells and induce antigen-specific CD4(+) T cell tolerance. J Exp Med *211*, 1153-1166.

Kotecha, N., Krutzik, P.O., and Irish, J.M. (2010). Web-based analysis and publication of flow cytometry experiments. Curr Protoc Cytom *Chapter 10*, Unit10 17.

Lee, S.L., Wesselschmidt, R.L., Linette, G.P., Kanagawa, O., Russell, J.H., and Milbrandt, J. (1995). Unimpaired thymic and peripheral T cell death in mice lacking the nuclear receptor NGFI-B (Nur77). Science *269*, 532-535.

LeibundGut-Landmann, S., Waldburger, J.M., Reis e Sousa, C., Acha-Orbea, H., and Reith, W. (2004). MHC class II expression is differentially regulated in plasmacytoid and conventional dendritic cells. Nat Immunol *5*, 899-908.

McKercher, S.R., Torbett, B.E., Anderson, K.L., Henkel, G.W., Vestal, D.J., Baribault, H., Klemsz, M., Feeney, A.J., Wu, G.E., Paige, C.J., *et al.* (1996). Targeted disruption of the PU.1 gene results in multiple hematopoietic abnormalities. EMBO J *15*, 5647-5658.

Meredith, M.M., Liu, K., Darrasse-Jeze, G., Kamphorst, A.O., Schreiber, H.A., Guermonprez, P., Idoyaga, J., Cheong, C., Yao, K.H., Niec, R.E., *et al.* (2012). Expression of the zinc finger transcription factor zDC (Zbtb46, Btbd4) defines the classical dendritic cell lineage. J Exp Med *209*, 1153-1165.

Reich, M., Liefeld, T., Gould, J., Lerner, J., Tamayo, P., and Mesirov, J.P. (2006). GenePattern 2.0. Nature genetics *38*, 500-501.

Satpathy, A.T., Wumesh, K.C., Albring, J.C., Edelson, B.T., Kretzer, N.M., Bhattacharya, D., Murphy, T.L., and Murphy, K.M. (2012). Zbtb46 expression distinguishes classical dendritic cells and their committed progenitors from other immune lineages. Journal of Experimental Medicine *209*, 1135-1152.

Tussiwand, R., Everts, B., Grajales-Reyes, G.E., Kretzer, N.M., Iwata, A., Bagaitkar, J., Wu, X., Wong, R., Anderson, D.A., Murphy, T.L., *et al.* (2015). Klf4 expression in conventional dendritic cells is required for T helper 2 cell responses. Immunity *42*, 916-928.

Waldburger, J.M., Suter, T., Fontana, A., Acha-Orbea, H., and Reith, W. (2001). Selective abrogation of major histocompatibility complex class II expression on extrahematopoietic cells in mice lacking promoter IV of the class II transactivator gene. J Exp Med *194*, 393-406.
